# Supplementary material for: STING induces HOIP-mediated synthesis of M1 ubiquitin chains to stimulate NF-κB signaling
Source: EMBO J. 2024 Nov 22;44(1):141–65. doi: 10.1038/s44318-024-00291-2 (PMC11696098; doi:10.1038/s44318-024-00291-2)
Supplement: Supplementary file 1 — Appendix [file 44318_2024_291_MOESM1_ESM.pdf]

Appendix

STING induces HOIP-mediated synthesis of M1 ubiquitin chains to stimulate NF-kB signaling

Tara D. Fischer, Eric N. Bunker, Peng-Peng Zhu, François Le Guerroué, Mahan Hadjian, Eunice Dominguez-Martin, Francesco Scavone, Robert Cohen, Tingting Yao, Yan Wang, Achim Werner, and Richard J. Youle

Table of Contents:

Appendix Table S1………..Page 2
Appendix Table S2………..Page 2
Appendix Table S3………..Page 3
Appendix Figure S1………Pages 4-6
Appendix Figure S2………Pages 7-8

Appendix Table S1. qPCR Primer Sequences

Gene	F/R Primer Sequences
Hu *IFNB1*	F: CTTGGATTCCTACAAAGAAGCAGC
	R: TCCTCCTTCTGGAACTGCTGCA
Hu *IFIT3*	F: CGGAACAGCAGAGACACAGA
	R: CGGAACAGCAGAGACACAGA
Hu *ISG15*	F: TGAGAGGCAGCGAACTCATC
	R: CAGCATCTTCACCGTCAGGT
Hu *TNF*	F: CTCTTCTGCCTGCTGCACTTTG
	R: ATGGGCTACAGGCTTGTCACTC
Hu *TNFAIP3*	F: GAAGCTTGTGGCGCTGAAAA
	R: GAACGCCCCACATGTACTGA
Hu *IL6*	F: AAGCCAGAGCTGTGCAGATG
	R: GCATTTGTGGTTGGGTCAGG
Hu *ACTB*	F: GCACTCTTCCAGCCTTCCTT
	R: AATGCCAGGGTACATGGTGG
Ms *Ifnb1*	F: GCCTTTGCCATCCAAGAGATGC
	R: ACACTGTCTGCTGGTGGAGTTC
Ms *Ifit3*	F: GCTCAGGCTTACGTTGACAAGG
	R: CTGCAAGTGCATCATCGTTGTTC
Ms *Isg15*	F: CATCCTGGTGAGGAACGAAAGG
	R: CTCAGCCAGAACTGGTCTTCGT
Ms *Tnf*	F: GGTGCCTATGTCTCAGCCTCTT
	R: GCCATAGAACTGATGAGAGGGAG
Ms *Tnfaip3*	F: AGCAAGTGCAGGAAAGCTGGCT
	R: GCTTTCGCAGAGGCAGTAACAG
Ms *Il6*	F: TACCACTTCACAAGTCGGAGGC
	R: CTGCAAGTGCATCATCGTTGTTC
Ms *Actb*	F: CATTGCTGACAGGATGCAGAAGG
	R: TGCTGGAAGGTGGACAGTGAGG

Appendix Table S2. CRISPR gRNA Information

Cell Line	Gene	gRNA	Transcript ID	Exon
HOIPKO HeLa	*RNF31*	(5'-GAATACTCATCCAAGACAGC-3')	ENST00000324103.11	Exon 4
		(3'-GCTGTCTTGGATGAGTATTC-5')		
HOIPKO^{pool} THP1	*RNF31*	UGCAGGUAGCGGGCGGCUAG	ENST00000324103.11	Exon 1
		CCUGAGGAGGGAUUCCGGGC		
		CAGGAUGCCGGGGGAGGAAG		
ATG16L1KO THP1	*ATG16L1*	GACAAUGUGGAUACUCAUCC	ENST00000392018.1	Exon 10

Appendix Table S3. shRNA Target Information

Cell Line	shRNA target sequence	Gene	Transcript ID
shHOIP iBMDMs	GCCACTATTCGCTACCTACAT	*RNF31*	ENSMUST00000019443.15

Appendix Figure S1

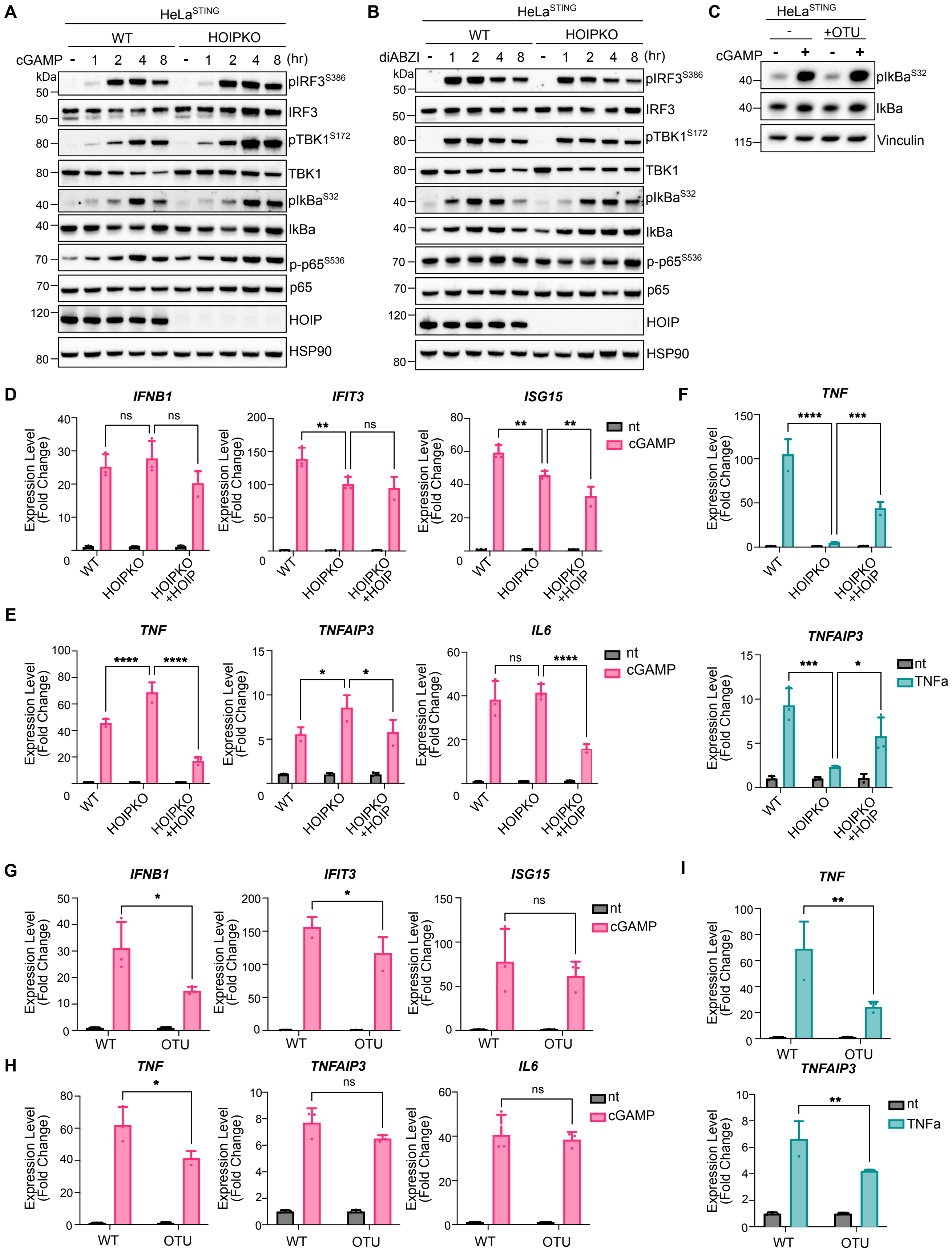

Appendix Figure S1.

A-B) Representative immunoblots of indicated proteins detected in WT and HOIPKO HeLa cell lysates prepared following treatment with 120 µg/mL cGAMP (A) or 1 µM diABZI (B) for 1, 2, 4, and 8 hours. Immunoblotting was replicated in 3 independent experiments.

C) Representative immunoblots of indicated proteins detected in lysates from $HeLa^{STING}$ and $HeLa^{STING}$ cells with stable overexpression of mEGFP-OTULIN prepared following treatment with 120 µg/mL cGAMP for 8 hours. Immunoblotting was replicated in 3 independent experiments.

D-E) Relative expression of indicated NFκB-related genes (D) and IRF3/interferon-related genes (E) detected by quantitative RT-PCR in $HeLa^{STING}$: WT, HOIPKO, and HOIPKO stably expressing mEGFP-HOIP cells treated with 120 µg/mL cGAMP for 8 hours. Quantification of relative expression is from 3 independent experiments analyzed at the same time. A 2-way ANOVA with a Tukey's multiple comparisons test was performed on $2^{-\Delta\Delta Ct}$ values. Error bars represent Standard Deviation. *<0.05, **<0.01, ***<0.001, ****<0.0001**F)** Relative expression of indicated NFκB-related genes detected by quantitative RT-PCR in $HeLa^{STING}$ WT,HOIPKO, and HOIPKO stably expressing mEGFP-HOIP cells treated with 10 ng/mL TNFα for 30 minutes. Quantification of relative expression is from 3 independent experiments analyzed at the same time. A 2-way ANOVA with a Tukey's multiple comparisons test was performed on $2^{-\Delta\Delta Ct}$ values. Error bars represent Standard Deviation. *<0.05, **<0.01, ***<0.001, ****<0.0001.

F) Relative expression of indicated NFκB-related genes detected by quantitative RT-PCR in $HeLa^{STING}$: WT, HOIPKO, and HOIPKO stably expressing mEGFP-HOIP cells treated with 10 ng/mL TNFα for 30 minutes. Quantification of relative expression is from 3 independent experiments analyzed at the same time. A 2-way ANOVA with a Tukey's multiple comparisons test was performed on $2^{-\Delta\Delta Ct}$ values. Error bars represent Standard Deviation. *<0.05, **<0.01, ***<0.001, ****<0.0001.

G-H) Relative expression of indicated NFκB-related genes (G) and interferon-related genes (H) detected by quantitative RT-PCR in WT $HeLa^{STING}$ and WT $HeLa^{STING}$ cells stably overexpressing mEGFP-OTULIN treated with 120 µg/mL cGAMP for 8 hours. Quantification of relative expression is from 3 independent experiments analyzed at the same time. A 2-way ANOVA with a Tukey's multiple comparisons test was performed on $2^{-\Delta\Delta Ct}$ values. Error bars represent Standard Deviation. *<0.05, **<0.01, ***<0.001, ****<0.0001.

I) Relative expression of indicated NFκB-related genes detected by quantitative RT-PCR in $HeLa^{STING}$ and $HeLa^{STING}$ cells with stable overexpression of mEGFP-OTULIN treated with 10 ng/mL TNFα for 30 minutes. Quantification of relative expression is from 3 independent experiments analyzed at the same time. A 2-way ANOVA with a Tukey's multiple comparisons test was performed on $2^{-\Delta\Delta Ct}$ values. Error bars represent Standard Deviation. *<0.05, **<0.01, ***<0.001, ****<0.0001.

Appendix Figure S2

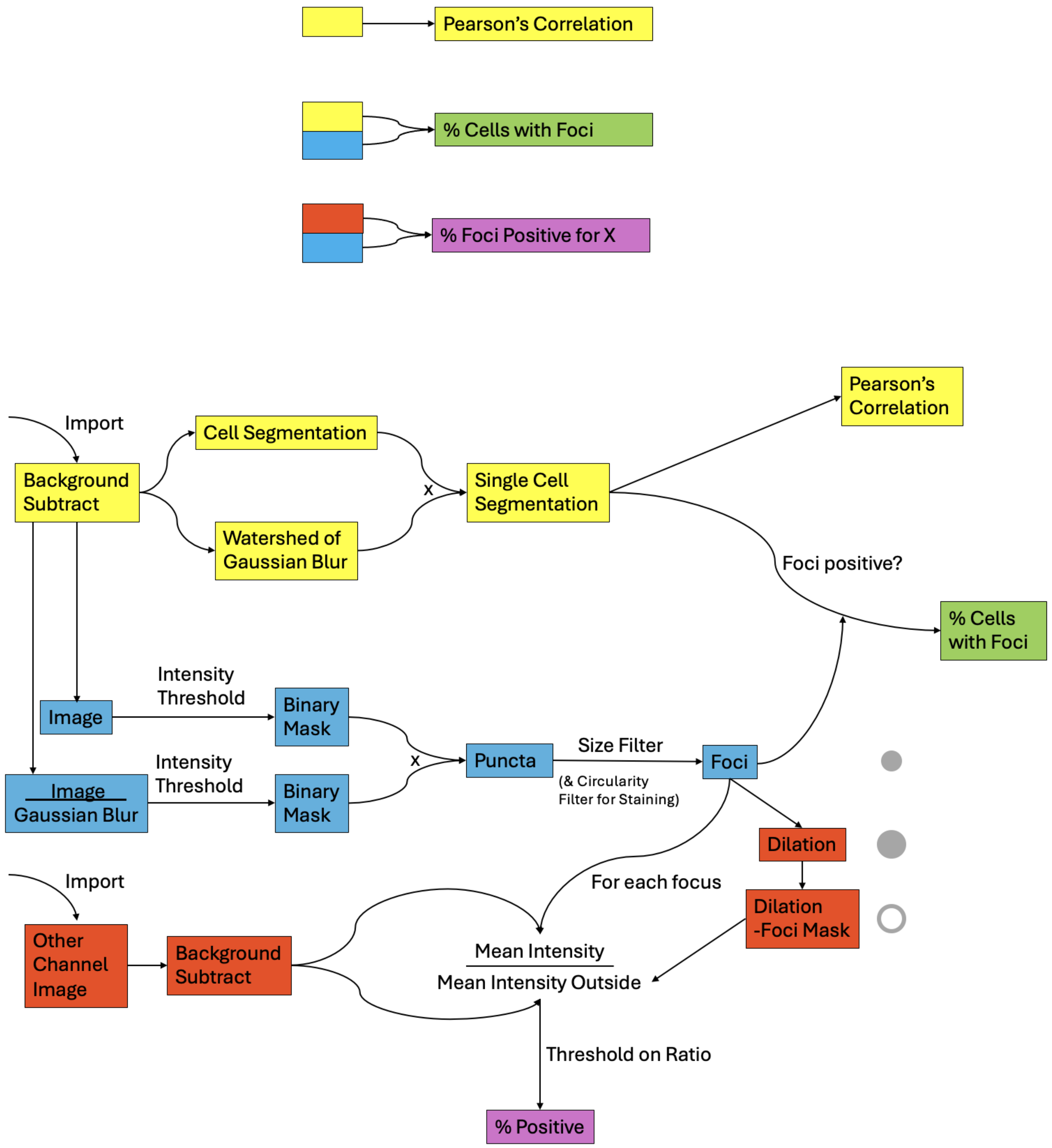

Appendix Figure S2.
Schematic of MATLAB workflow for image analysis.
