# Supplementary material for: STING induces HOIP-mediated synthesis of M1 ubiquitin chains to stimulate NF-κB signaling
Source: EMBO J. 2024 Nov 22;44(1):141–65. doi: 10.1038/s44318-024-00291-2 (PMC11696098; doi:10.1038/s44318-024-00291-2)
Supplement: Supplementary file 3 — Movie EV1 [file 44318_2024_291_MOESM3_ESM.zip › SD Movie EV1/EV-Movie-1_Legend.docx]

**Expanded View Movie 1.**

Representative spinning disk confocal movie of FRT/TREX HeLa cells stably expressing FRT/TO-DD-Vx3-EGFP, BFP-P2A-STING, and mScarletI-LC3B. Cells were incubated with 1 µg/mL Doxycycline and 500 nM Shield1 for 24h prior to treatment with 120 µg/mL cGAMP and imaging every 30 minutes for 12 hours on a spinning disk confocal microscope. Scale bar = 25 µm.
