# Supplementary figures and images for: STING induces HOIP-mediated synthesis of M1 ubiquitin chains to stimulate NF-κB signaling
Source: EMBO J. 2024 Nov 22;44(1):141–65. doi: 10.1038/s44318-024-00291-2 (PMC11696098; doi:10.1038/s44318-024-00291-2)

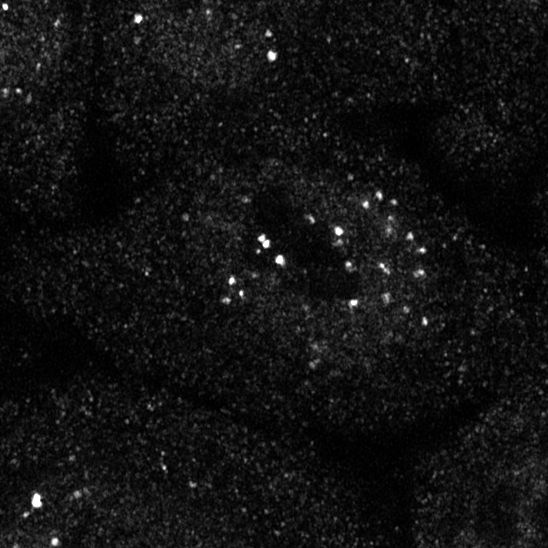

Supplement: Supplementary file 4 — Source data Fig. 1 [file 44318_2024_291_MOESM4_ESM.zip › SD Figure 1/EMBOJ-2023-115976_Fig1A-1_nt.tif]

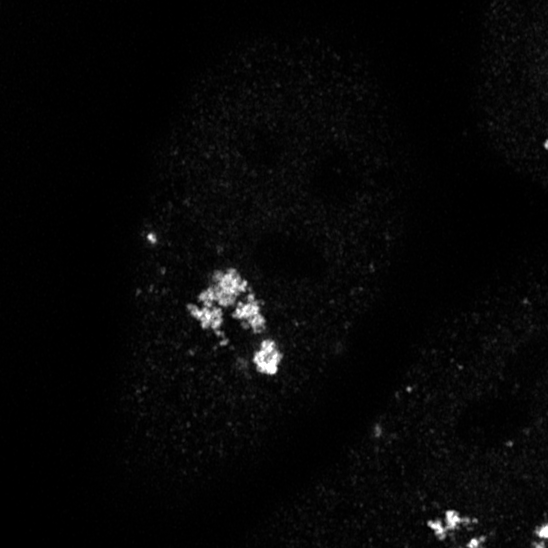

Supplement: Supplementary file 4 — Source data Fig. 1 [file 44318_2024_291_MOESM4_ESM.zip › SD Figure 1/EMBOJ-2023-115976_Fig1A-2_cGAMP.tif]

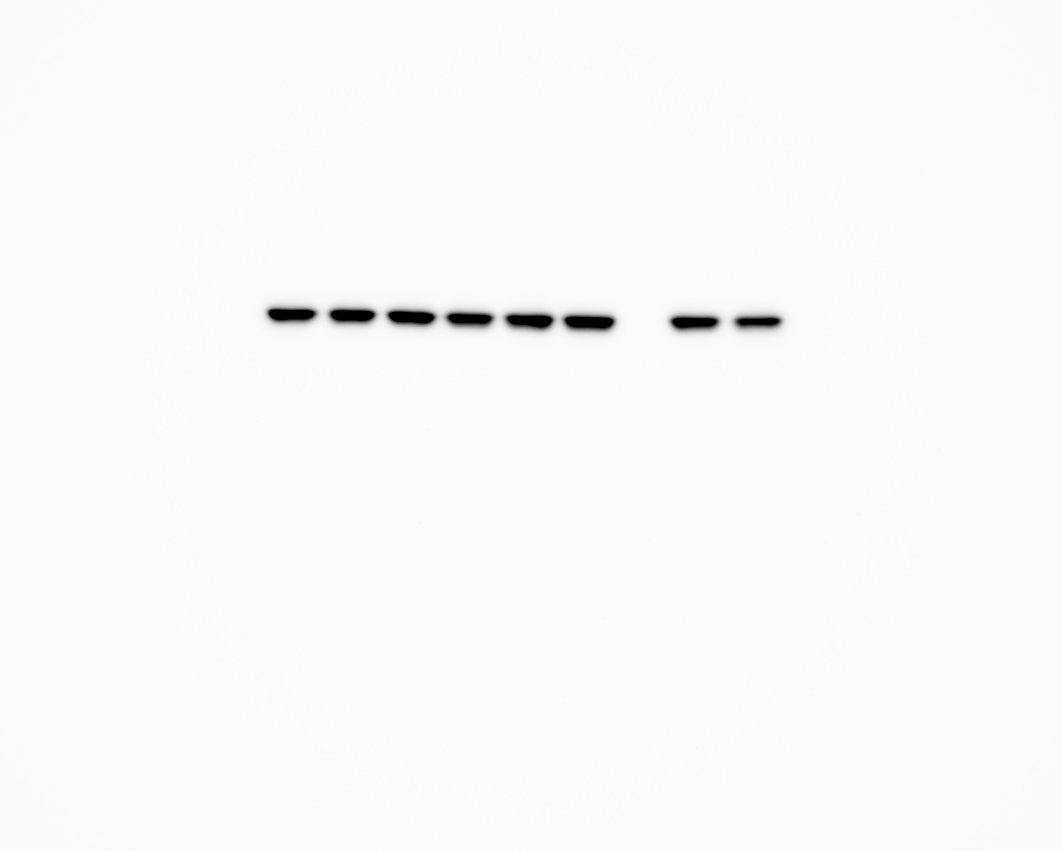

Supplement: Supplementary file 4 — Source data Fig. 1 [file 44318_2024_291_MOESM4_ESM.zip › SD Figure 1/EMBOJ-2023-115976_Fig1C-1_gapdh.tif]

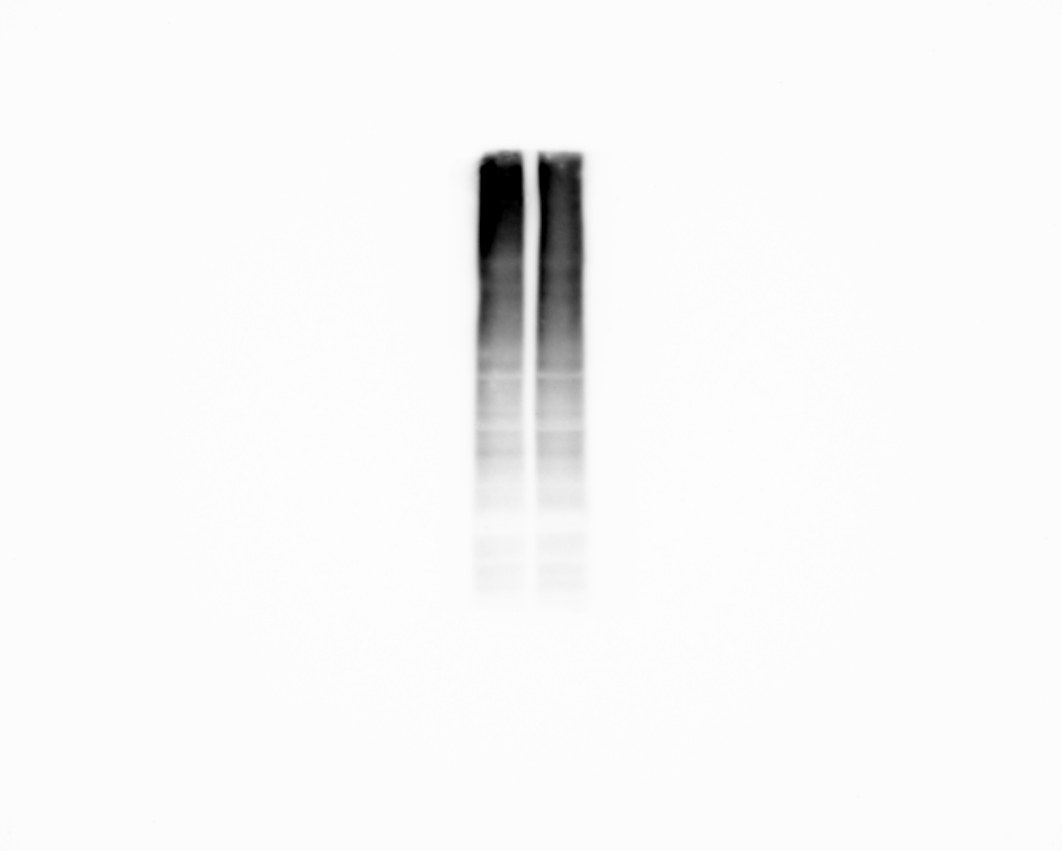

Supplement: Supplementary file 4 — Source data Fig. 1 [file 44318_2024_291_MOESM4_ESM.zip › SD Figure 1/EMBOJ-2023-115976_Fig1C-2_VU1.tif]

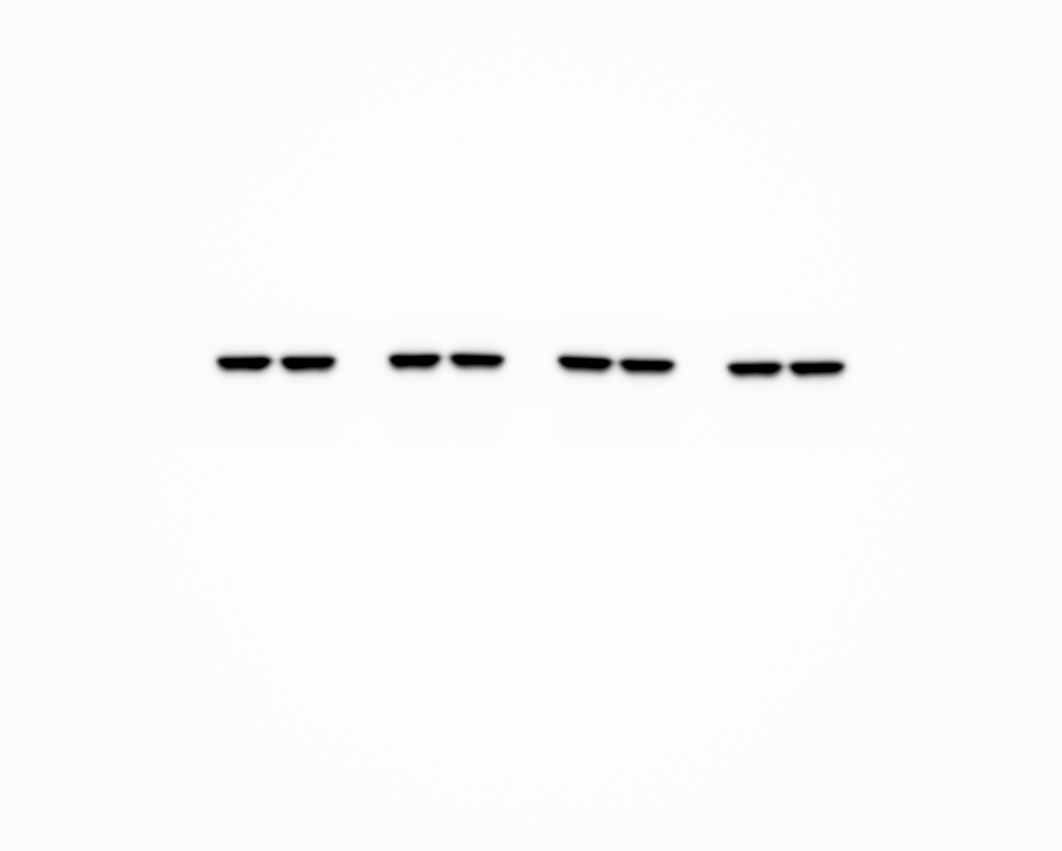

Supplement: Supplementary file 4 — Source data Fig. 1 [file 44318_2024_291_MOESM4_ESM.zip › SD Figure 1/EMBOJ-2023-115976_Fig1D-1_gapdh.tif]

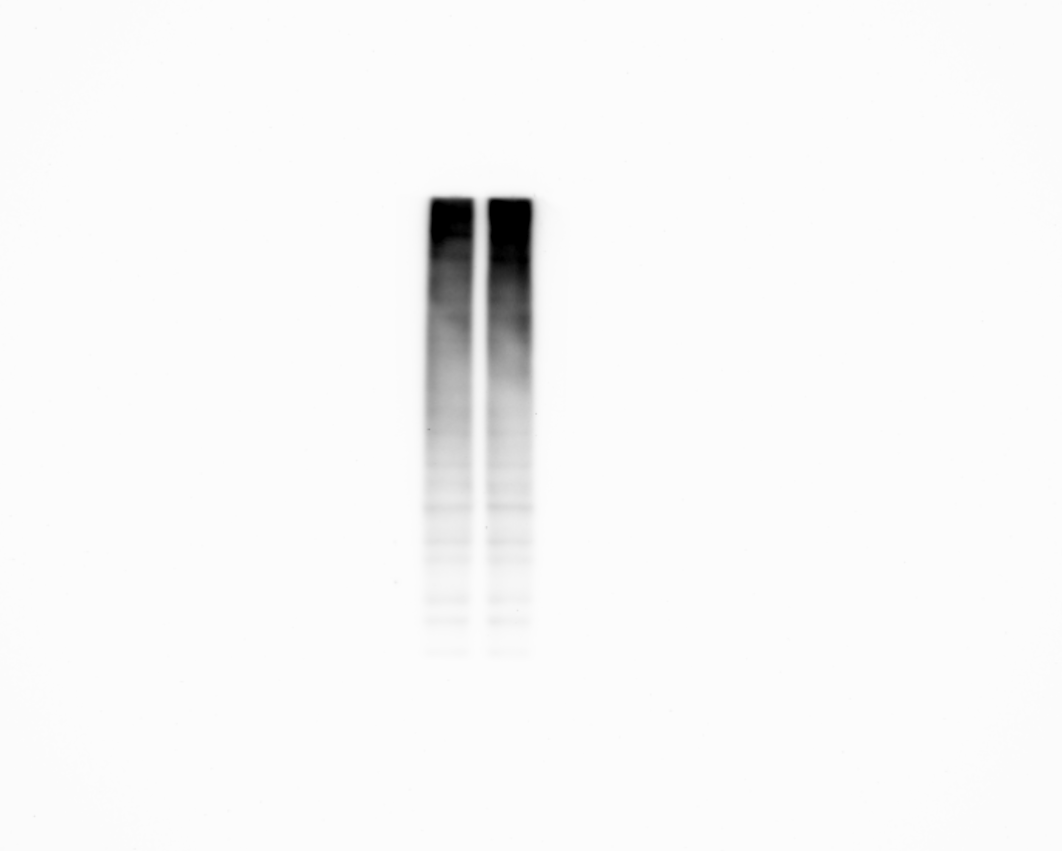

Supplement: Supplementary file 4 — Source data Fig. 1 [file 44318_2024_291_MOESM4_ESM.zip › SD Figure 1/EMBOJ-2023-115976_Fig1D-2_K48.tif]

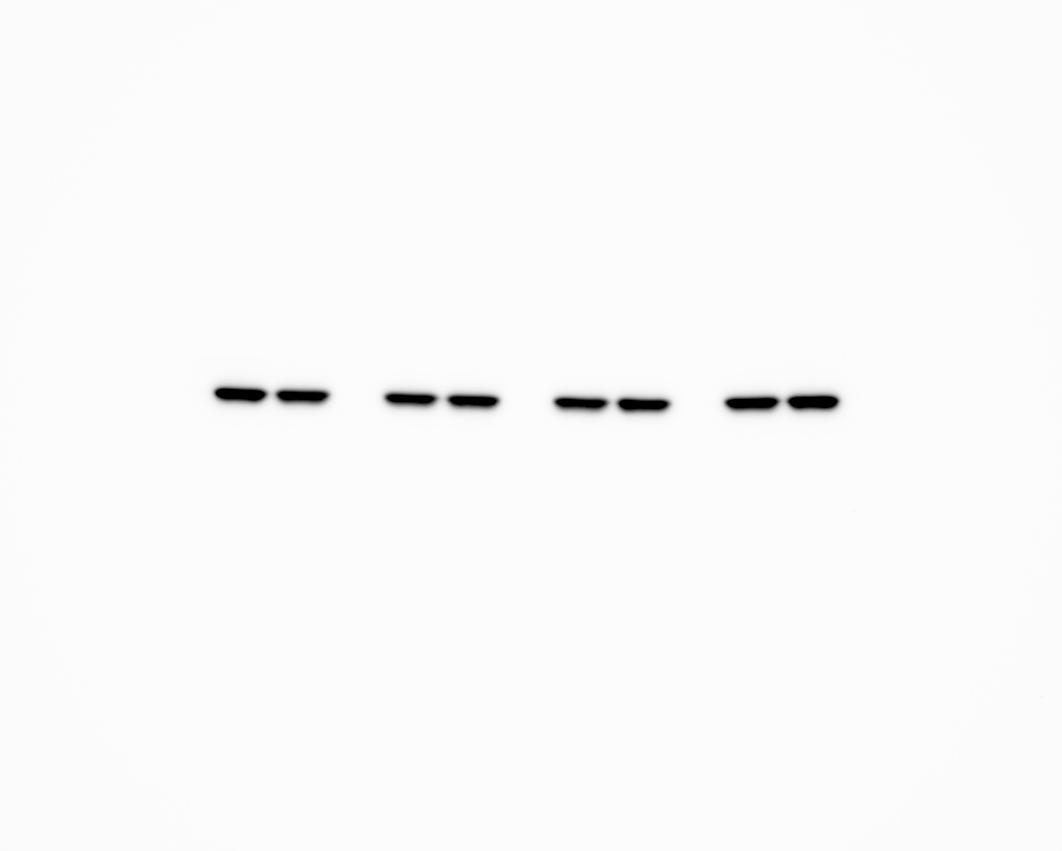

Supplement: Supplementary file 4 — Source data Fig. 1 [file 44318_2024_291_MOESM4_ESM.zip › SD Figure 1/EMBOJ-2023-115976_Fig1E-1_gapdh.tif]

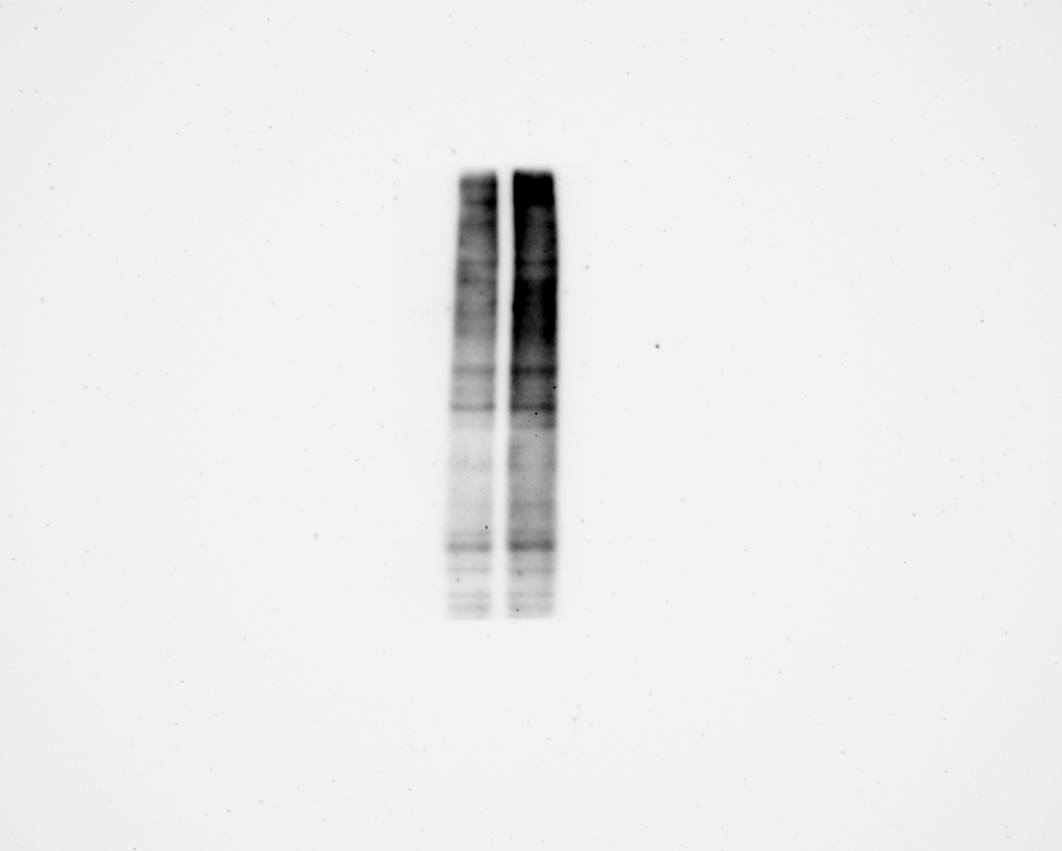

Supplement: Supplementary file 4 — Source data Fig. 1 [file 44318_2024_291_MOESM4_ESM.zip › SD Figure 1/EMBOJ-2023-115976_Fig1E-2_K63.tif]

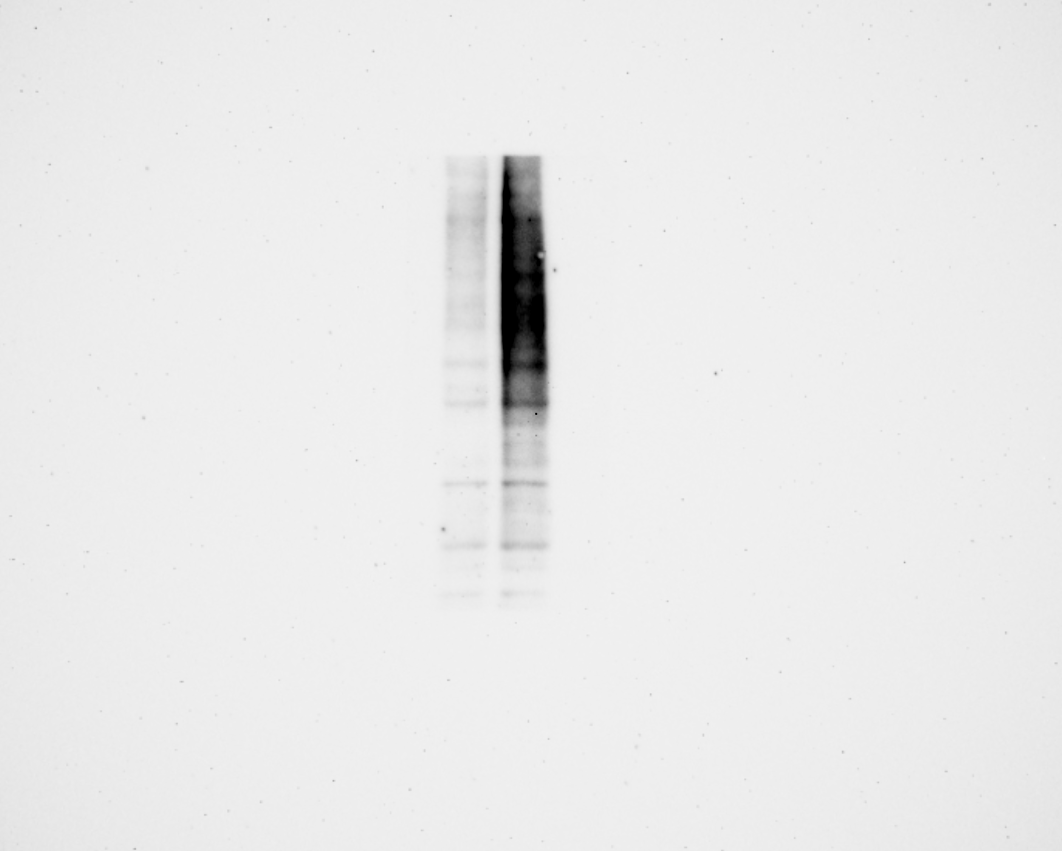

Supplement: Supplementary file 4 — Source data Fig. 1 [file 44318_2024_291_MOESM4_ESM.zip › SD Figure 1/EMBOJ-2023-115976_Fig1F-2_M1.tif]

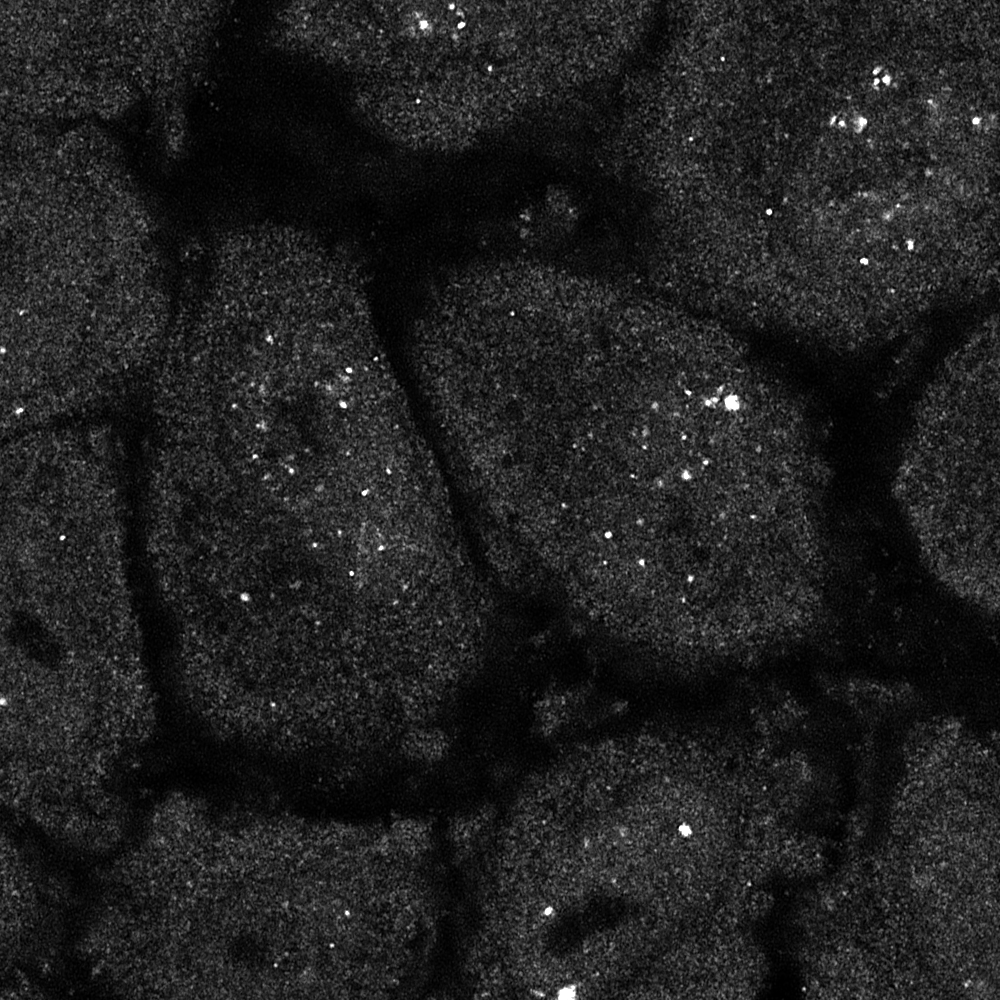

Supplement: Supplementary file 4 — Source data Fig. 1 [file 44318_2024_291_MOESM4_ESM.zip › SD Figure 1/EMBOJ-2023-115976_Fig1I-1_nt.tif]

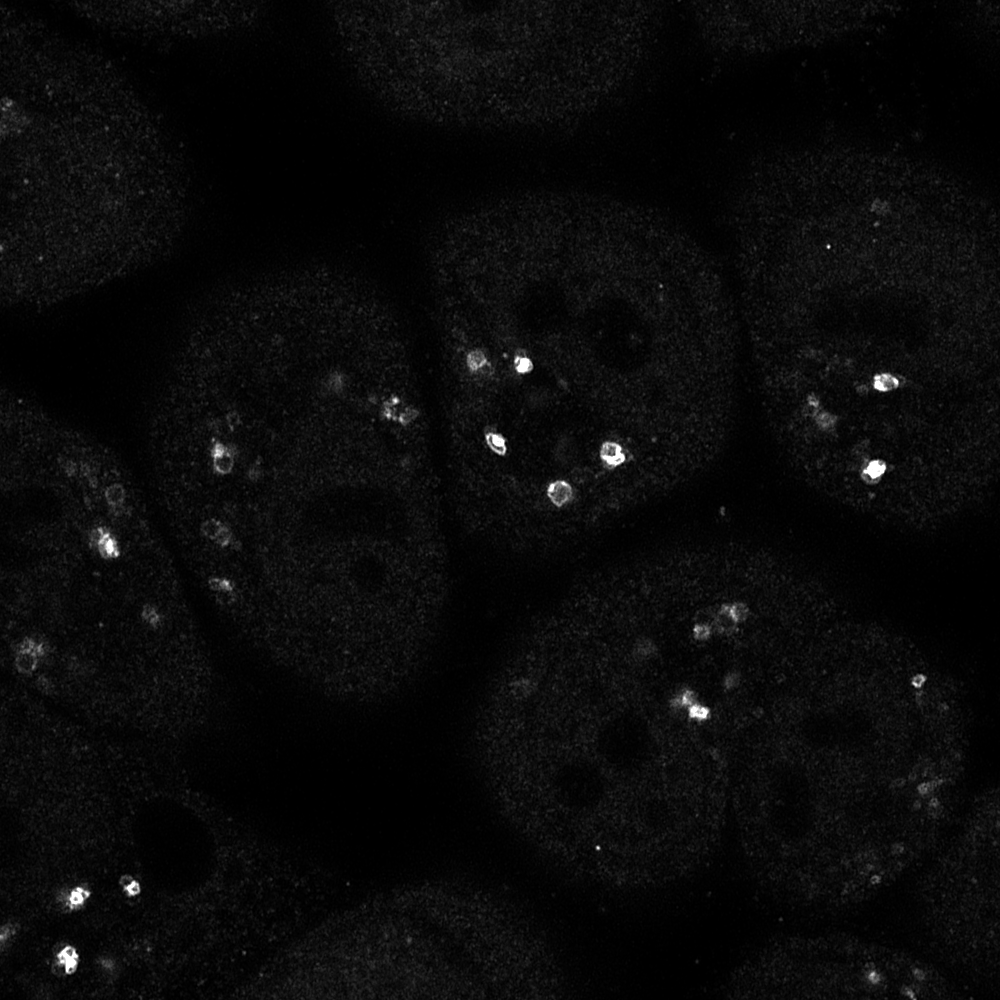

Supplement: Supplementary file 4 — Source data Fig. 1 [file 44318_2024_291_MOESM4_ESM.zip › SD Figure 1/EMBOJ-2023-115976_Fig1I-2_Mon.tif]

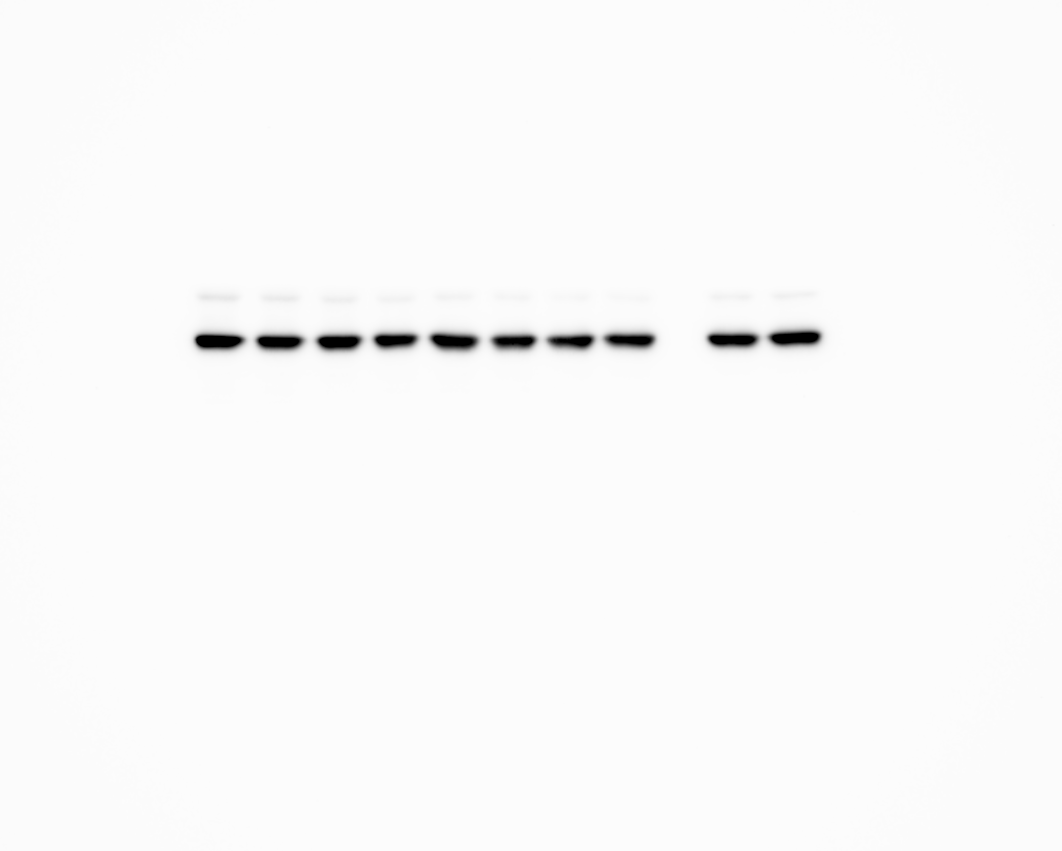

Supplement: Supplementary file 4 — Source data Fig. 1 [file 44318_2024_291_MOESM4_ESM.zip › SD Figure 1/EMBOJ-2023-115976_Fig1J-1_gapdh.tif]

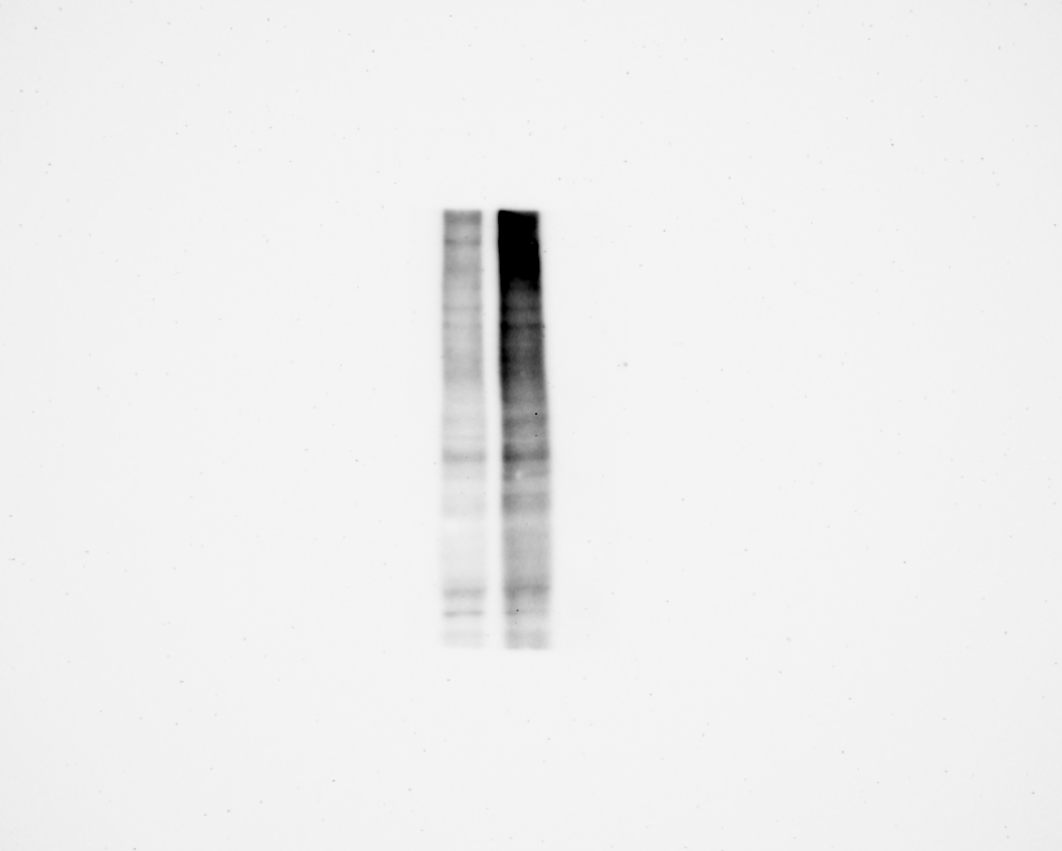

Supplement: Supplementary file 4 — Source data Fig. 1 [file 44318_2024_291_MOESM4_ESM.zip › SD Figure 1/EMBOJ-2023-115976_Fig1J-2_K63.tif]

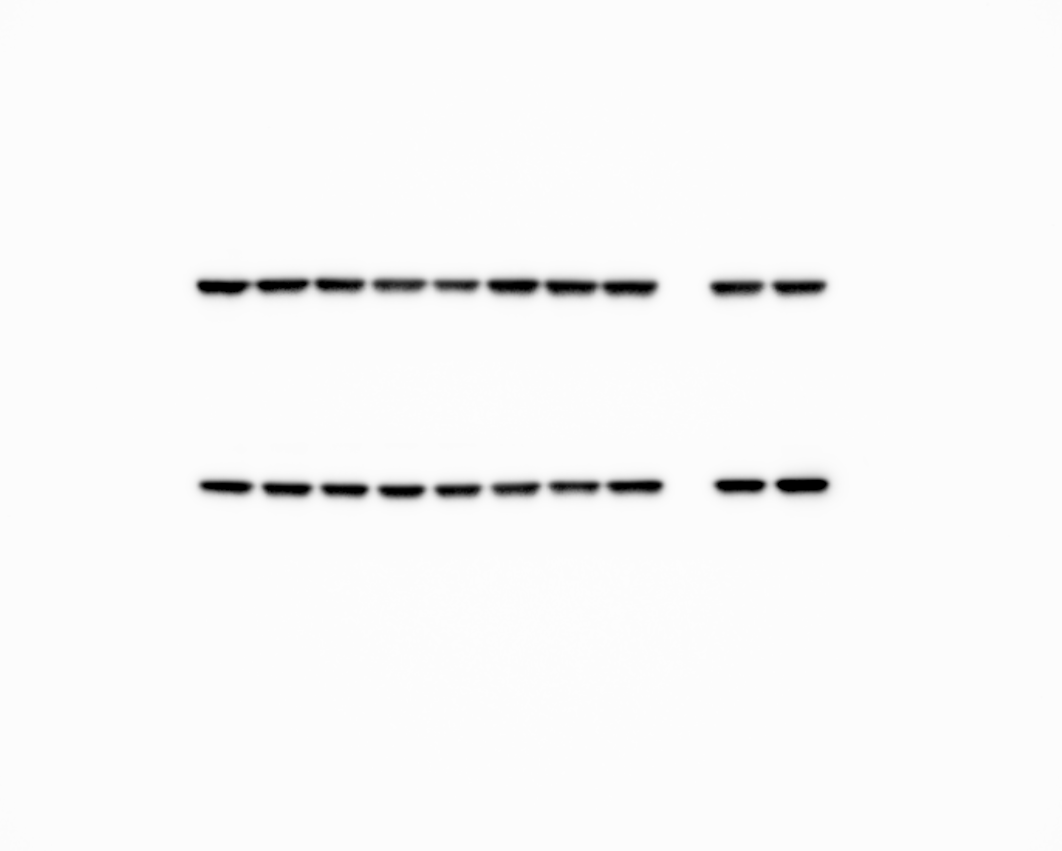

Supplement: Supplementary file 4 — Source data Fig. 1 [file 44318_2024_291_MOESM4_ESM.zip › SD Figure 1/EMBOJ-2023-115976_Fig1K-1_gapdh.tif]

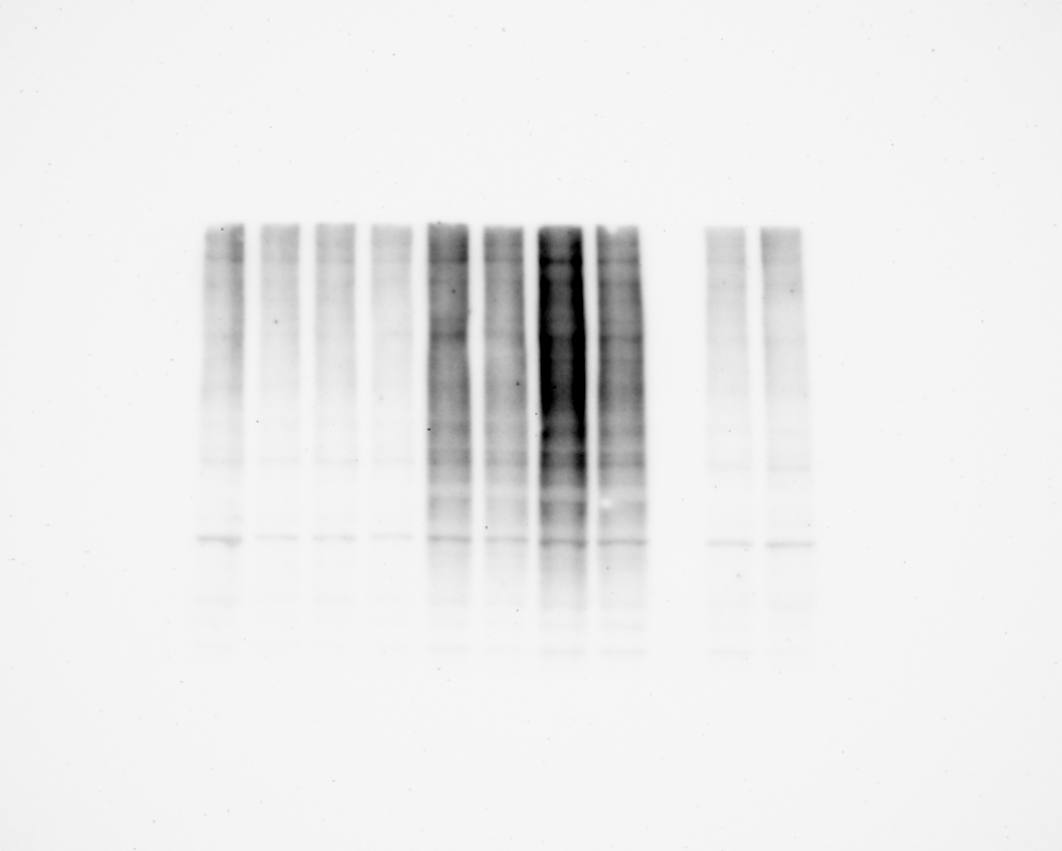

Supplement: Supplementary file 4 — Source data Fig. 1 [file 44318_2024_291_MOESM4_ESM.zip › SD Figure 1/EMBOJ-2023-115976_Fig1K-2_M1.tif]

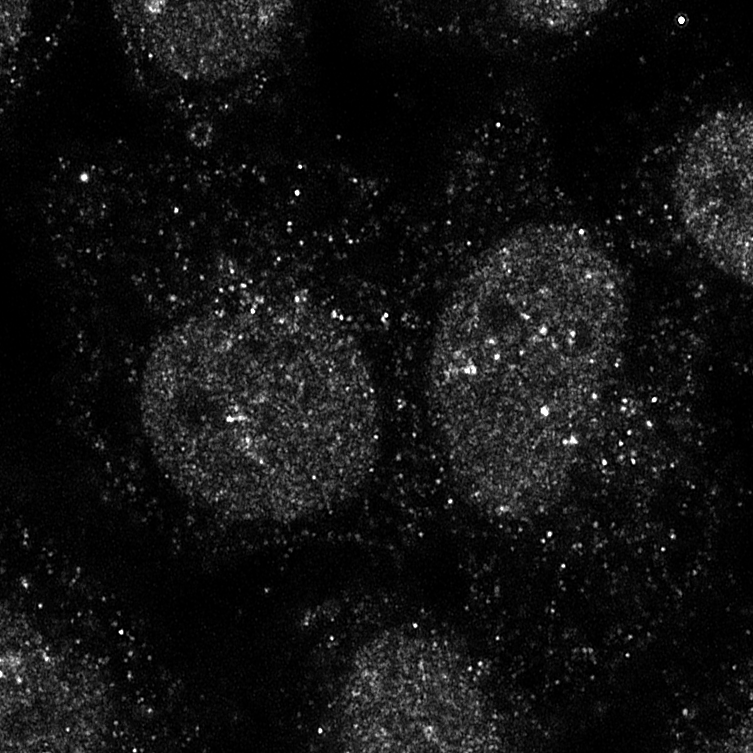

Supplement: Supplementary file 5 — Source data Fig. 2 [file 44318_2024_291_MOESM5_ESM.zip › SD Figure 2/EMBOJ-2023-115976_Fig2A-1_nt.tif]

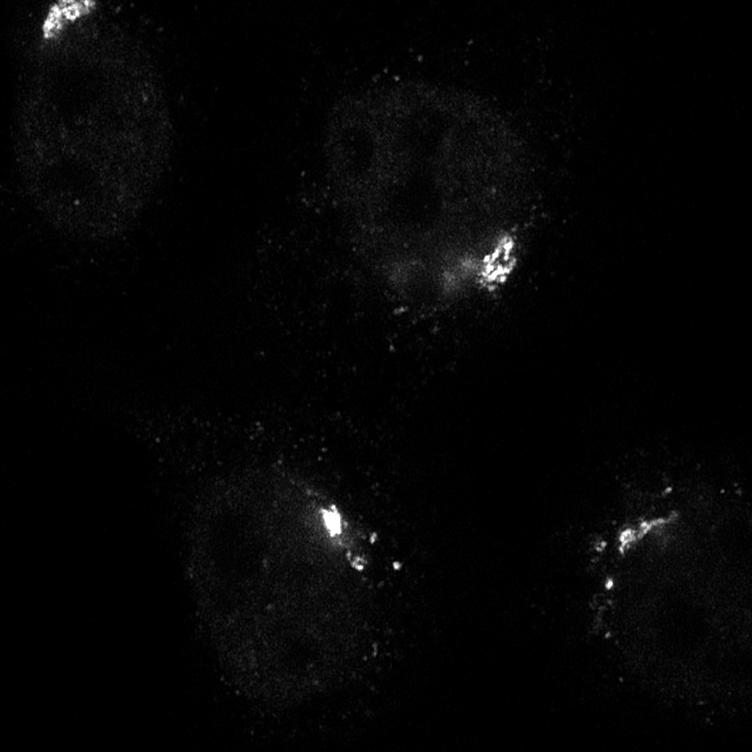

Supplement: Supplementary file 5 — Source data Fig. 2 [file 44318_2024_291_MOESM5_ESM.zip › SD Figure 2/EMBOJ-2023-115976_Fig2A-2_cGAMP.tif]

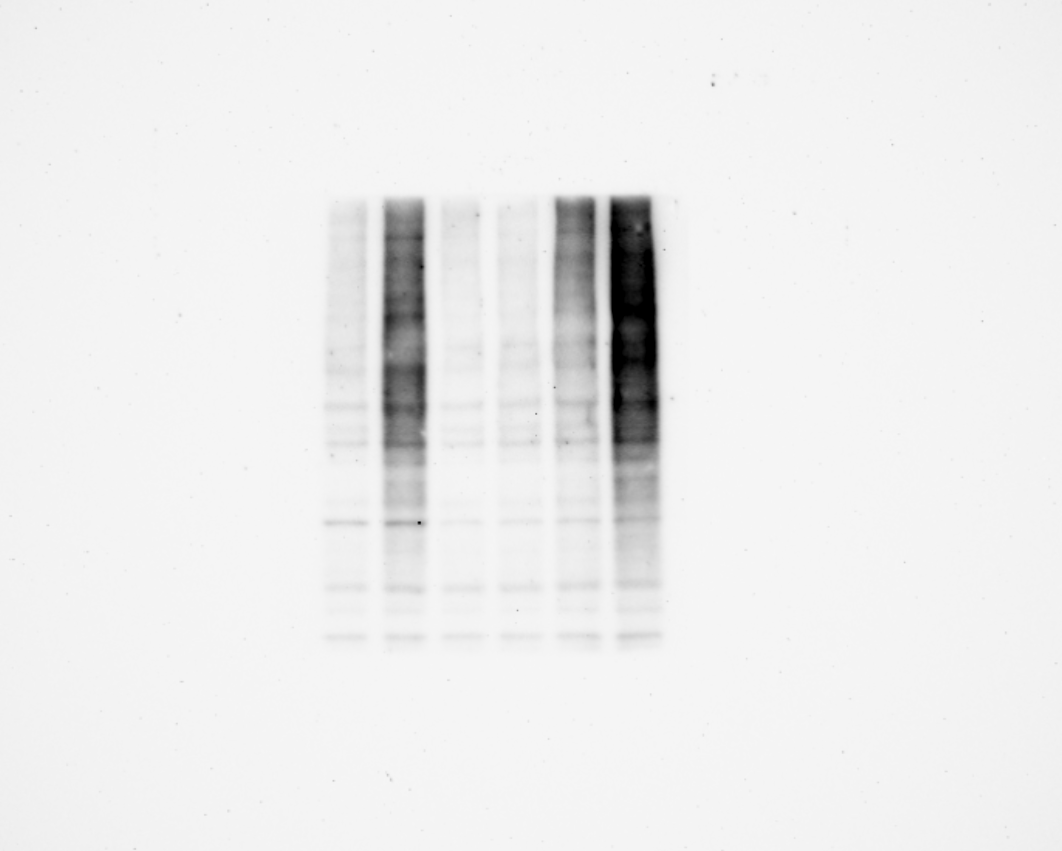

Supplement: Supplementary file 5 — Source data Fig. 2 [file 44318_2024_291_MOESM5_ESM.zip › SD Figure 2/EMBOJ-2023-115976_Fig2C-1_M1.tif]

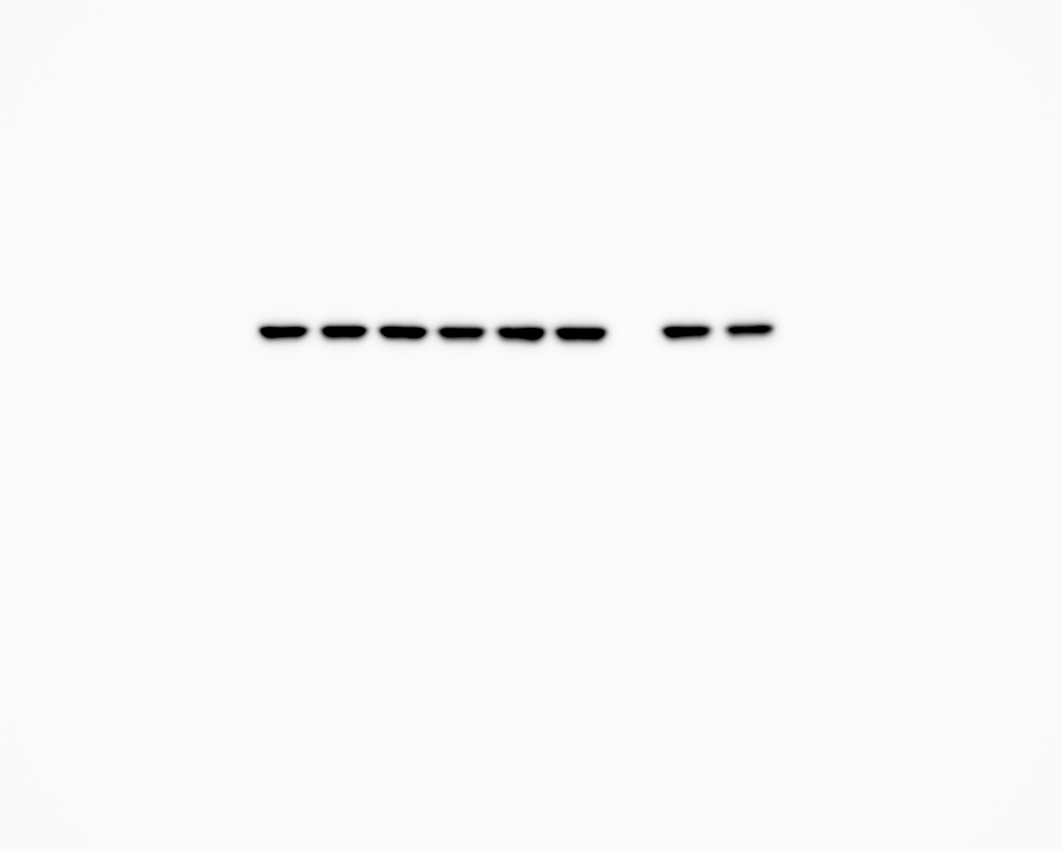

Supplement: Supplementary file 5 — Source data Fig. 2 [file 44318_2024_291_MOESM5_ESM.zip › SD Figure 2/EMBOJ-2023-115976_Fig2C-2_gapdh.tif]

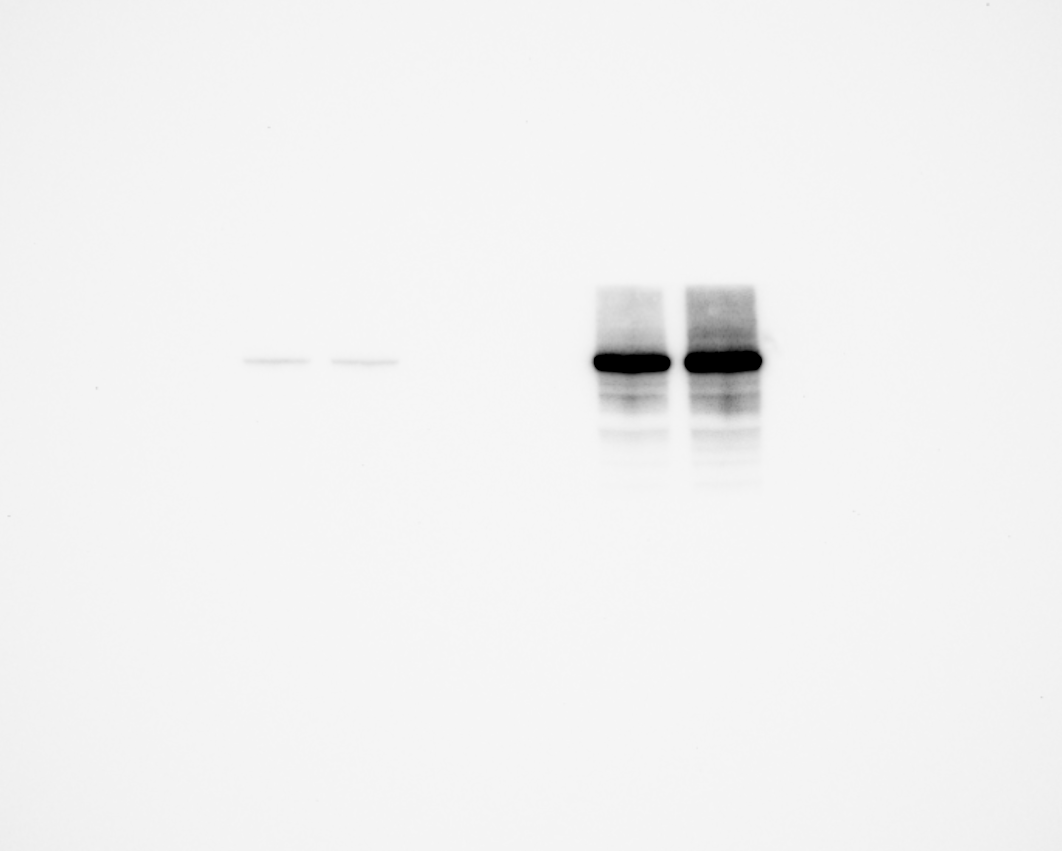

Supplement: Supplementary file 5 — Source data Fig. 2 [file 44318_2024_291_MOESM5_ESM.zip › SD Figure 2/EMBOJ-2023-115976_Fig2C-3_HOIP.tif]

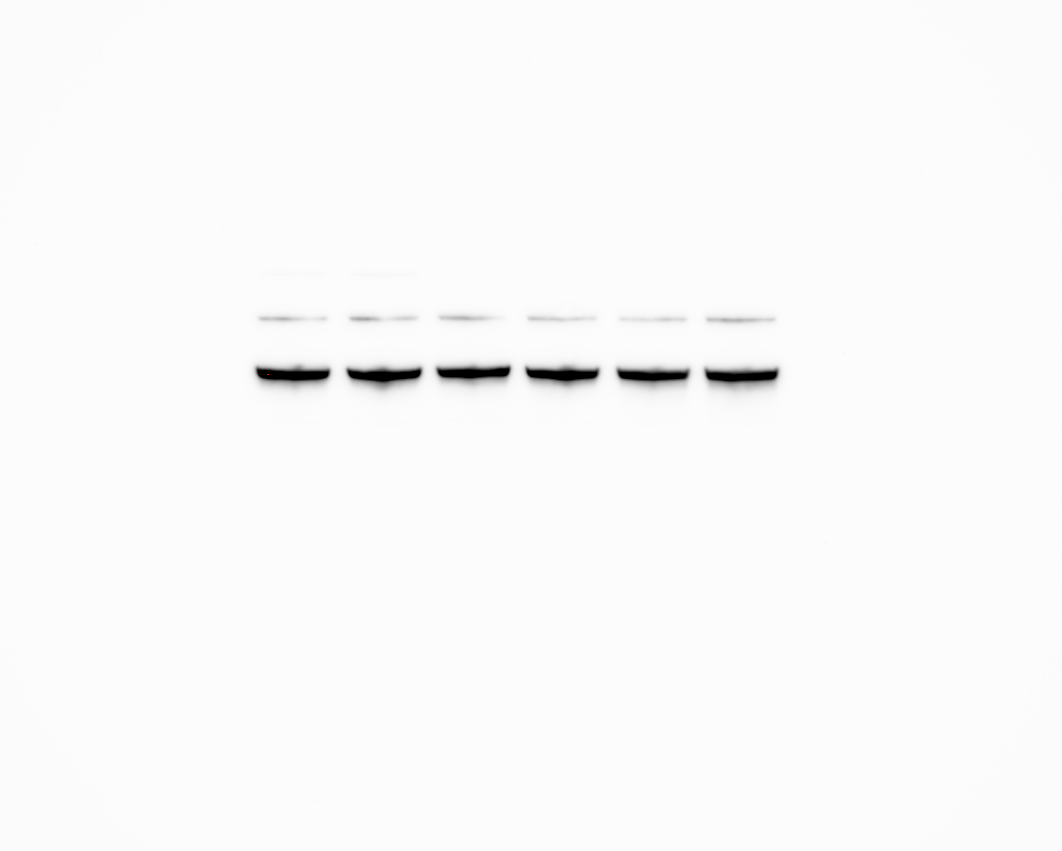

Supplement: Supplementary file 5 — Source data Fig. 2 [file 44318_2024_291_MOESM5_ESM.zip › SD Figure 2/EMBOJ-2023-115976_Fig2C-4_HSP90.tif]

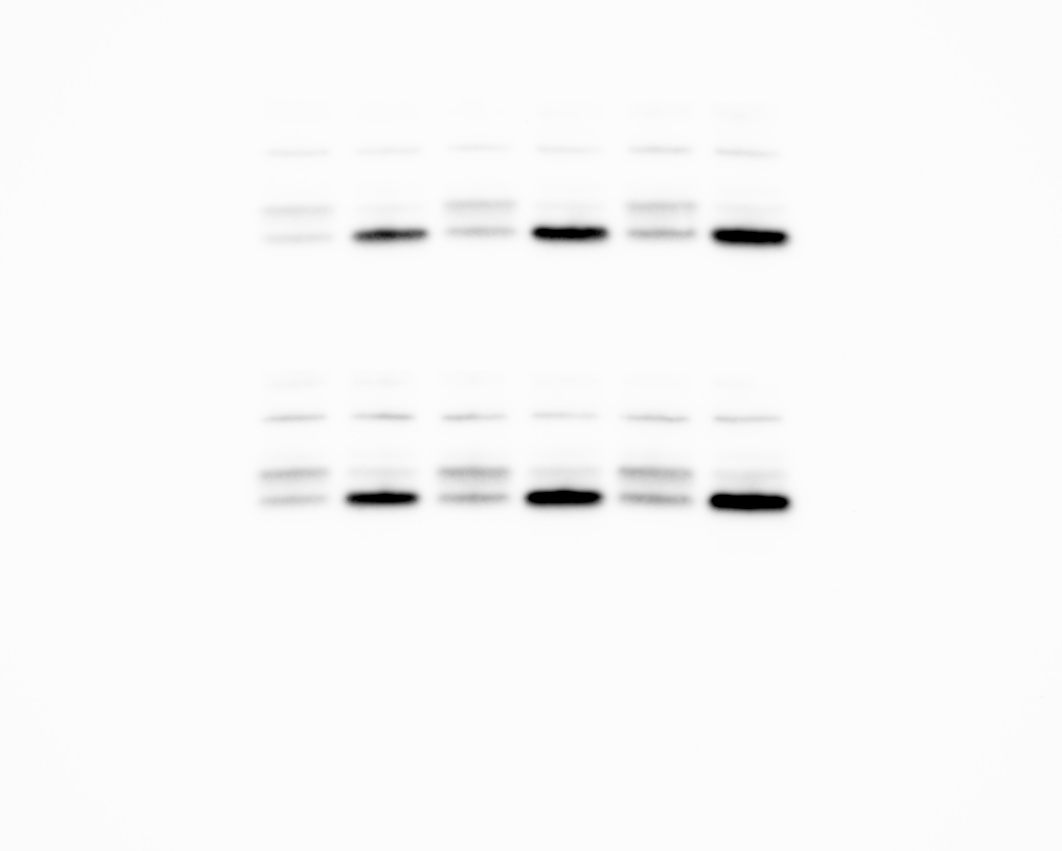

Supplement: Supplementary file 5 — Source data Fig. 2 [file 44318_2024_291_MOESM5_ESM.zip › SD Figure 2/EMBOJ-2023-115976_Fig2C-5_LC3B.tif]

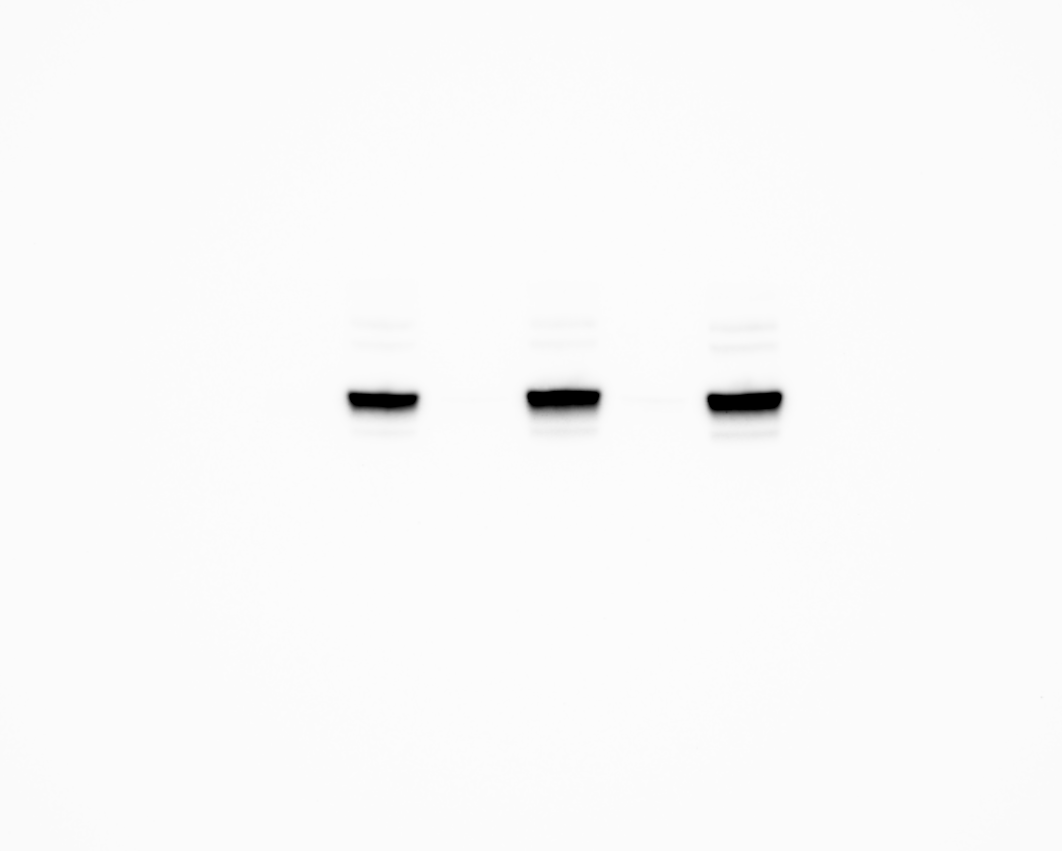

Supplement: Supplementary file 5 — Source data Fig. 2 [file 44318_2024_291_MOESM5_ESM.zip › SD Figure 2/EMBOJ-2023-115976_Fig2C-6_pSTING.tif]

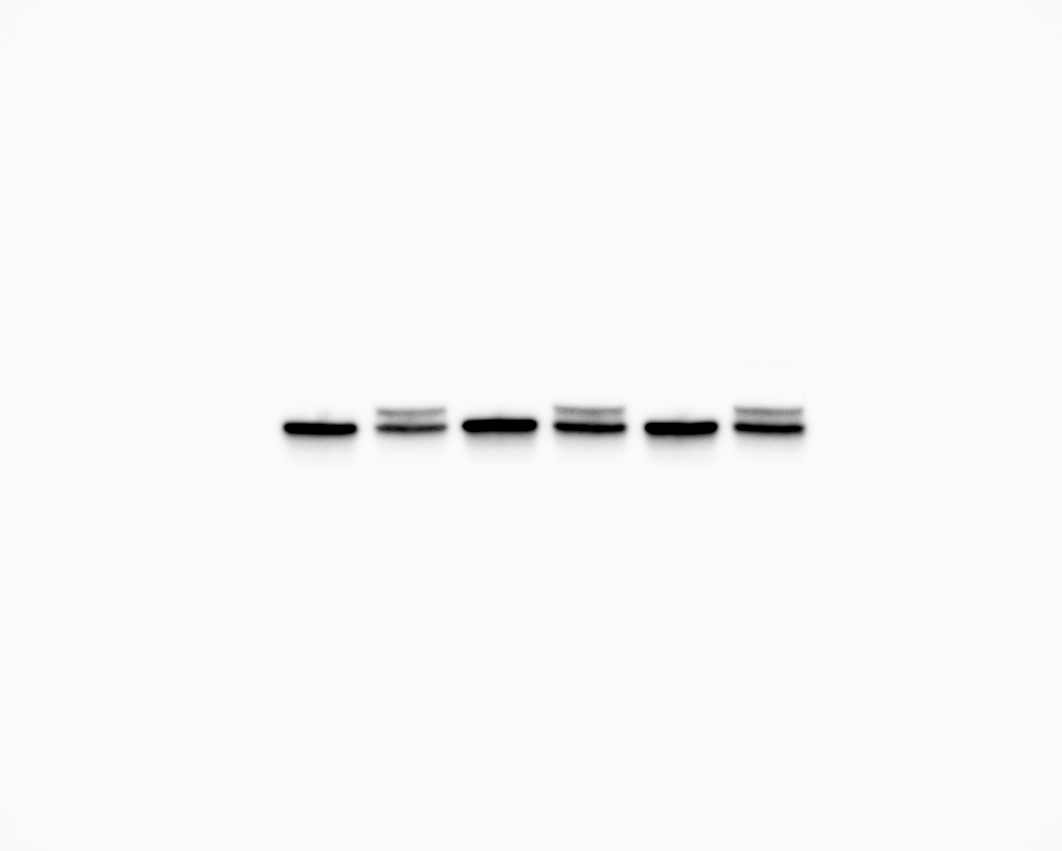

Supplement: Supplementary file 5 — Source data Fig. 2 [file 44318_2024_291_MOESM5_ESM.zip › SD Figure 2/EMBOJ-2023-115976_Fig2C-7_STING.tif]

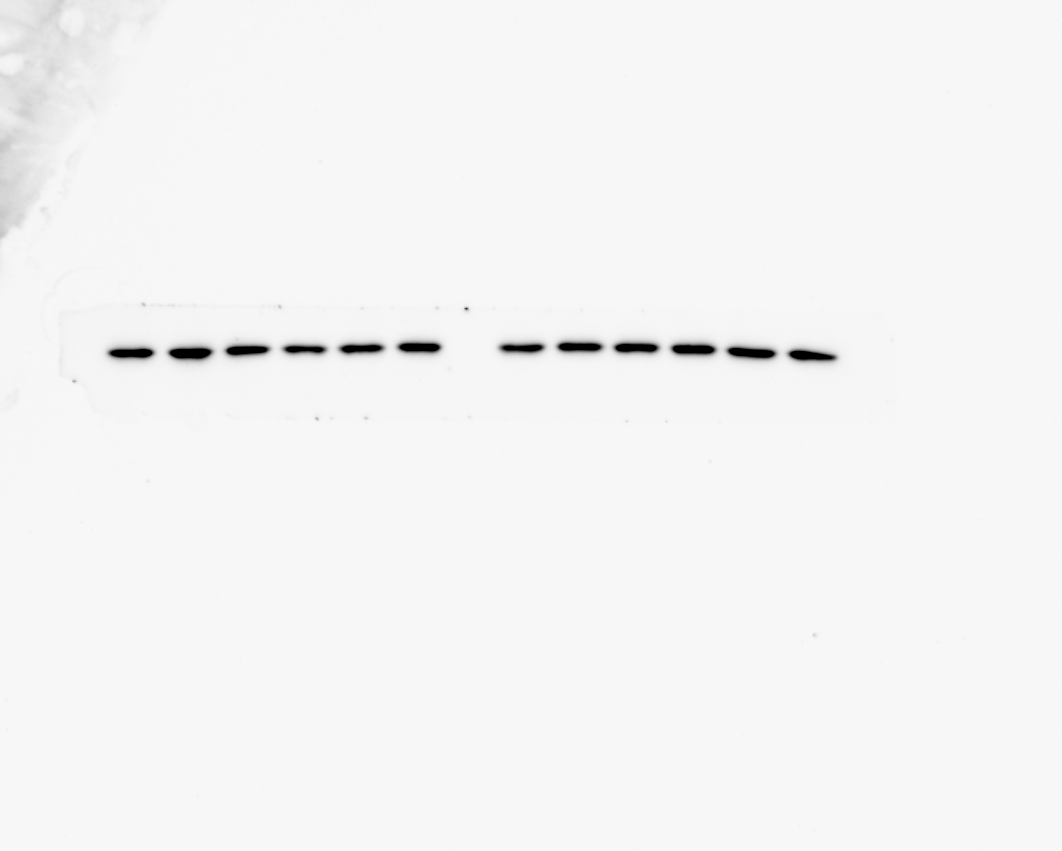

Supplement: Supplementary file 5 — Source data Fig. 2 [file 44318_2024_291_MOESM5_ESM.zip › SD Figure 2/EMBOJ-2023-115976_Fig2E-1_gapdh.tif]

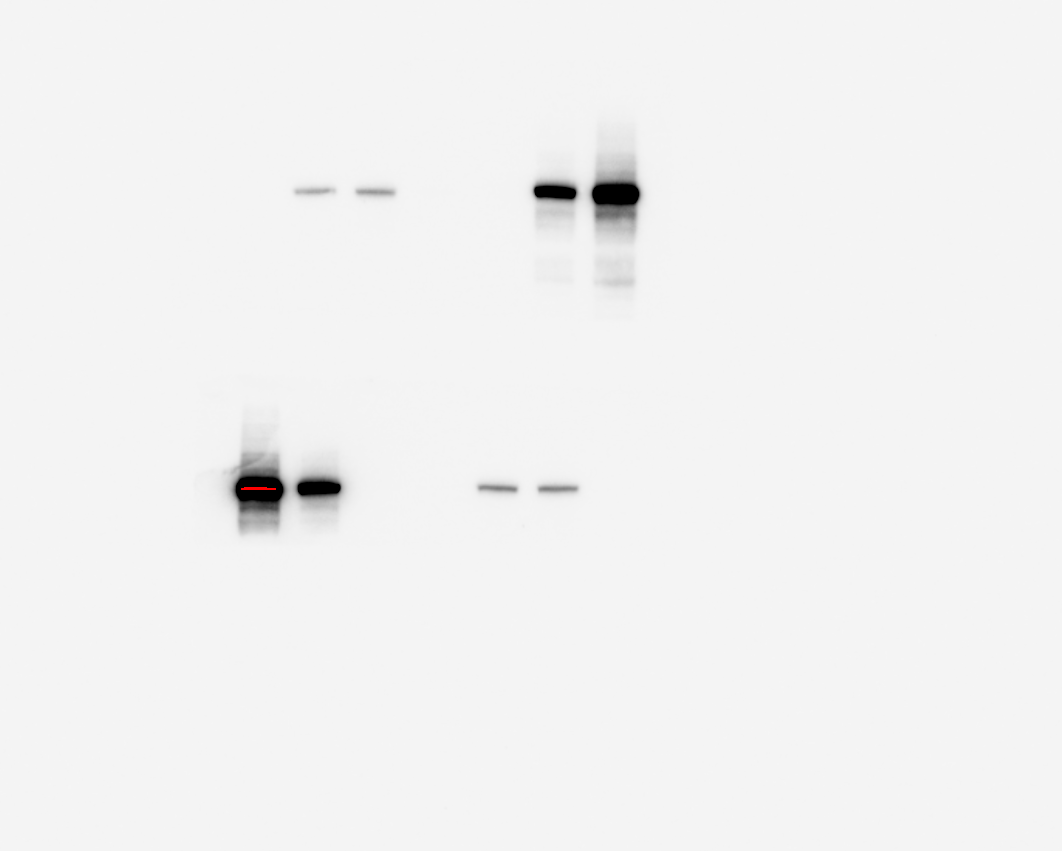

Supplement: Supplementary file 5 — Source data Fig. 2 [file 44318_2024_291_MOESM5_ESM.zip › SD Figure 2/EMBOJ-2023-115976_Fig2E-2_HOIP.tif]

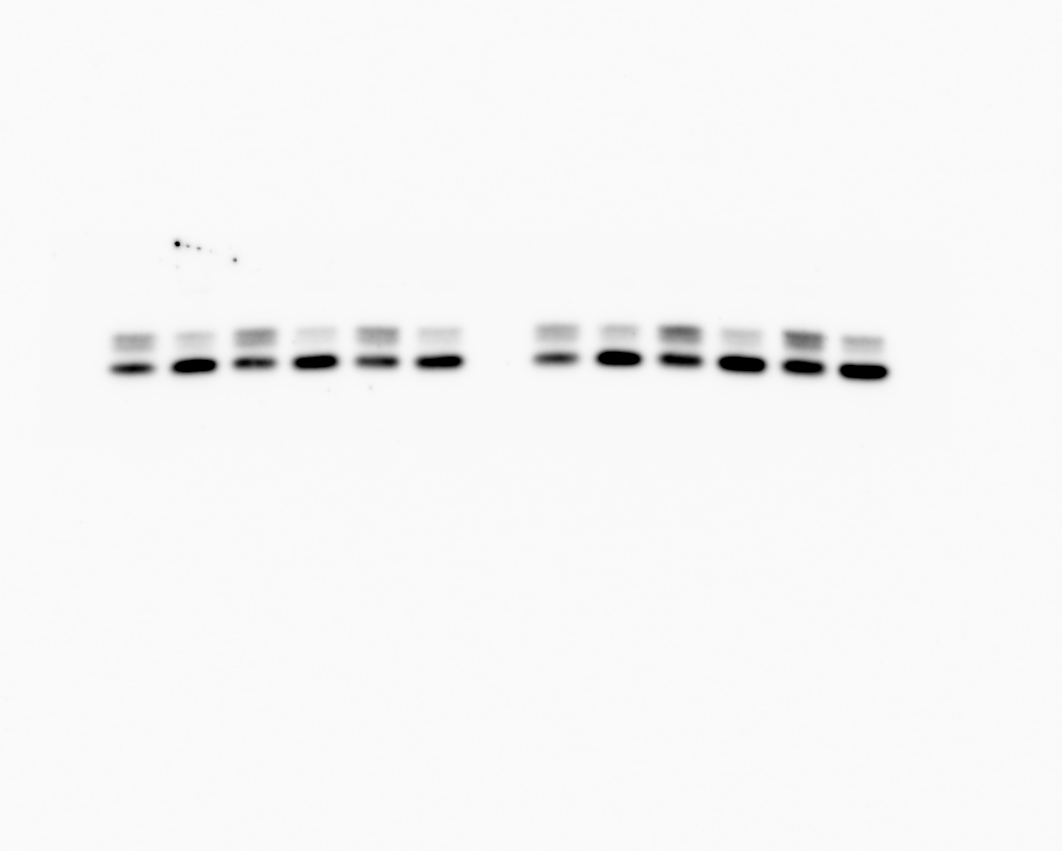

Supplement: Supplementary file 5 — Source data Fig. 2 [file 44318_2024_291_MOESM5_ESM.zip › SD Figure 2/EMBOJ-2023-115976_Fig2E-3_LC3B.tif]

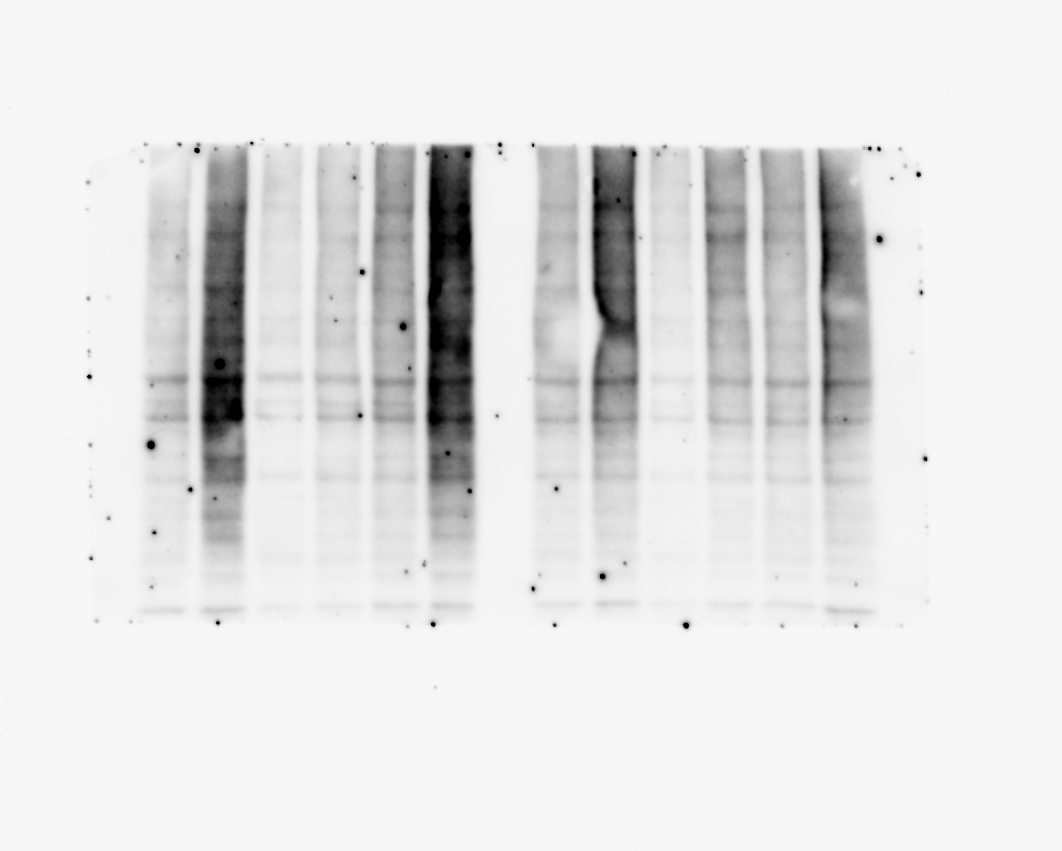

Supplement: Supplementary file 5 — Source data Fig. 2 [file 44318_2024_291_MOESM5_ESM.zip › SD Figure 2/EMBOJ-2023-115976_Fig2E-4_M1.tif]

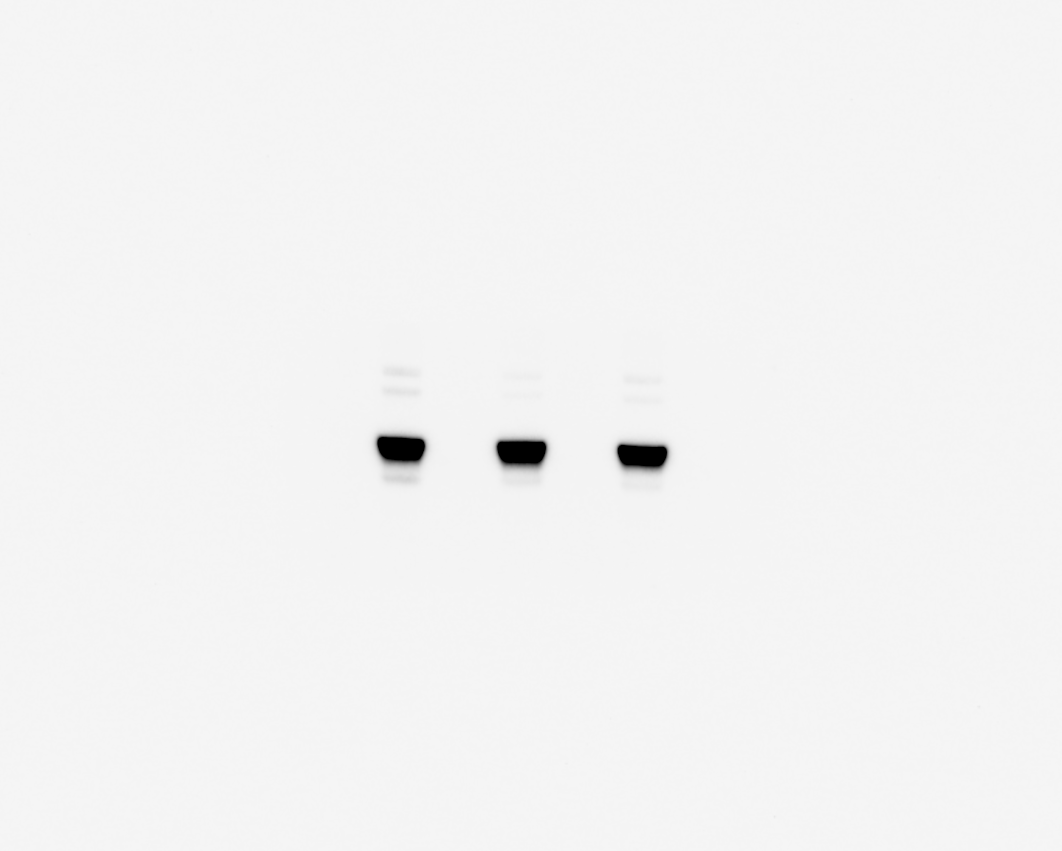

Supplement: Supplementary file 5 — Source data Fig. 2 [file 44318_2024_291_MOESM5_ESM.zip › SD Figure 2/EMBOJ-2023-115976_Fig2E-5_pSTING.tif]

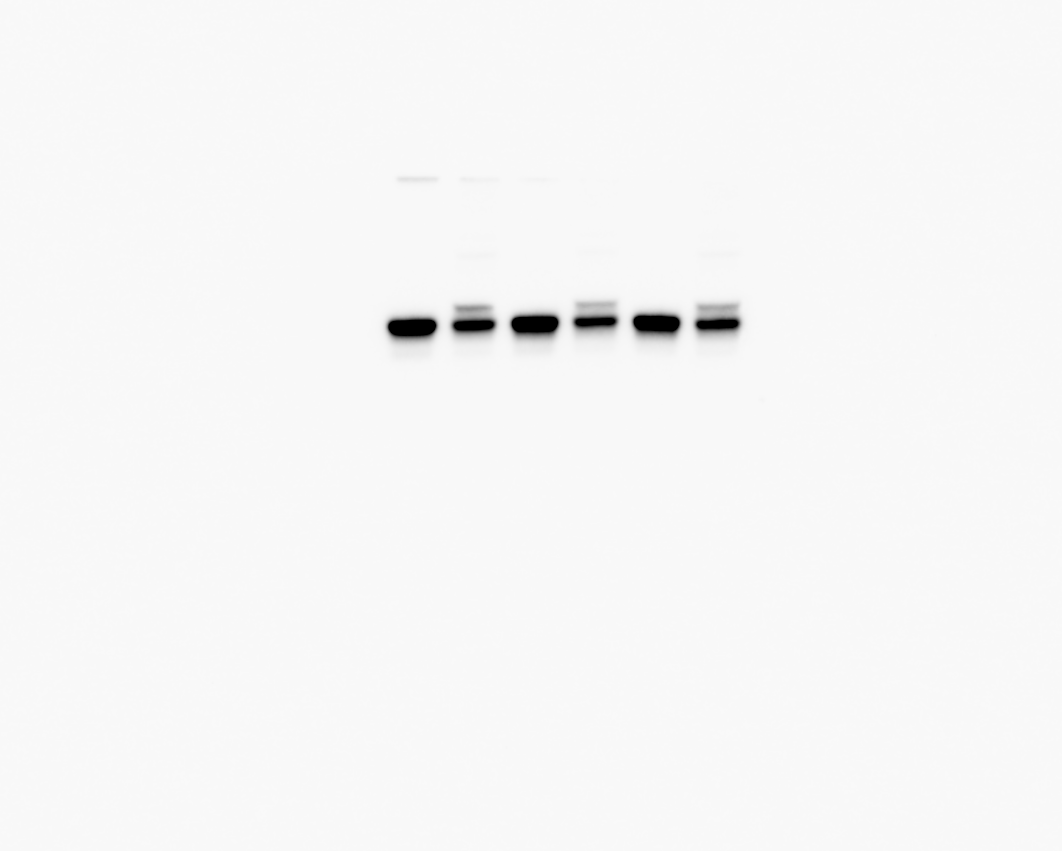

Supplement: Supplementary file 5 — Source data Fig. 2 [file 44318_2024_291_MOESM5_ESM.zip › SD Figure 2/EMBOJ-2023-115976_Fig2E-6_STING.tif]

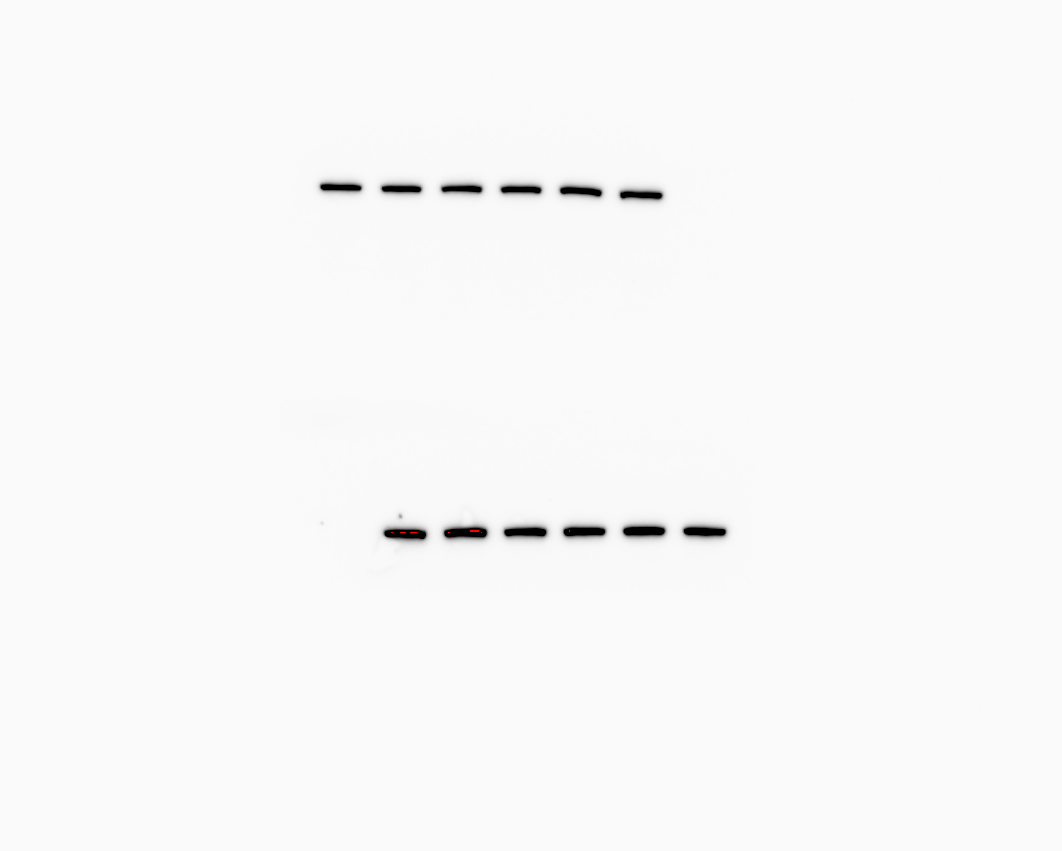

Supplement: Supplementary file 5 — Source data Fig. 2 [file 44318_2024_291_MOESM5_ESM.zip › SD Figure 2/EMBOJ-2023-115976_Fig2E-7_Vinc.tif]

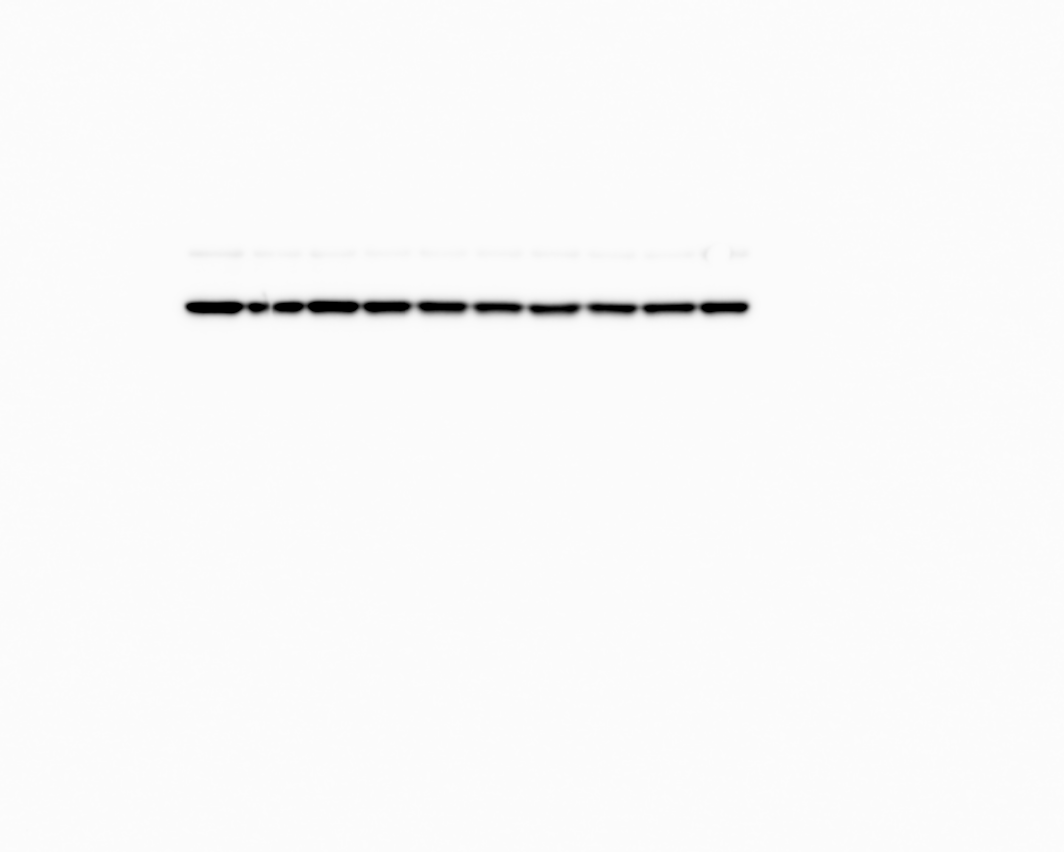

Supplement: Supplementary file 5 — Source data Fig. 2 [file 44318_2024_291_MOESM5_ESM.zip › SD Figure 2/EMBOJ-2023-115976_Fig2G-1_gapdh.tif]

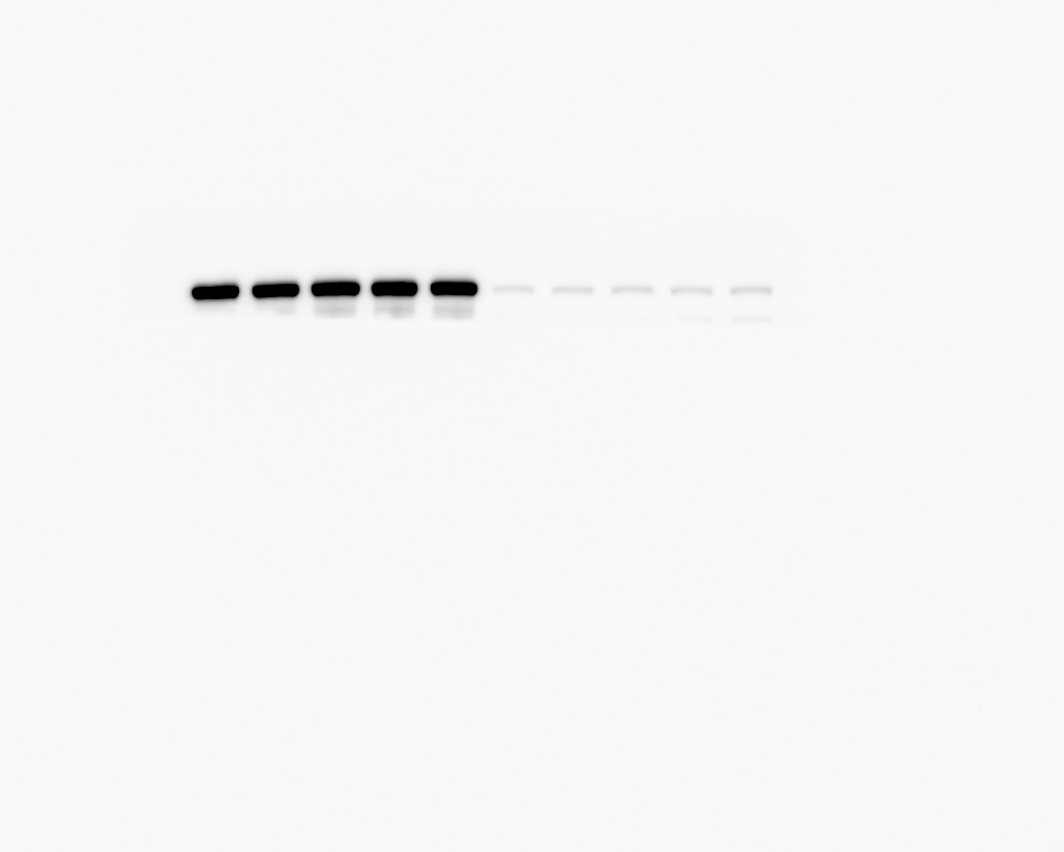

Supplement: Supplementary file 5 — Source data Fig. 2 [file 44318_2024_291_MOESM5_ESM.zip › SD Figure 2/EMBOJ-2023-115976_Fig2G-2_HOIP.tif]

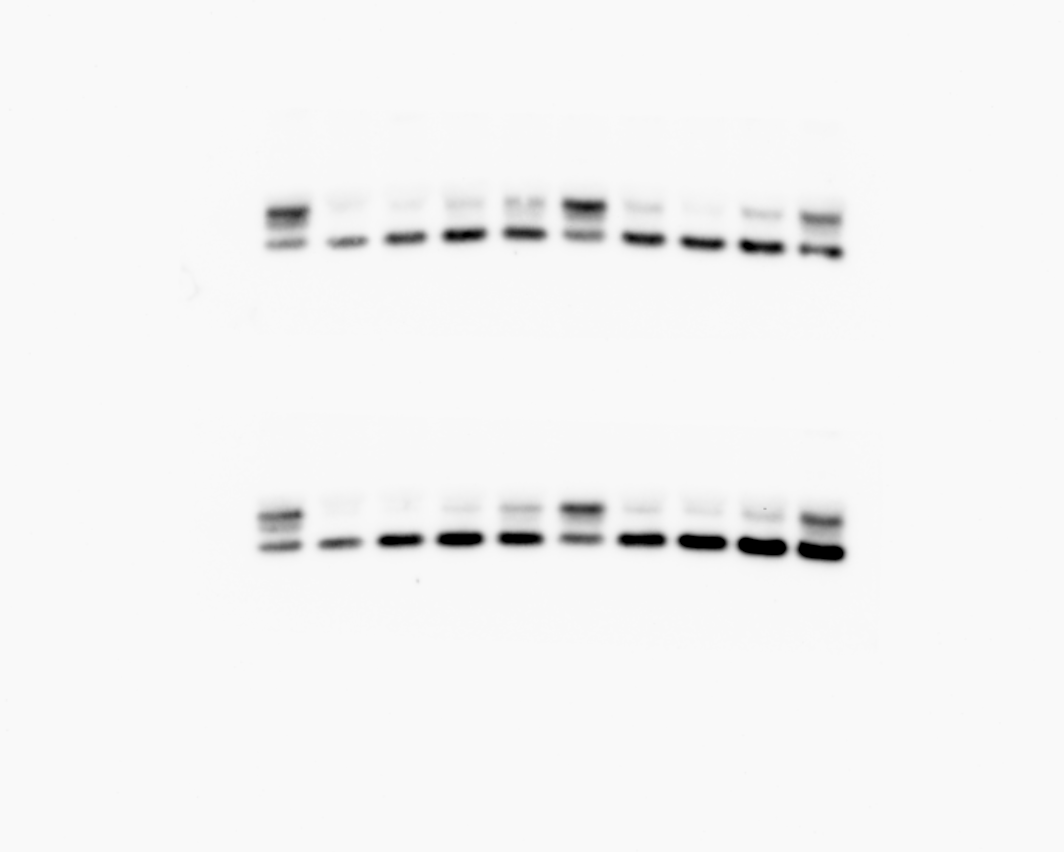

Supplement: Supplementary file 5 — Source data Fig. 2 [file 44318_2024_291_MOESM5_ESM.zip › SD Figure 2/EMBOJ-2023-115976_Fig2G-3_LC3B.tif]

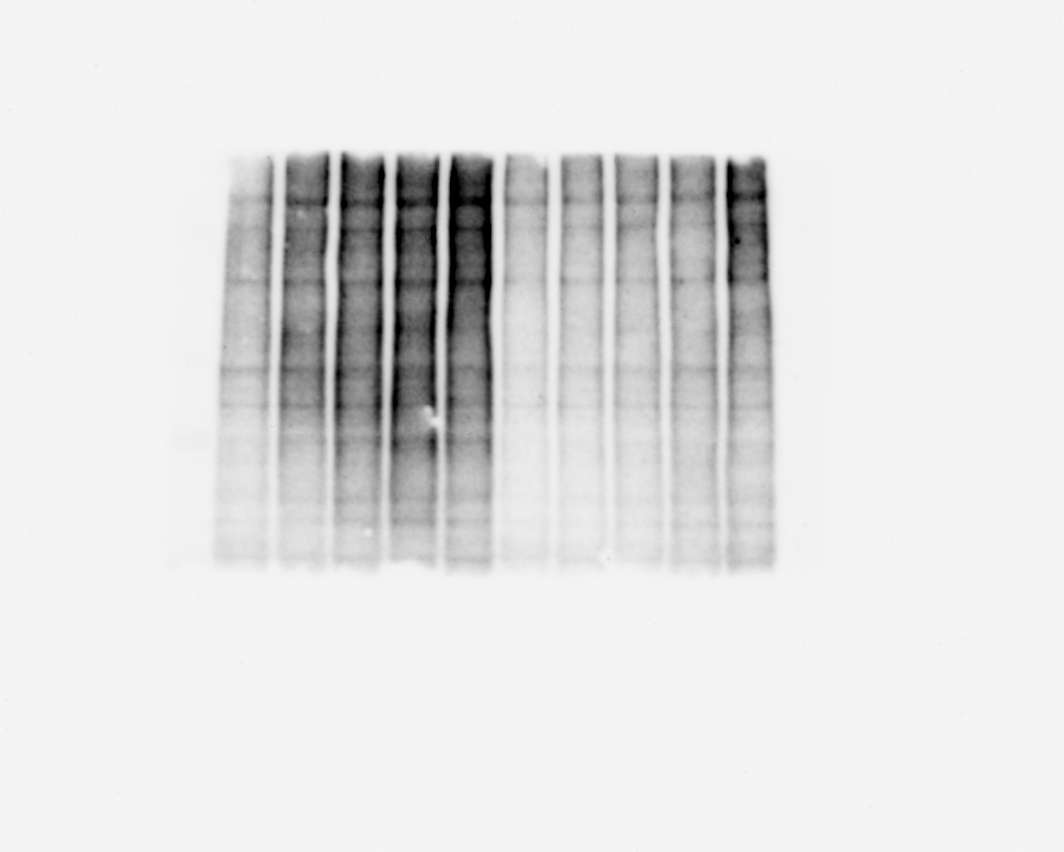

Supplement: Supplementary file 5 — Source data Fig. 2 [file 44318_2024_291_MOESM5_ESM.zip › SD Figure 2/EMBOJ-2023-115976_Fig2G-4_M1.tif]

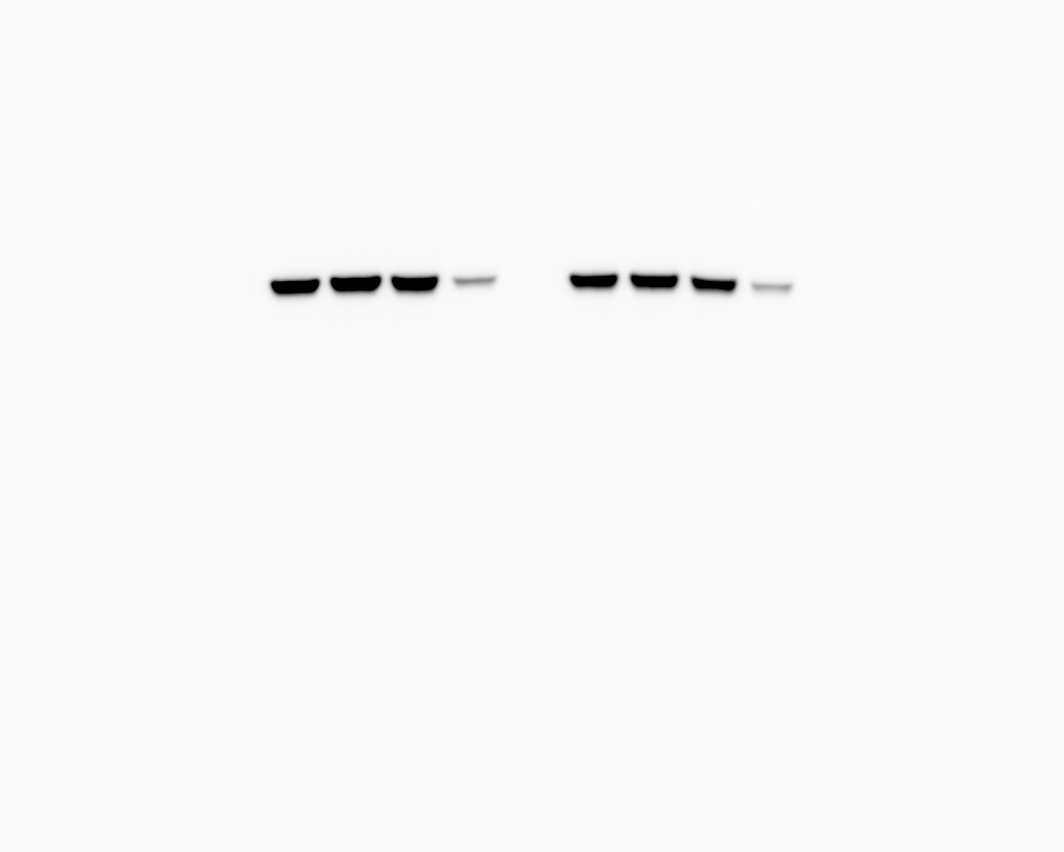

Supplement: Supplementary file 5 — Source data Fig. 2 [file 44318_2024_291_MOESM5_ESM.zip › SD Figure 2/EMBOJ-2023-115976_Fig2G-5_pSTING.tif]

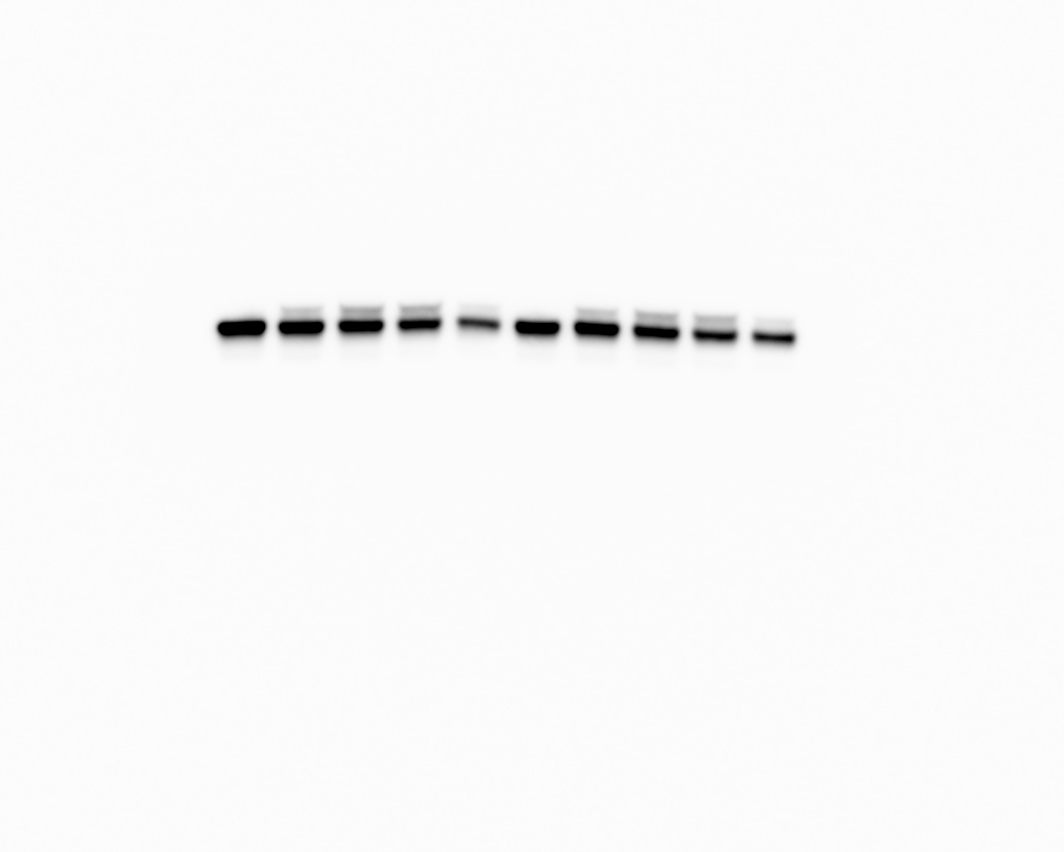

Supplement: Supplementary file 5 — Source data Fig. 2 [file 44318_2024_291_MOESM5_ESM.zip › SD Figure 2/EMBOJ-2023-115976_Fig2G-6_STING.tif]

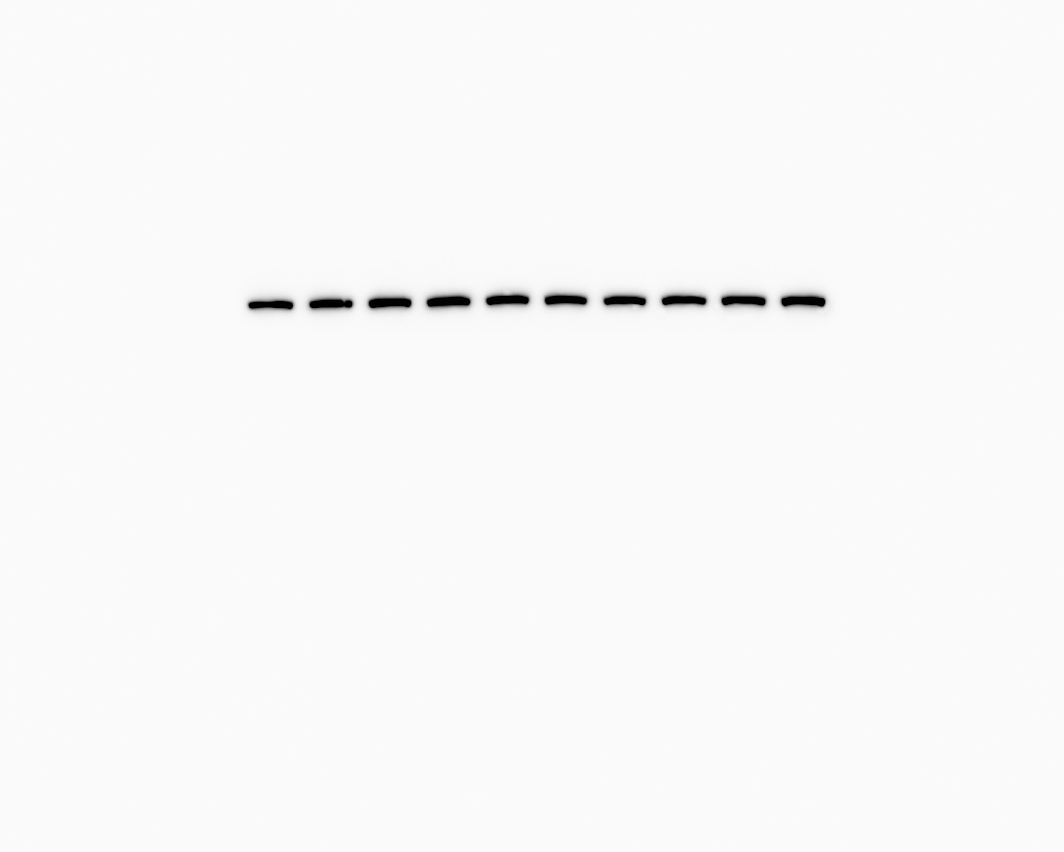

Supplement: Supplementary file 5 — Source data Fig. 2 [file 44318_2024_291_MOESM5_ESM.zip › SD Figure 2/EMBOJ-2023-115976_Fig2G-7_Vinc.tif]

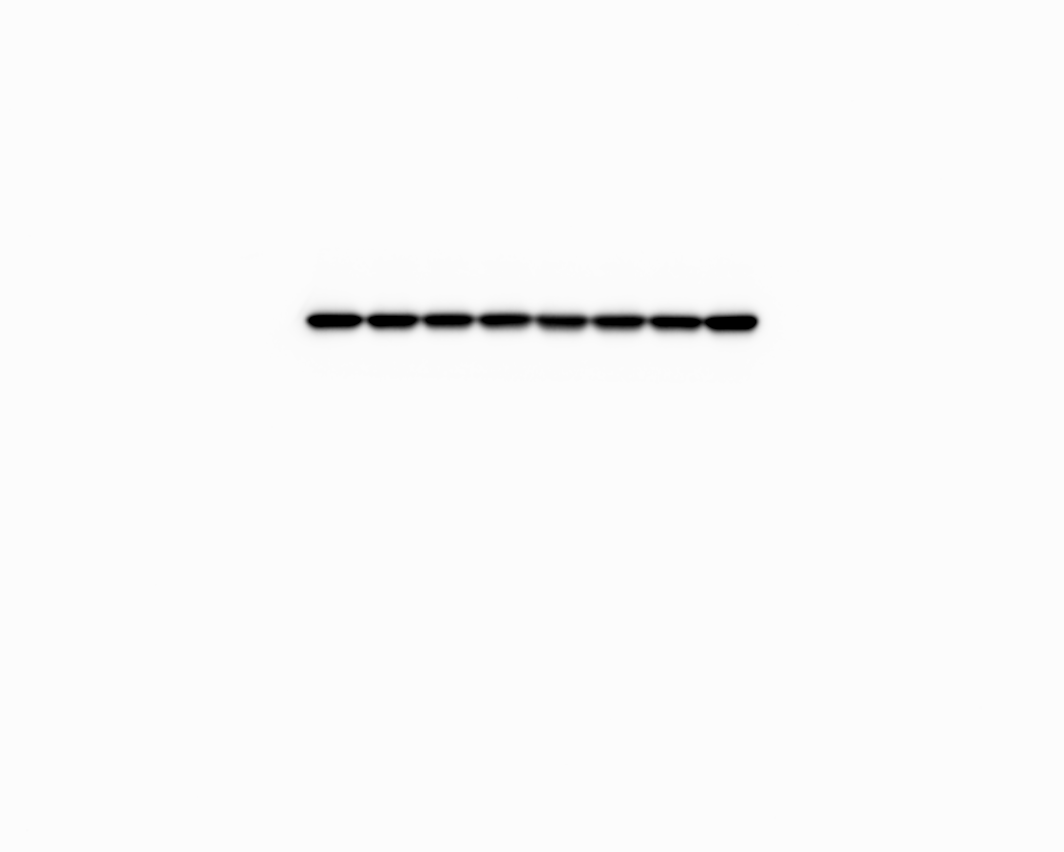

Supplement: Supplementary file 5 — Source data Fig. 2 [file 44318_2024_291_MOESM5_ESM.zip › SD Figure 2/EMBOJ-2023-115976_Fig2H-1_gapdh.tif]

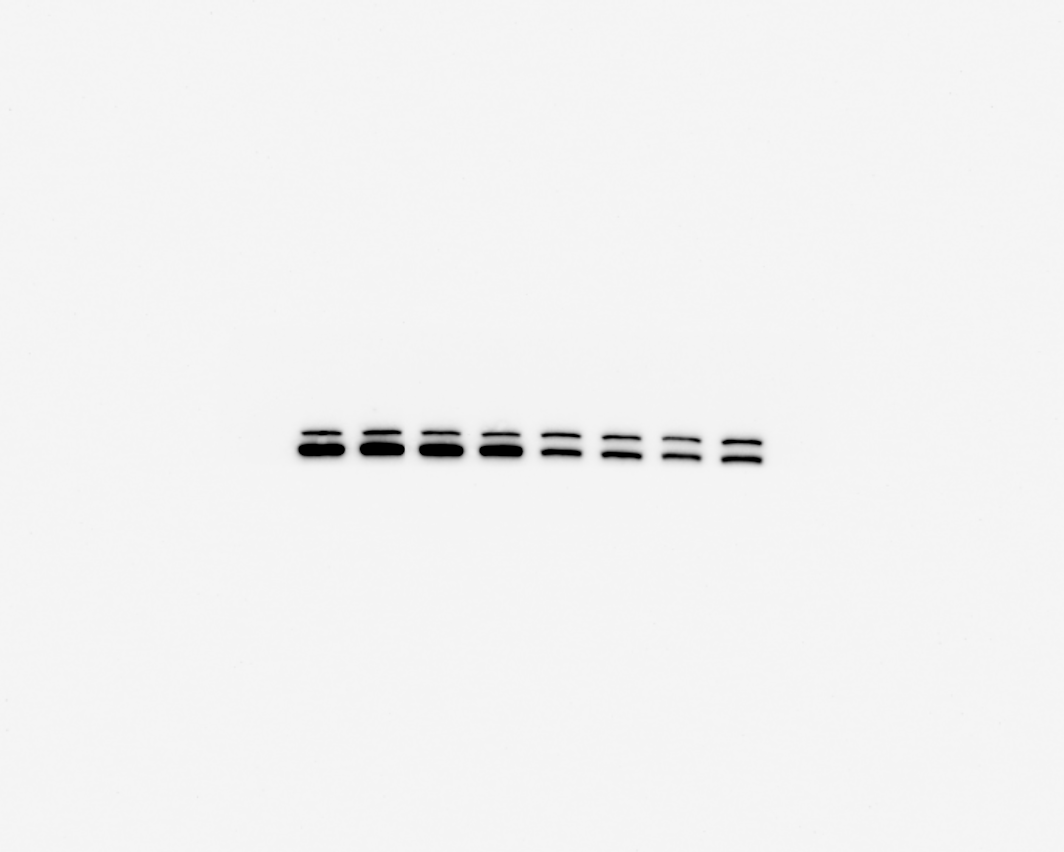

Supplement: Supplementary file 5 — Source data Fig. 2 [file 44318_2024_291_MOESM5_ESM.zip › SD Figure 2/EMBOJ-2023-115976_Fig2H-2_HOIP.tif]

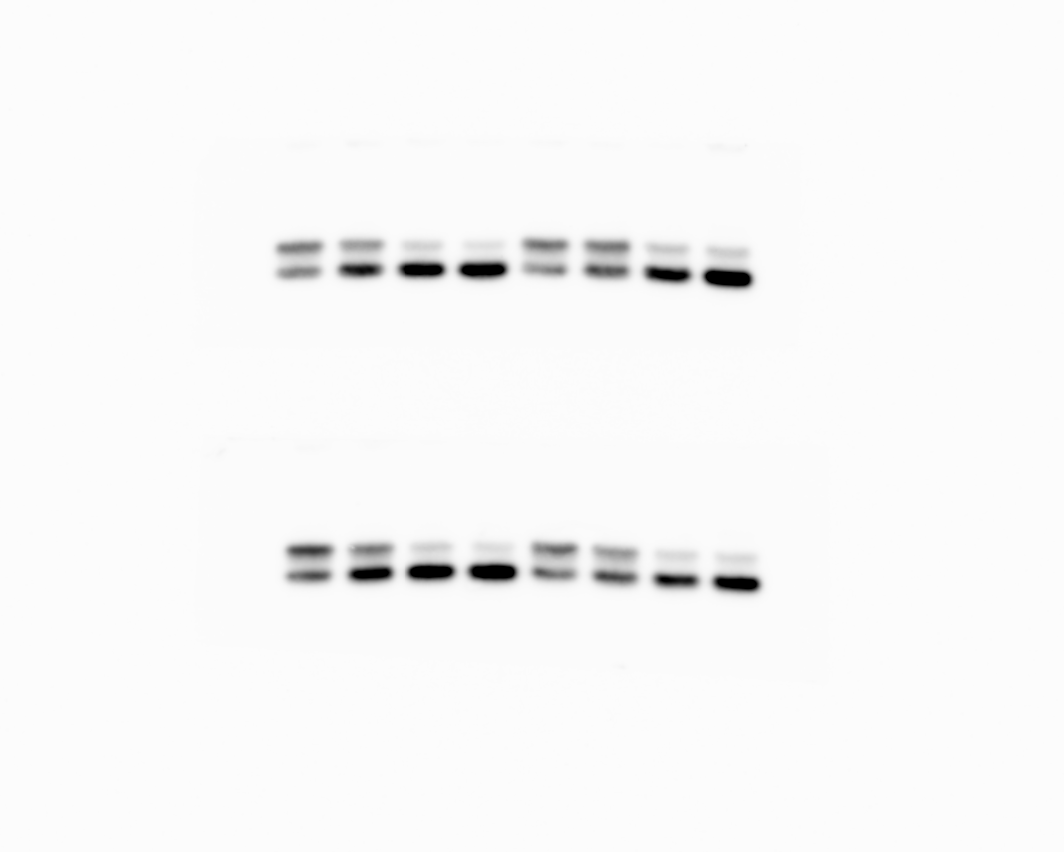

Supplement: Supplementary file 5 — Source data Fig. 2 [file 44318_2024_291_MOESM5_ESM.zip › SD Figure 2/EMBOJ-2023-115976_Fig2H-3_LC3B.tif]

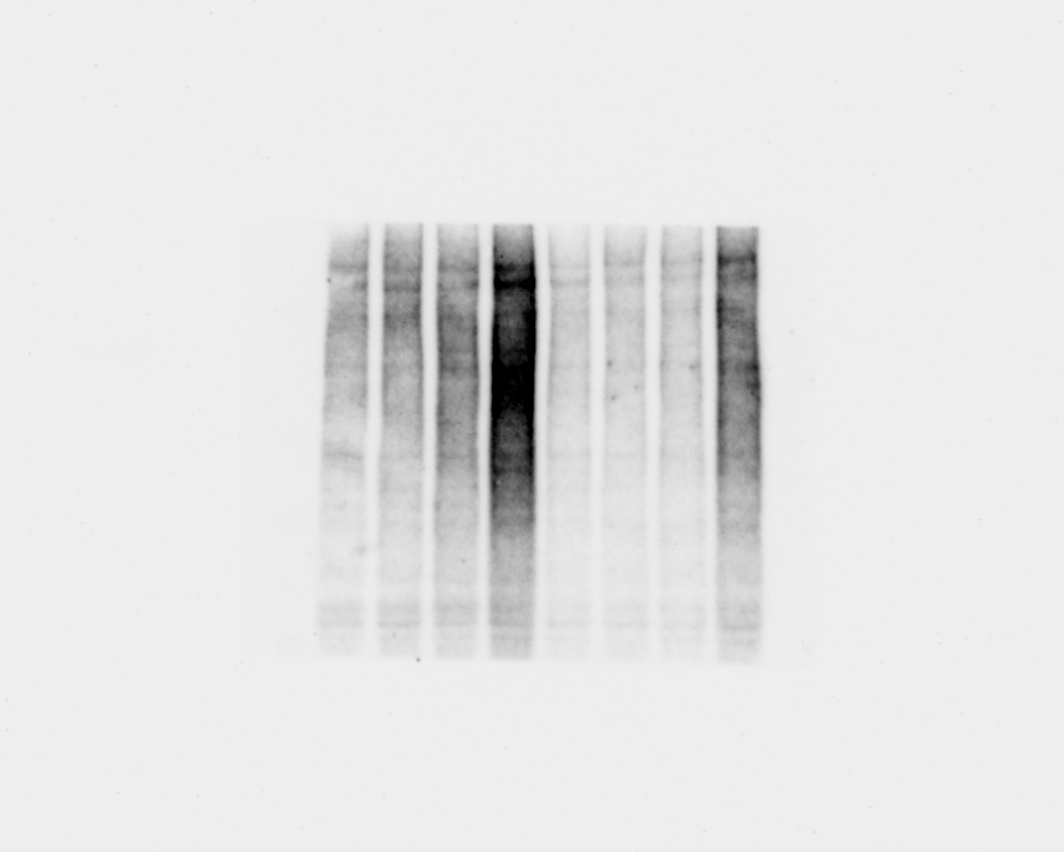

Supplement: Supplementary file 5 — Source data Fig. 2 [file 44318_2024_291_MOESM5_ESM.zip › SD Figure 2/EMBOJ-2023-115976_Fig2H-4_M1.tif]

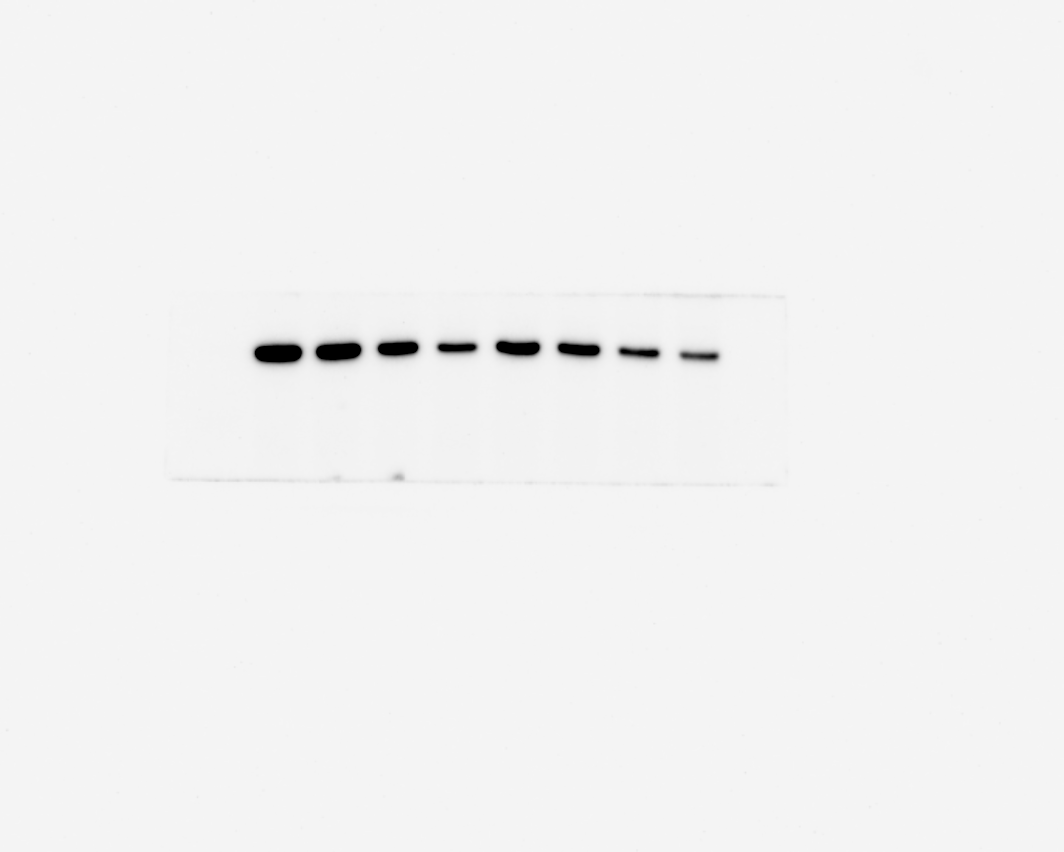

Supplement: Supplementary file 5 — Source data Fig. 2 [file 44318_2024_291_MOESM5_ESM.zip › SD Figure 2/EMBOJ-2023-115976_Fig2H-5_STING.tif]

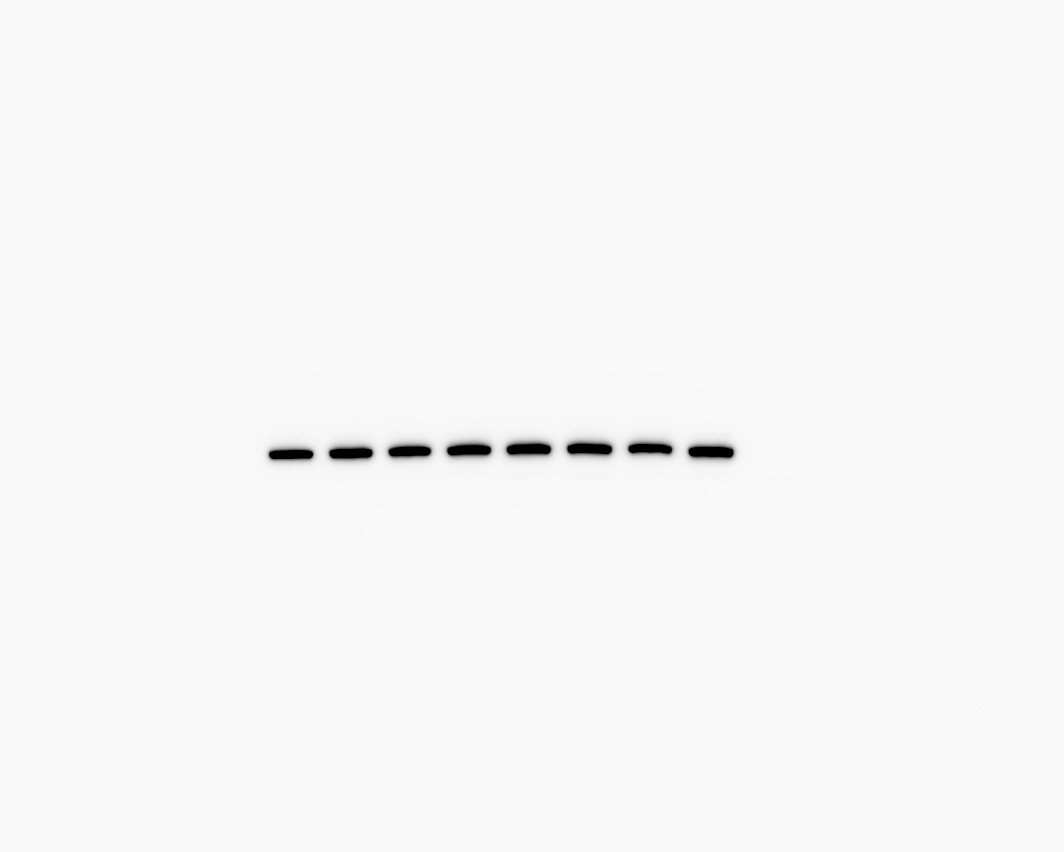

Supplement: Supplementary file 5 — Source data Fig. 2 [file 44318_2024_291_MOESM5_ESM.zip › SD Figure 2/EMBOJ-2023-115976_Fig2H-6_Vin.tif]

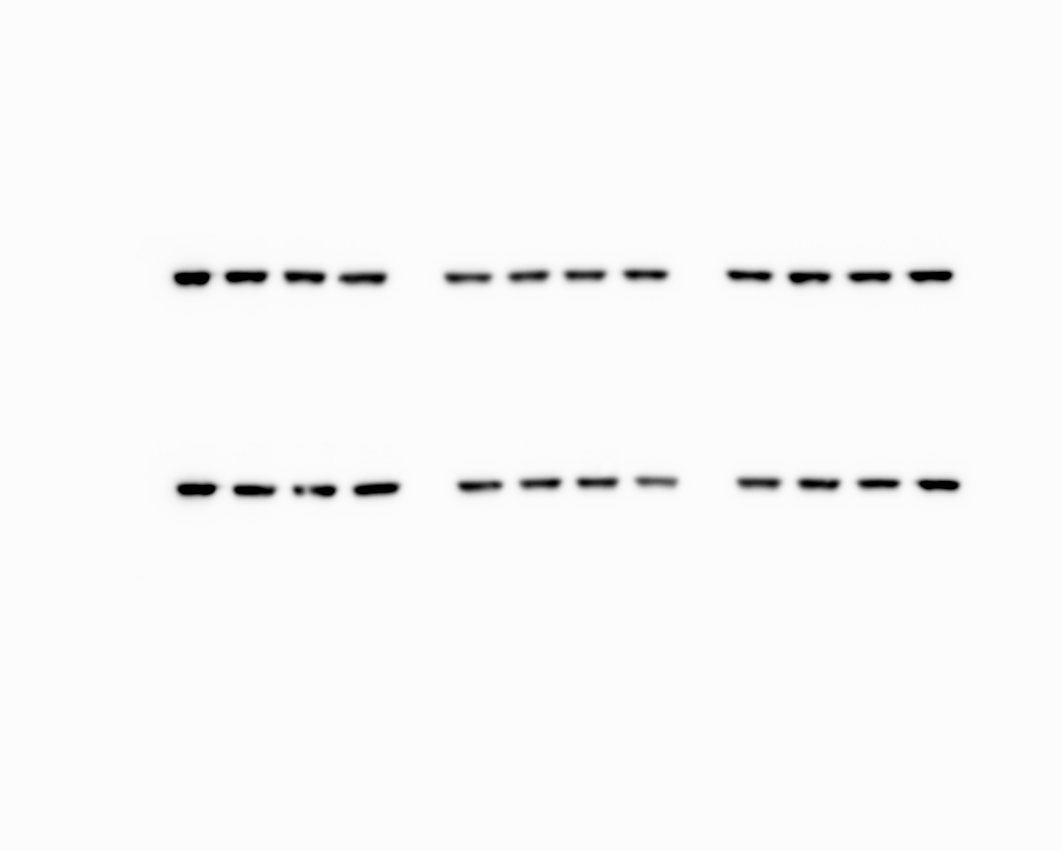

Supplement: Supplementary file 6 — Source data Fig. 3 [file 44318_2024_291_MOESM6_ESM.zip › SD Figure 3/EMBOJ-2023-115976_Fig3A-1_gapdh.tif]

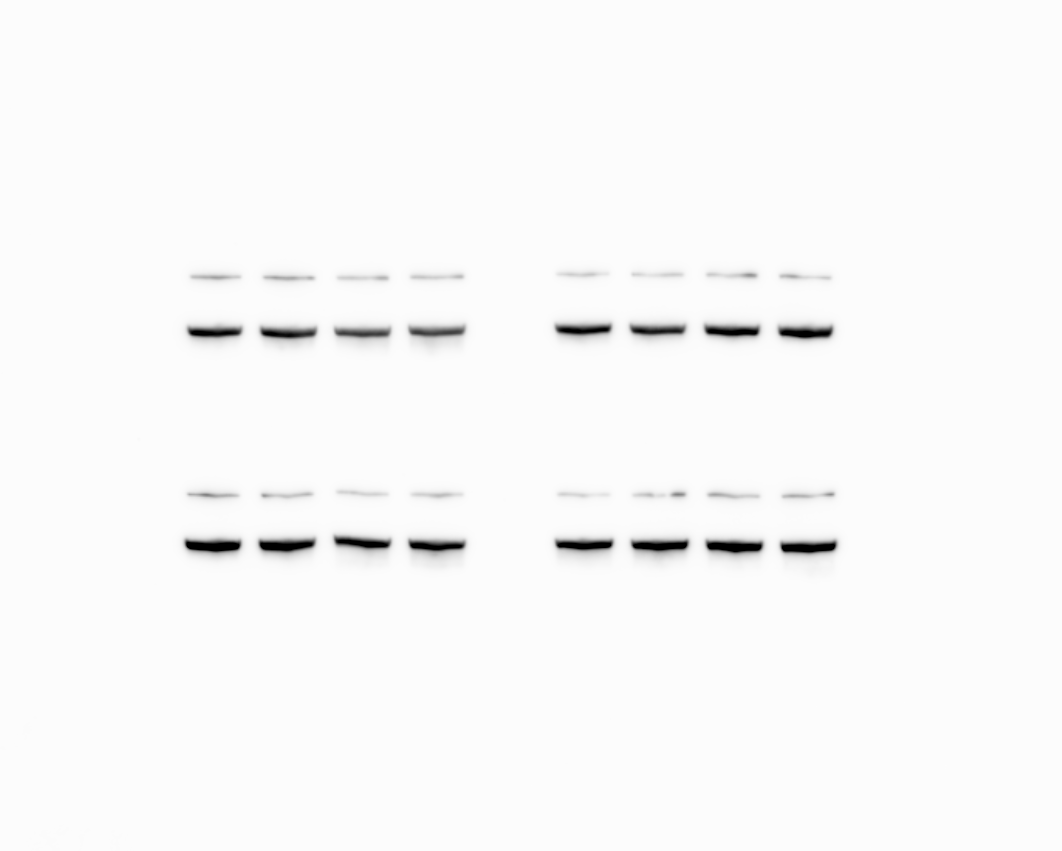

Supplement: Supplementary file 6 — Source data Fig. 3 [file 44318_2024_291_MOESM6_ESM.zip › SD Figure 3/EMBOJ-2023-115976_Fig3A-2_HSP90.tif]

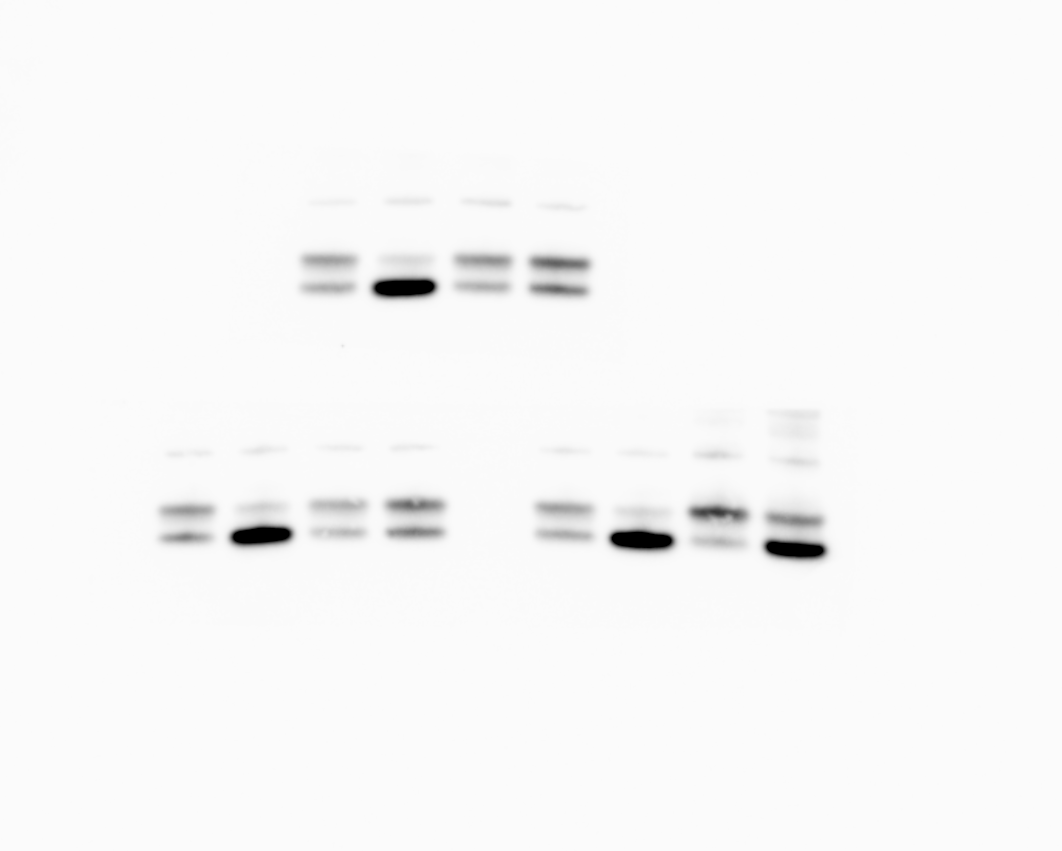

Supplement: Supplementary file 6 — Source data Fig. 3 [file 44318_2024_291_MOESM6_ESM.zip › SD Figure 3/EMBOJ-2023-115976_Fig3A-3_LC3B.tif]

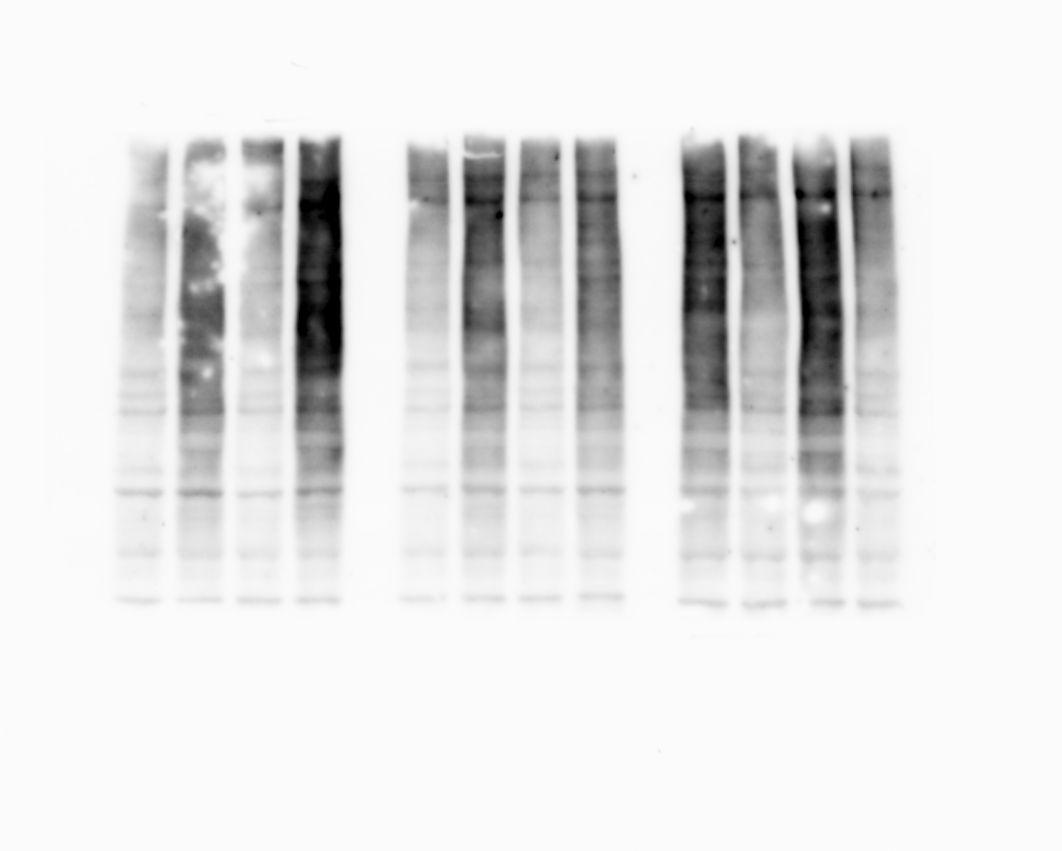

Supplement: Supplementary file 6 — Source data Fig. 3 [file 44318_2024_291_MOESM6_ESM.zip › SD Figure 3/EMBOJ-2023-115976_Fig3A-4_M1.tif]

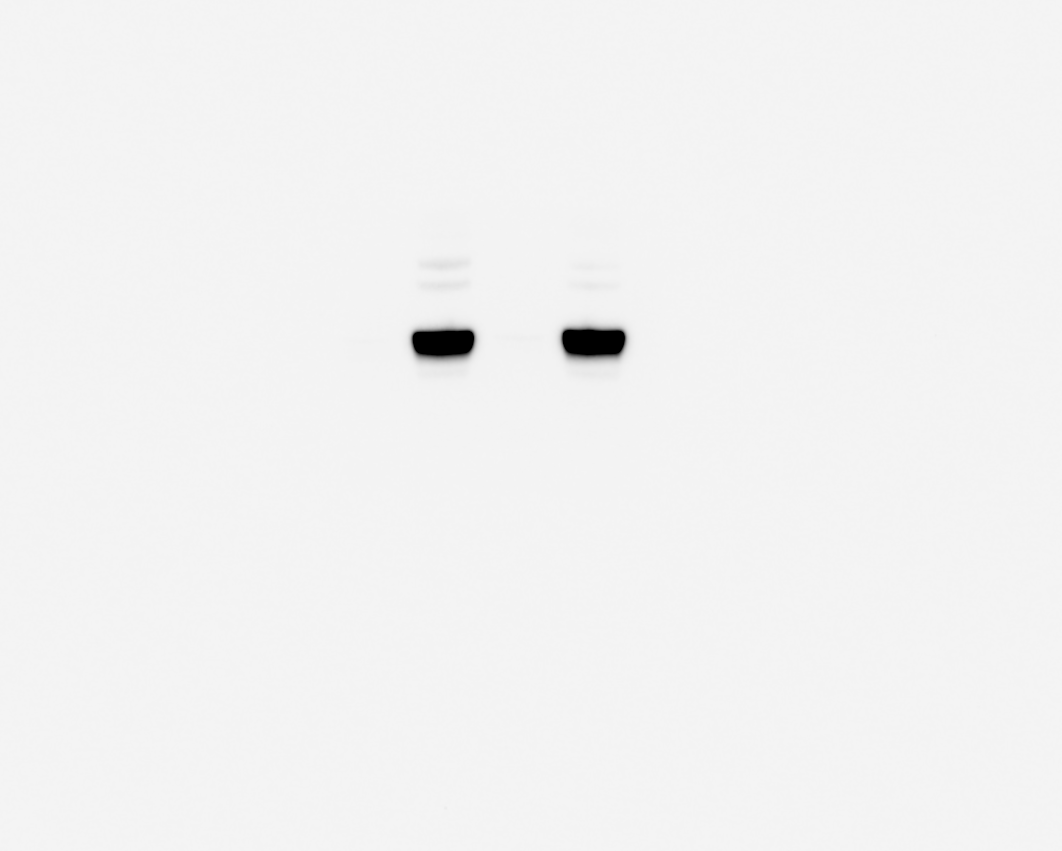

Supplement: Supplementary file 6 — Source data Fig. 3 [file 44318_2024_291_MOESM6_ESM.zip › SD Figure 3/EMBOJ-2023-115976_Fig3A-5_pSTING.tif]

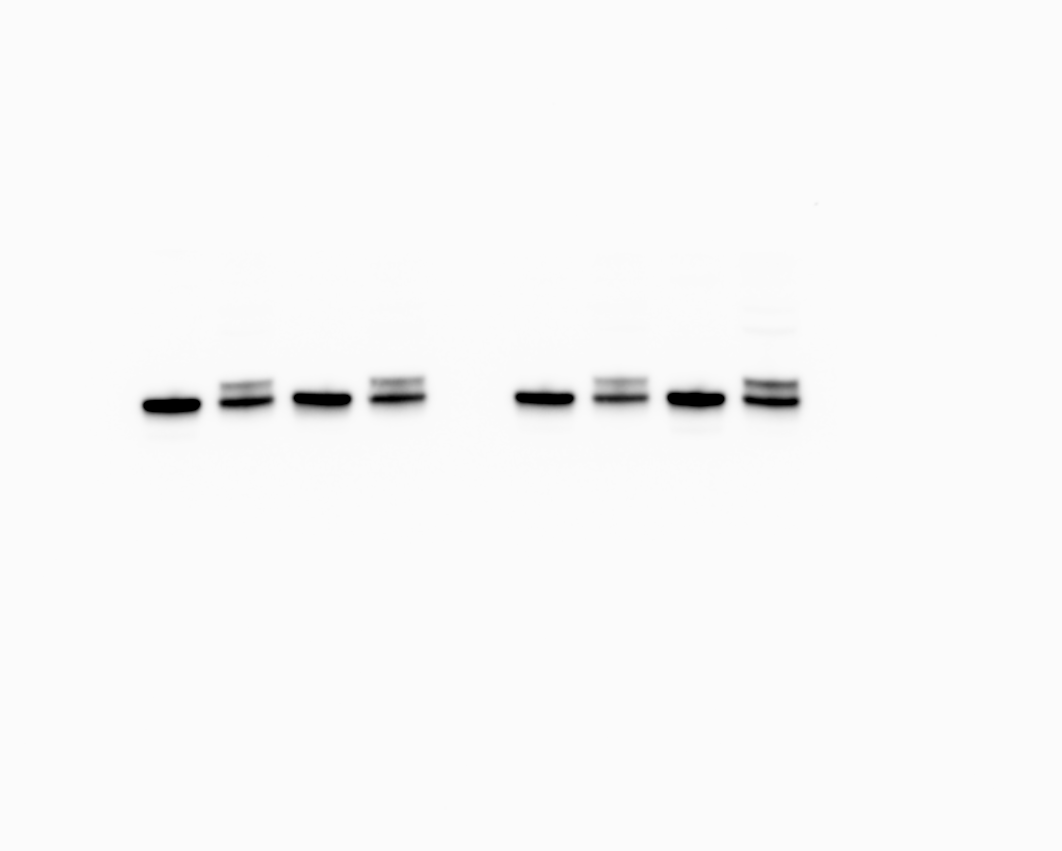

Supplement: Supplementary file 6 — Source data Fig. 3 [file 44318_2024_291_MOESM6_ESM.zip › SD Figure 3/EMBOJ-2023-115976_Fig3A-6_STING.tif]

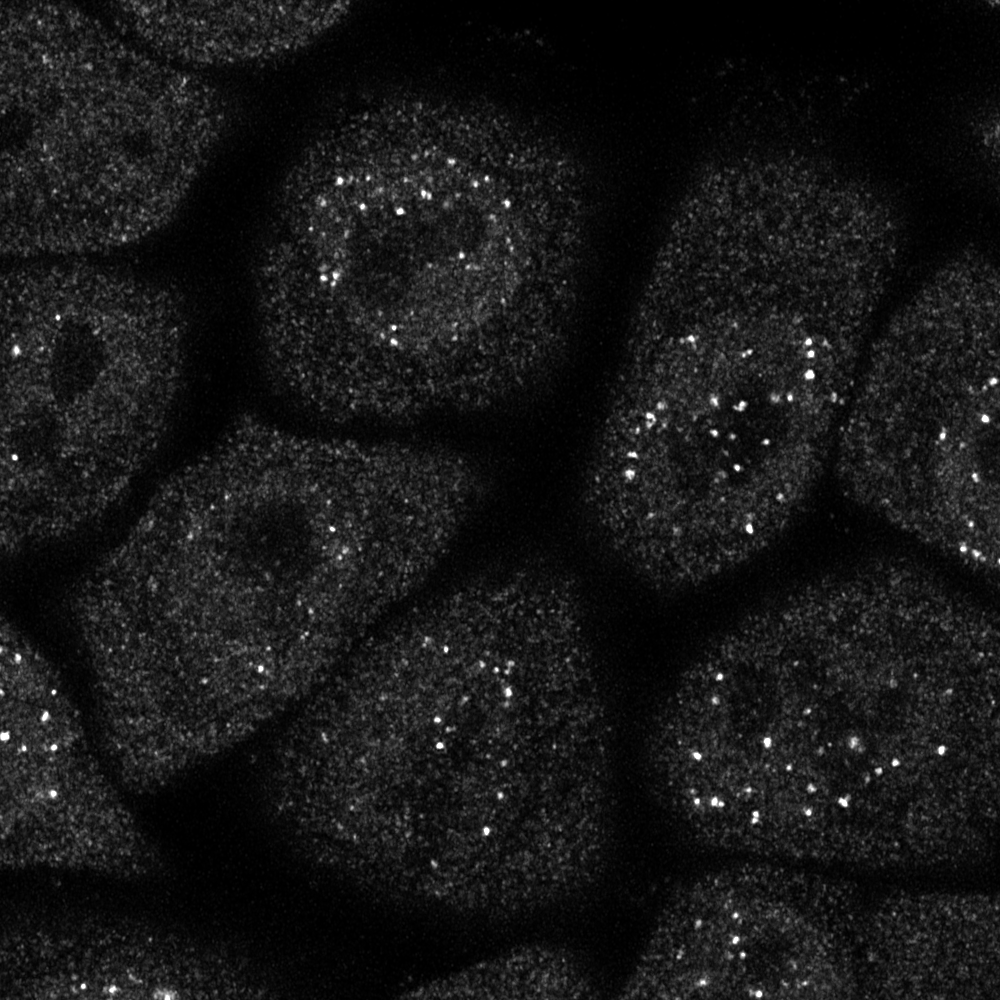

Supplement: Supplementary file 6 — Source data Fig. 3 [file 44318_2024_291_MOESM6_ESM.zip › SD Figure 3/EMBOJ-2023-115976_Fig3C-1_WT-nt.tif]

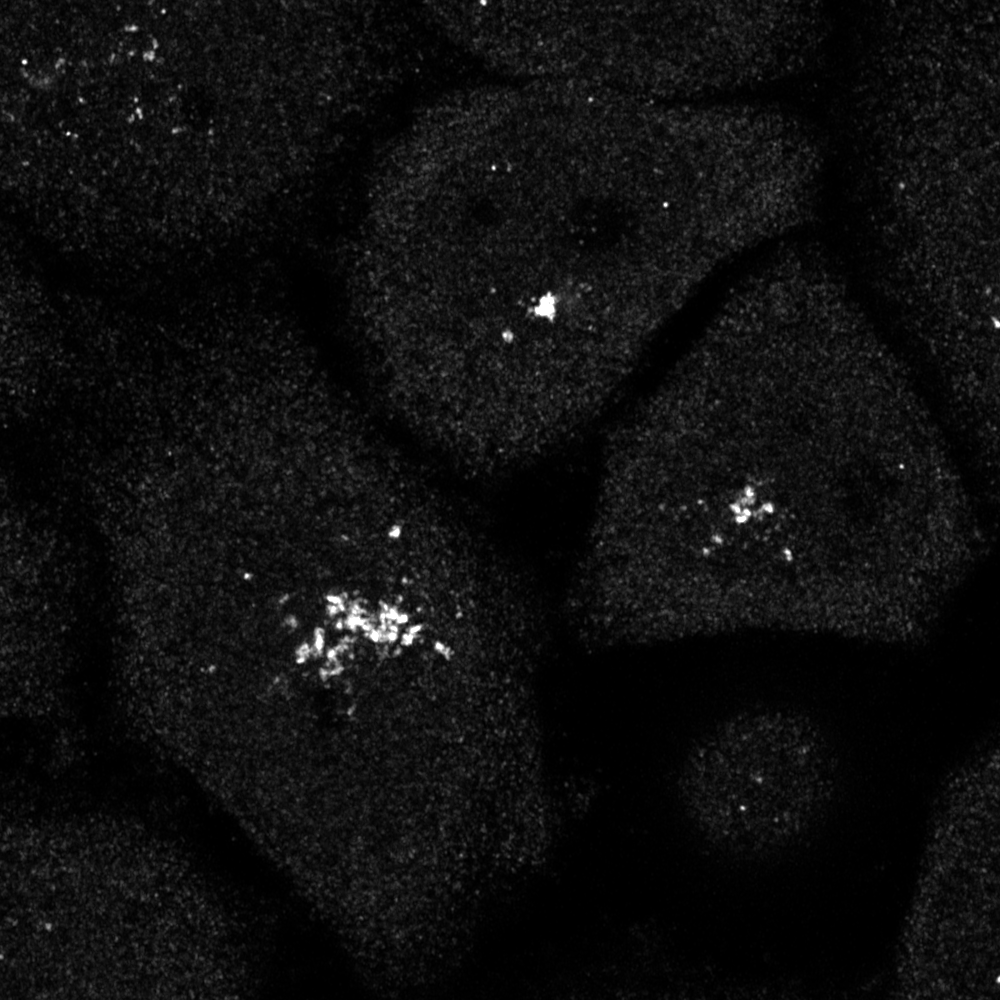

Supplement: Supplementary file 6 — Source data Fig. 3 [file 44318_2024_291_MOESM6_ESM.zip › SD Figure 3/EMBOJ-2023-115976_Fig3C-2_WT-cGAMP.tif]

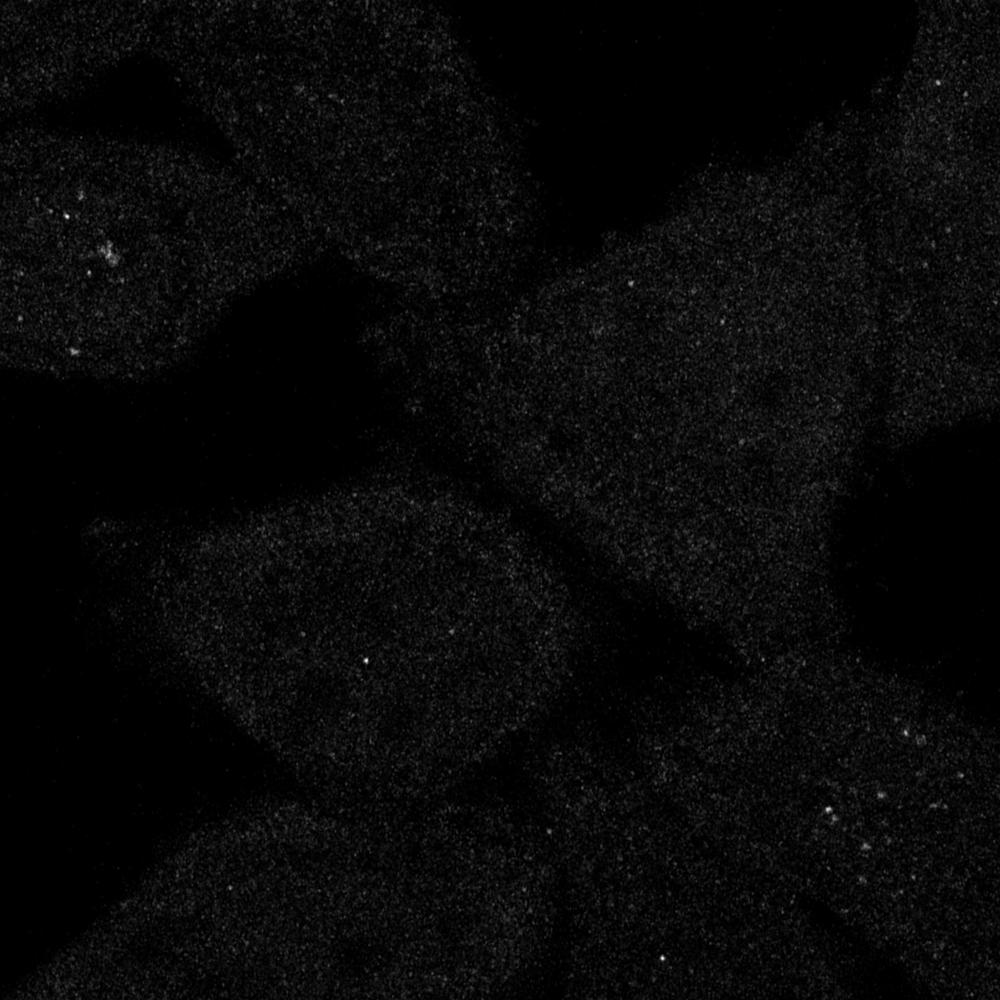

Supplement: Supplementary file 6 — Source data Fig. 3 [file 44318_2024_291_MOESM6_ESM.zip › SD Figure 3/EMBOJ-2023-115976_Fig3C-3_SopF-nt.tif]

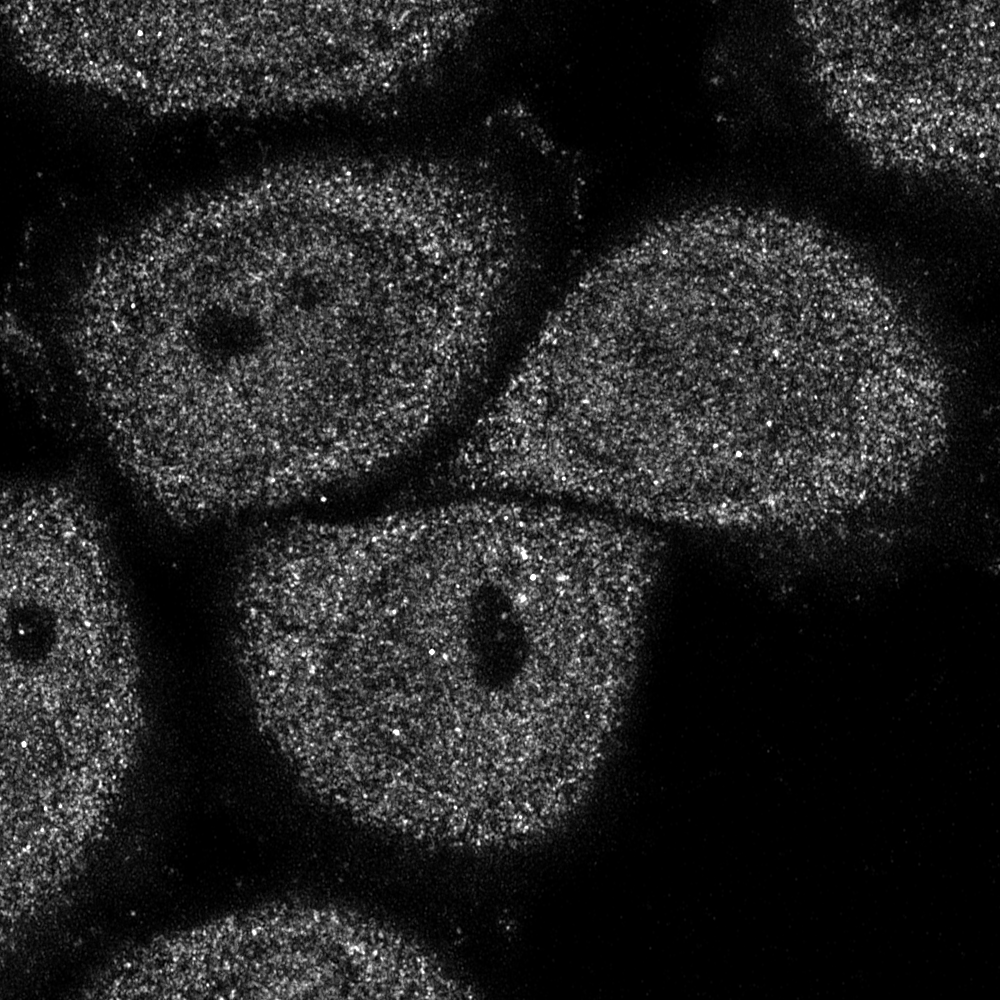

Supplement: Supplementary file 6 — Source data Fig. 3 [file 44318_2024_291_MOESM6_ESM.zip › SD Figure 3/EMBOJ-2023-115976_Fig3C-4_SopF-cGAMP.tif]

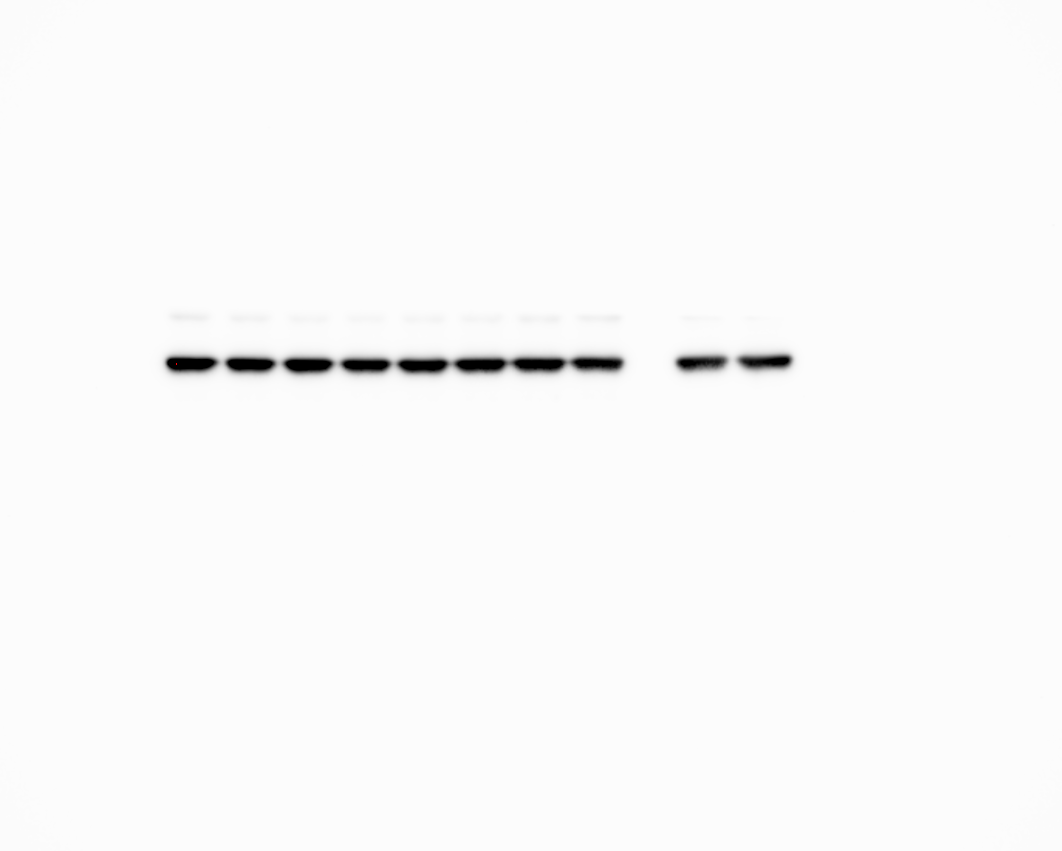

Supplement: Supplementary file 6 — Source data Fig. 3 [file 44318_2024_291_MOESM6_ESM.zip › SD Figure 3/EMBOJ-2023-115976_Fig3D-1_gapdh.tif]

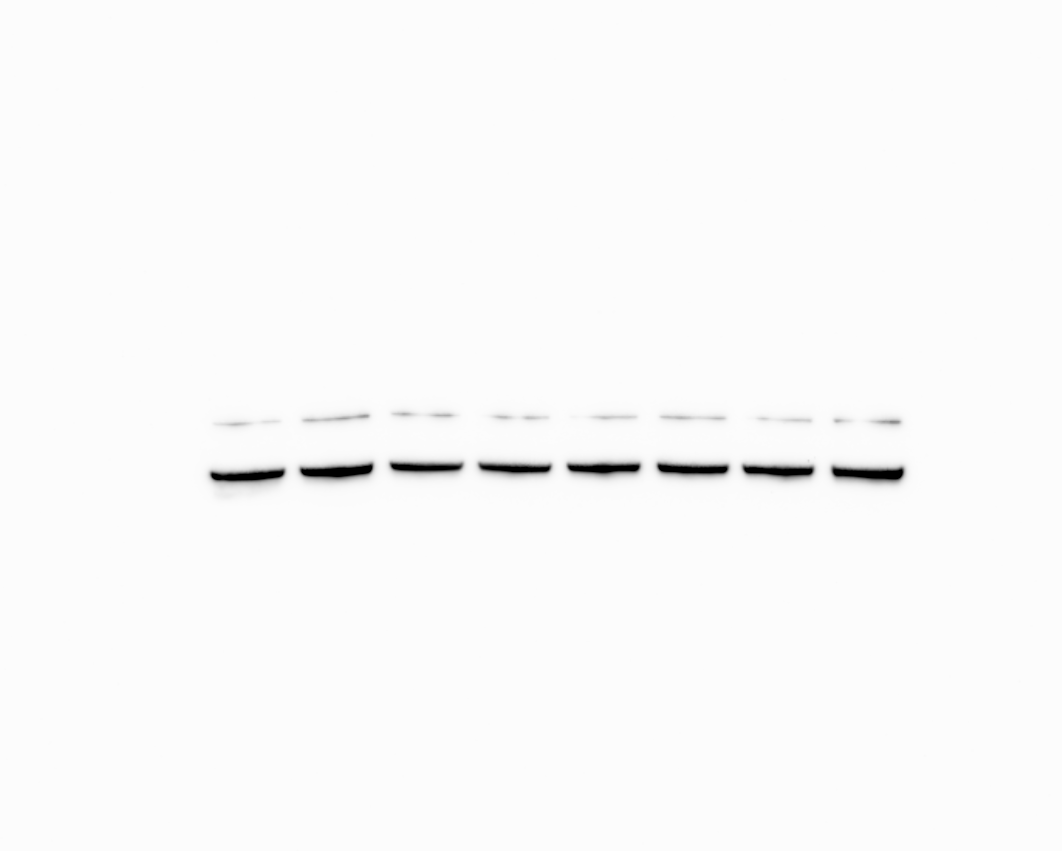

Supplement: Supplementary file 6 — Source data Fig. 3 [file 44318_2024_291_MOESM6_ESM.zip › SD Figure 3/EMBOJ-2023-115976_Fig3D-2_hsp90.tif]

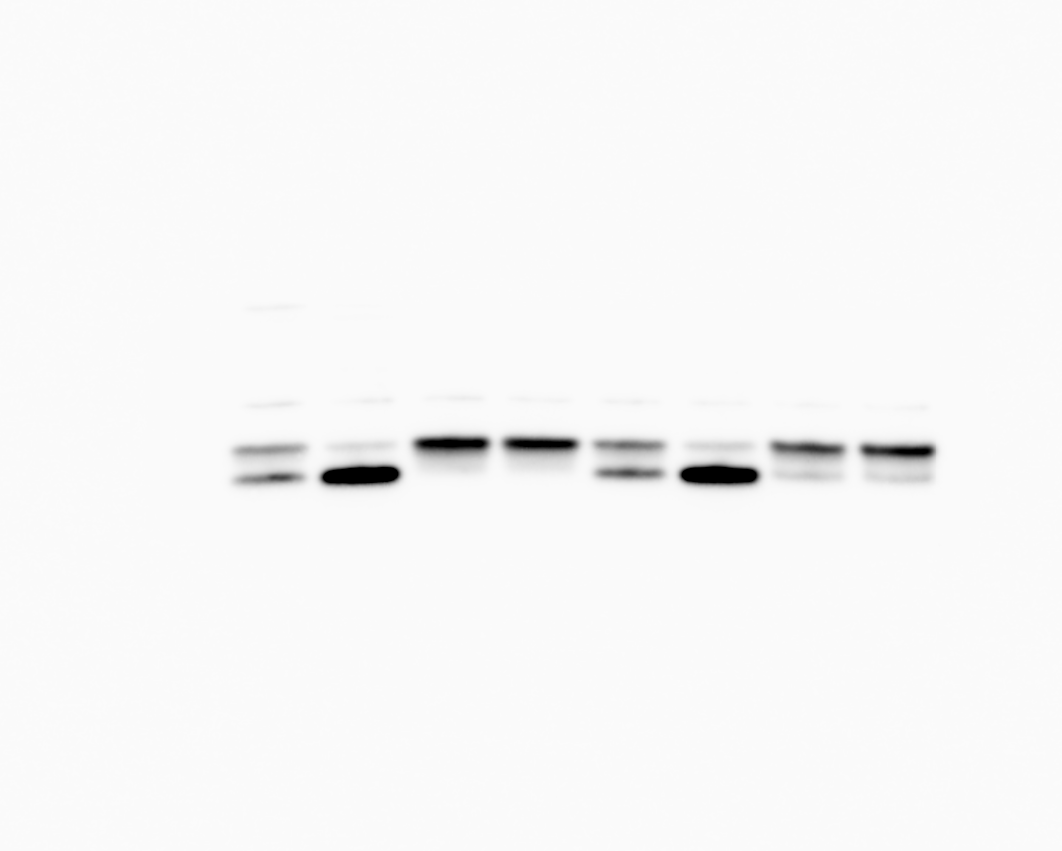

Supplement: Supplementary file 6 — Source data Fig. 3 [file 44318_2024_291_MOESM6_ESM.zip › SD Figure 3/EMBOJ-2023-115976_Fig3D-3_lc3b.tif]

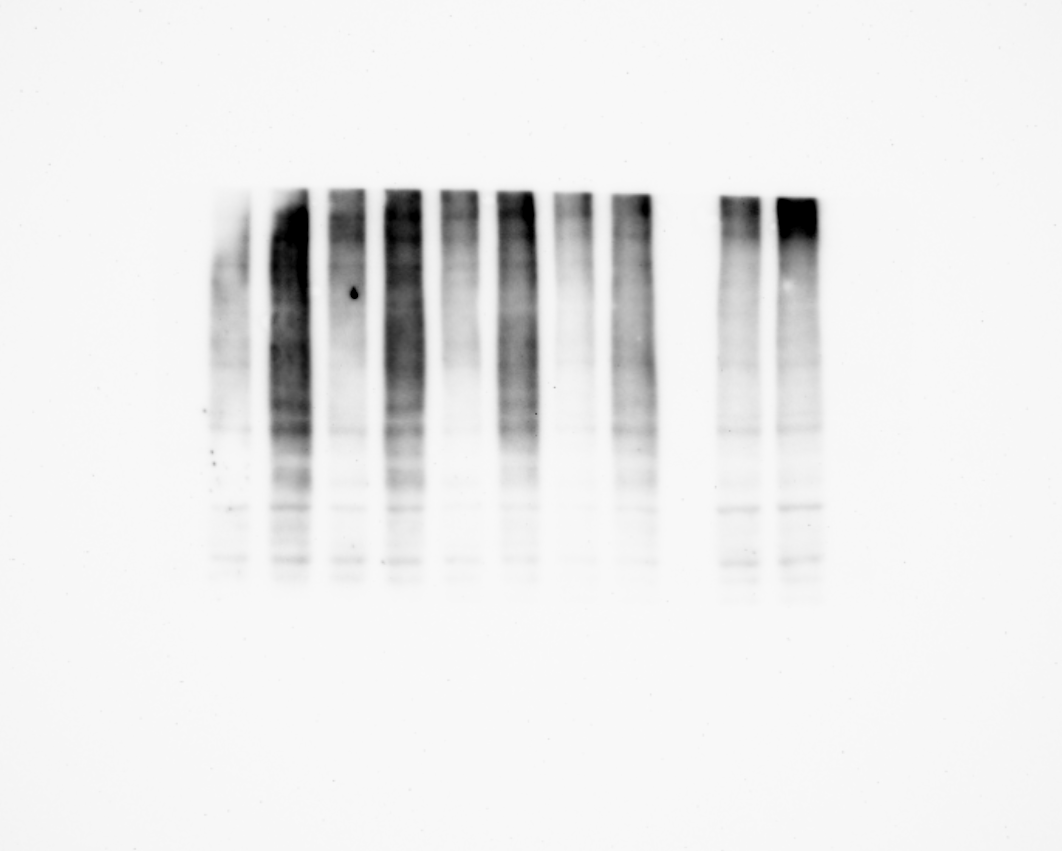

Supplement: Supplementary file 6 — Source data Fig. 3 [file 44318_2024_291_MOESM6_ESM.zip › SD Figure 3/EMBOJ-2023-115976_Fig3D-4_M1.tif]

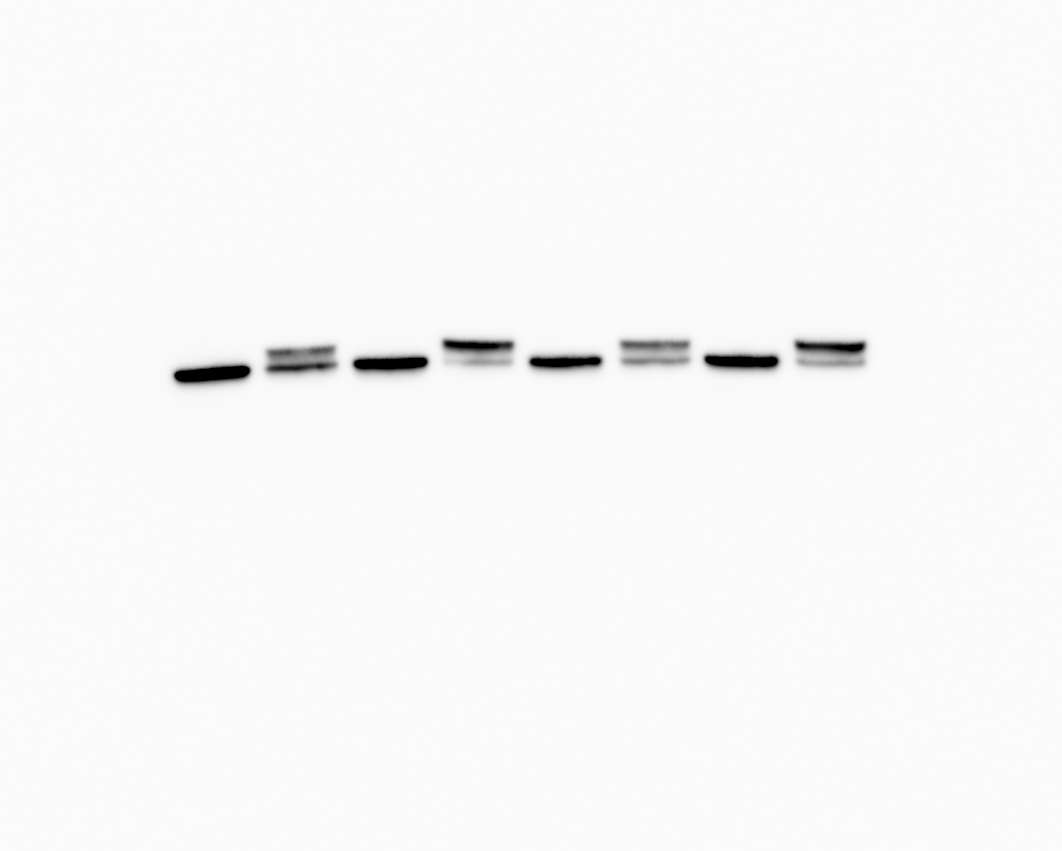

Supplement: Supplementary file 6 — Source data Fig. 3 [file 44318_2024_291_MOESM6_ESM.zip › SD Figure 3/EMBOJ-2023-115976_Fig3D-5_sting.tif]

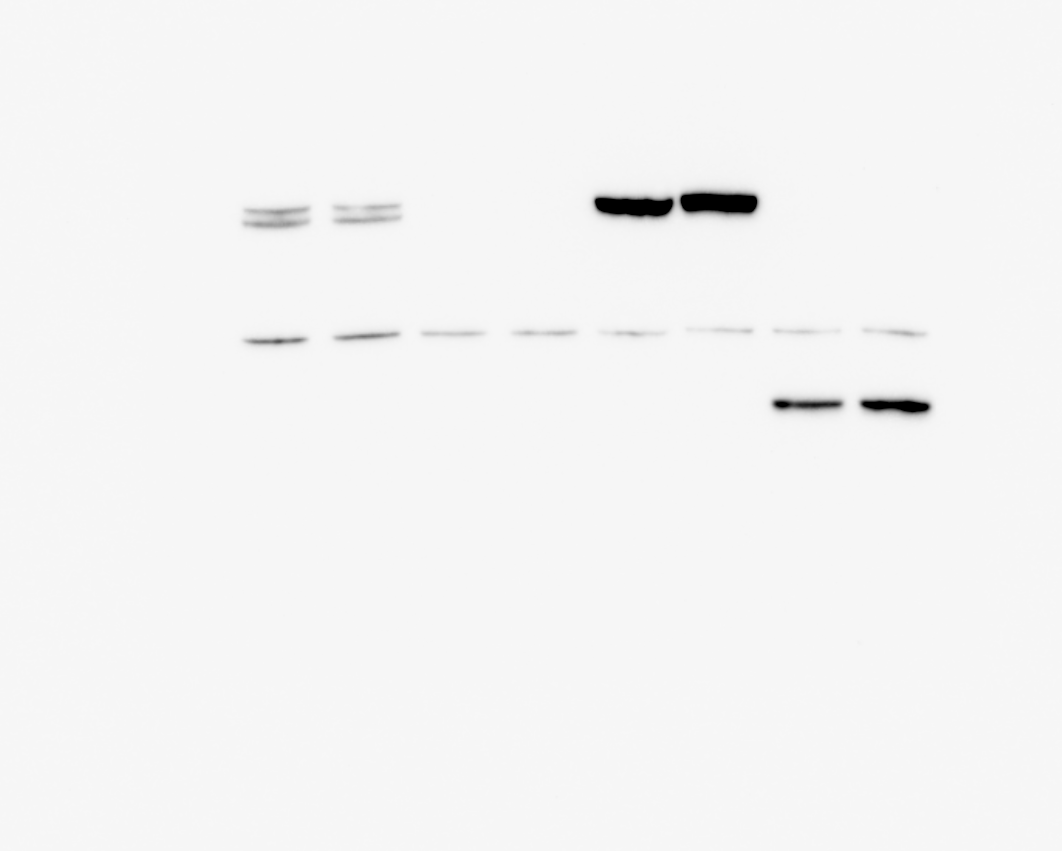

Supplement: Supplementary file 6 — Source data Fig. 3 [file 44318_2024_291_MOESM6_ESM.zip › SD Figure 3/EMBOJ-2023-115976_Fig3D-6_ATG16.tif]

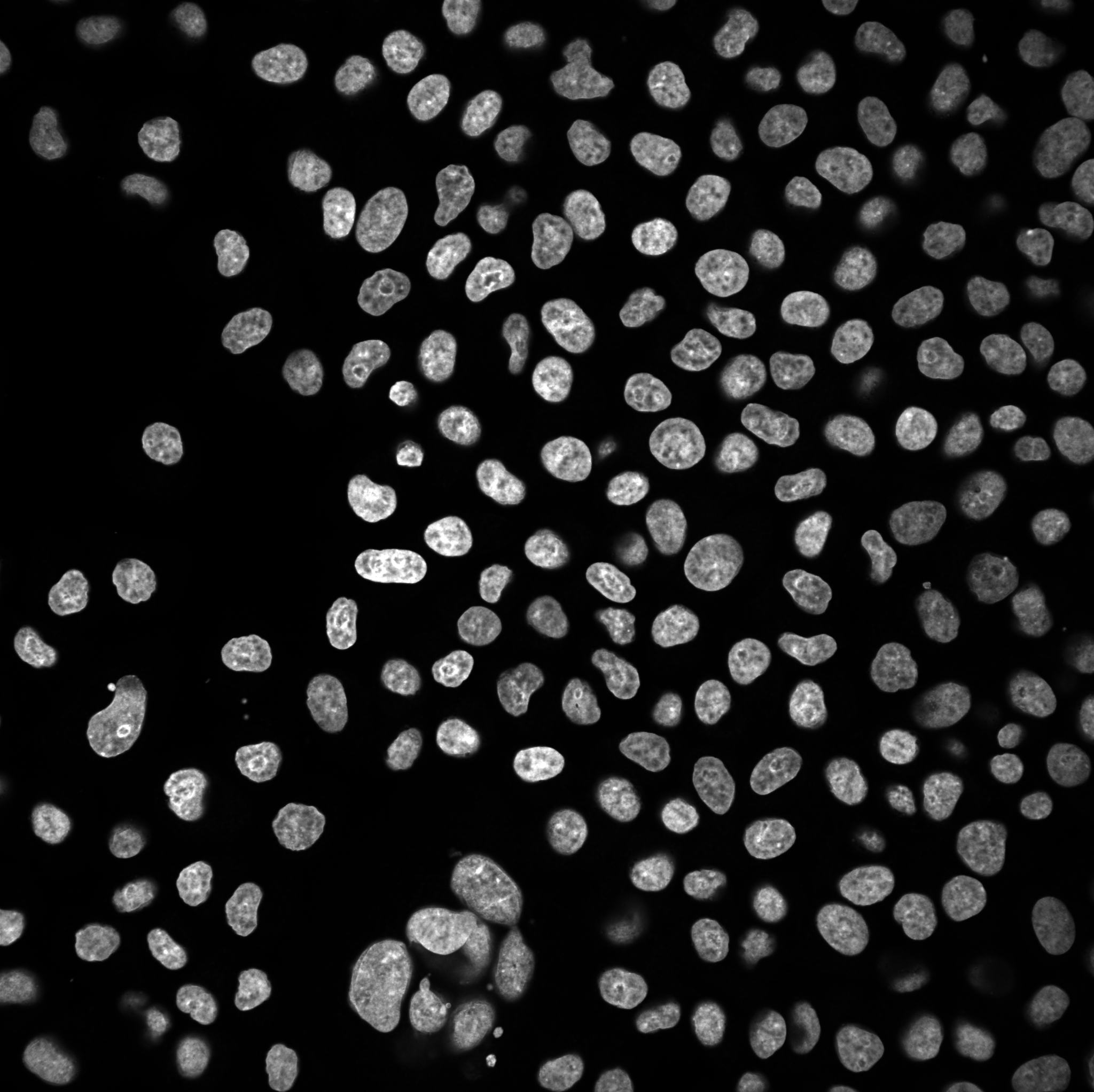

Supplement: Supplementary file 6 — Source data Fig. 3 [file 44318_2024_291_MOESM6_ESM.zip › SD Figure 3/EMBOJ-2023-115976_Fig3F-1_WT.tif]

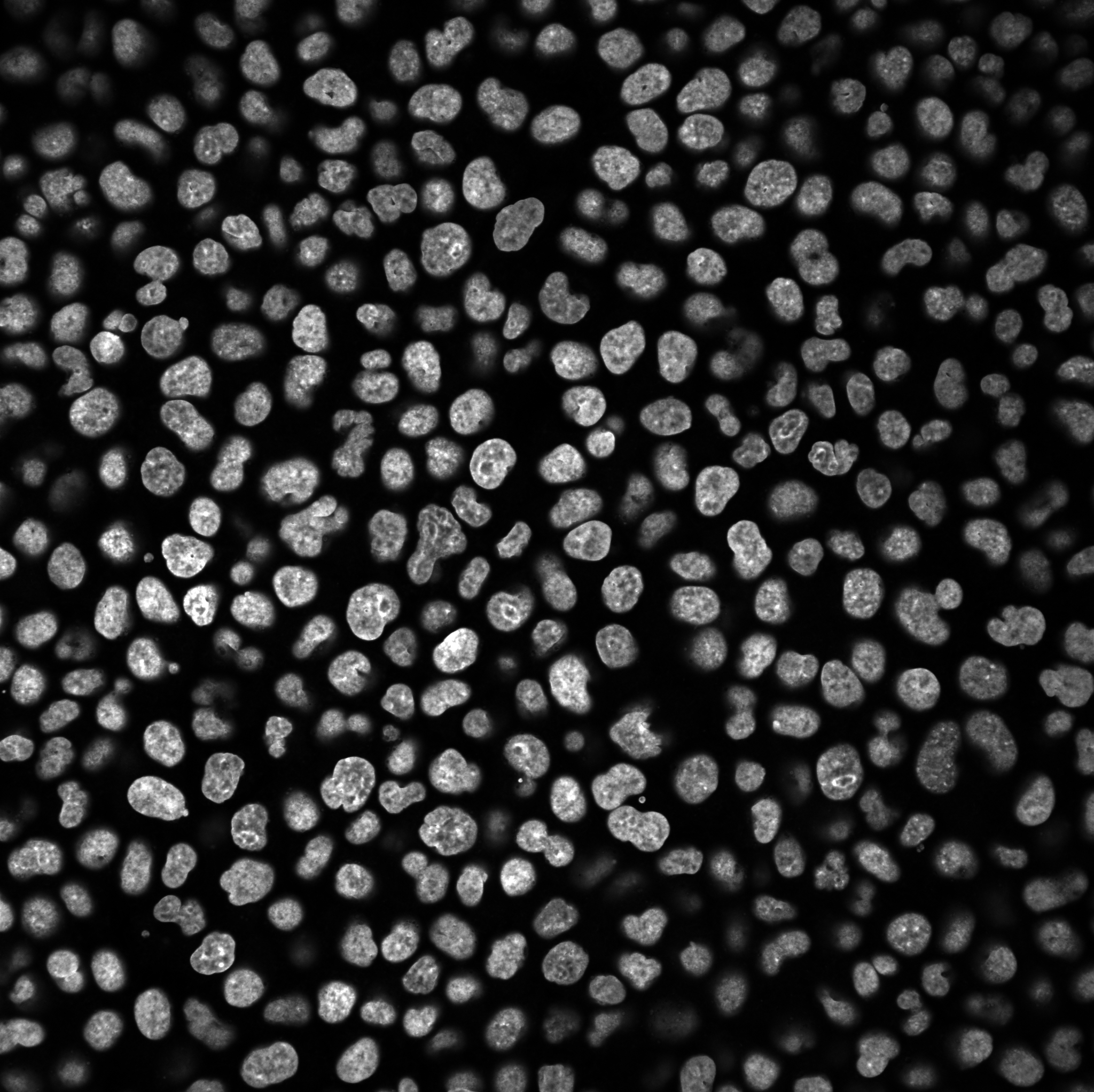

Supplement: Supplementary file 6 — Source data Fig. 3 [file 44318_2024_291_MOESM6_ESM.zip › SD Figure 3/EMBOJ-2023-115976_Fig3F-2_16KO.tif]

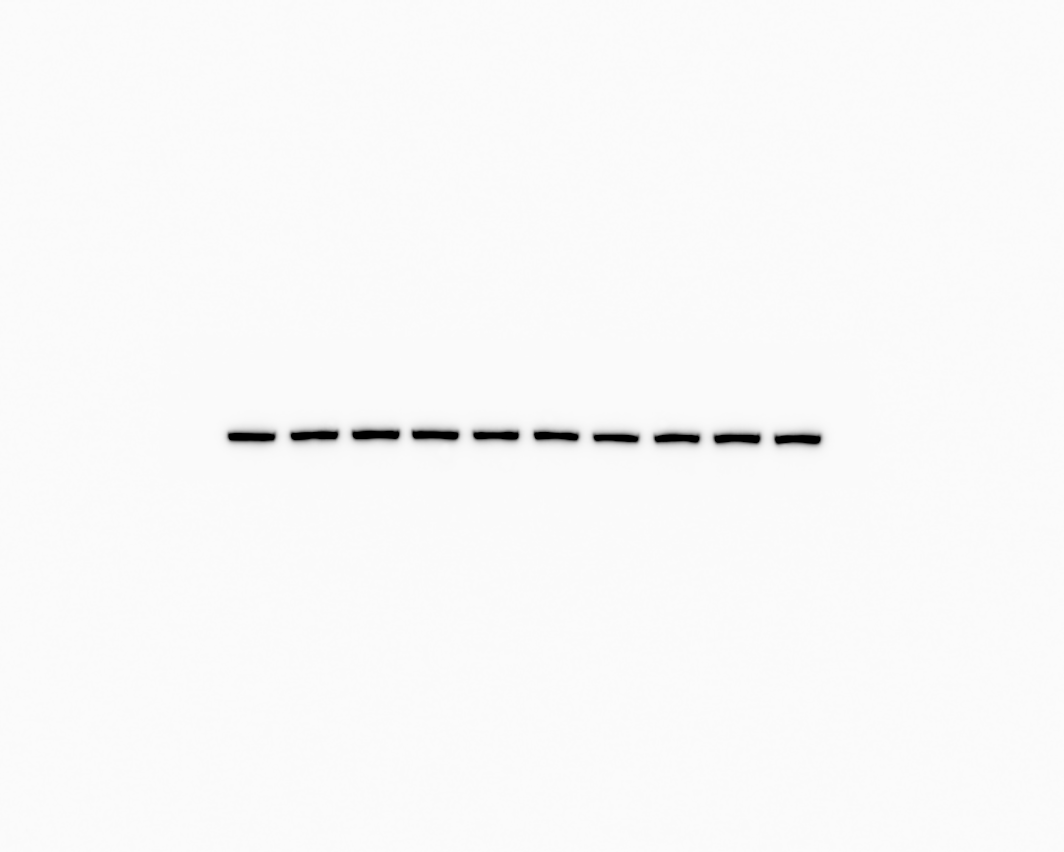

Supplement: Supplementary file 7 — Source data Fig. 4 [file 44318_2024_291_MOESM7_ESM.zip › SD Figure 4/EMBOJ-2023-115976_Fig4A-10_Vinc.tif]

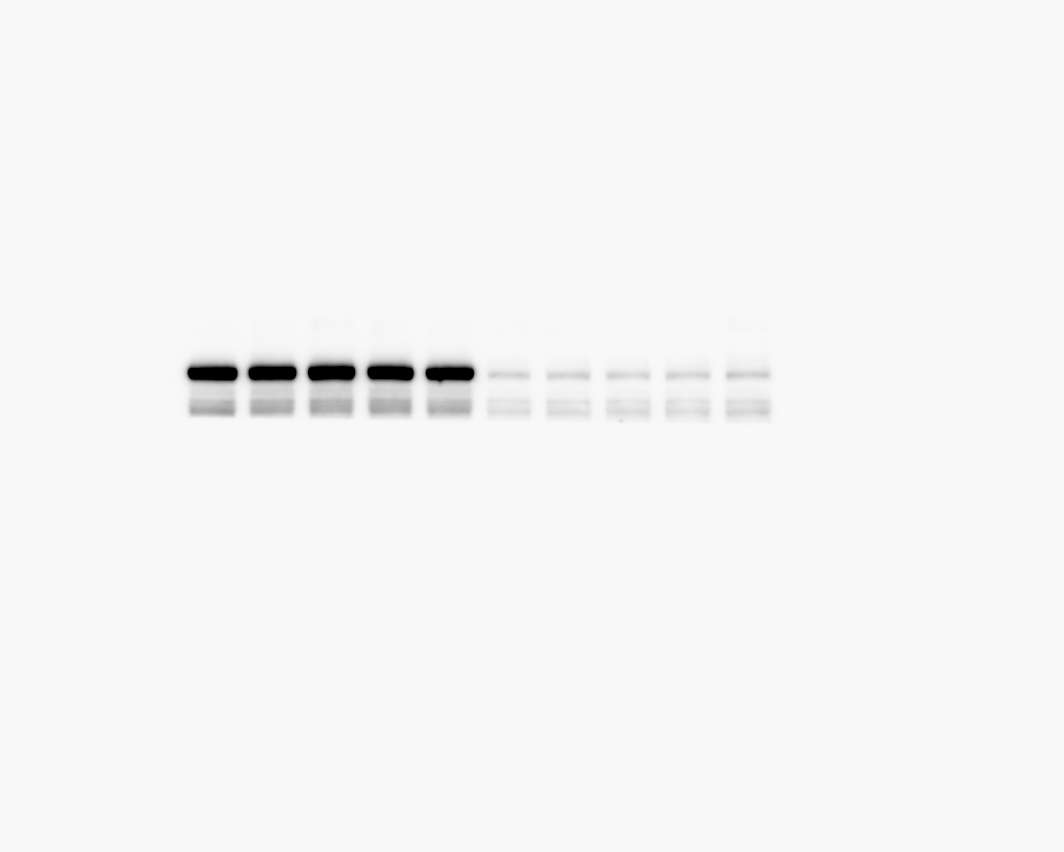

Supplement: Supplementary file 7 — Source data Fig. 4 [file 44318_2024_291_MOESM7_ESM.zip › SD Figure 4/EMBOJ-2023-115976_Fig4A-1_HOIP.tif]

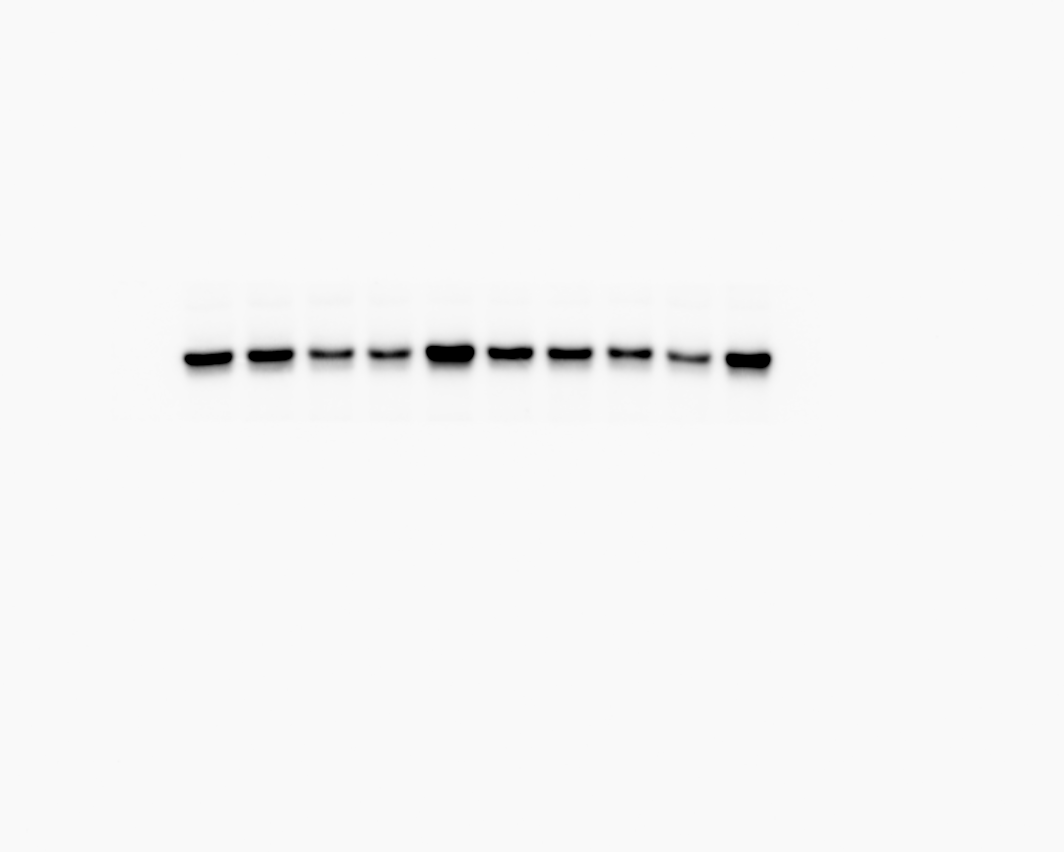

Supplement: Supplementary file 7 — Source data Fig. 4 [file 44318_2024_291_MOESM7_ESM.zip › SD Figure 4/EMBOJ-2023-115976_Fig4A-2_IkBa.tif]

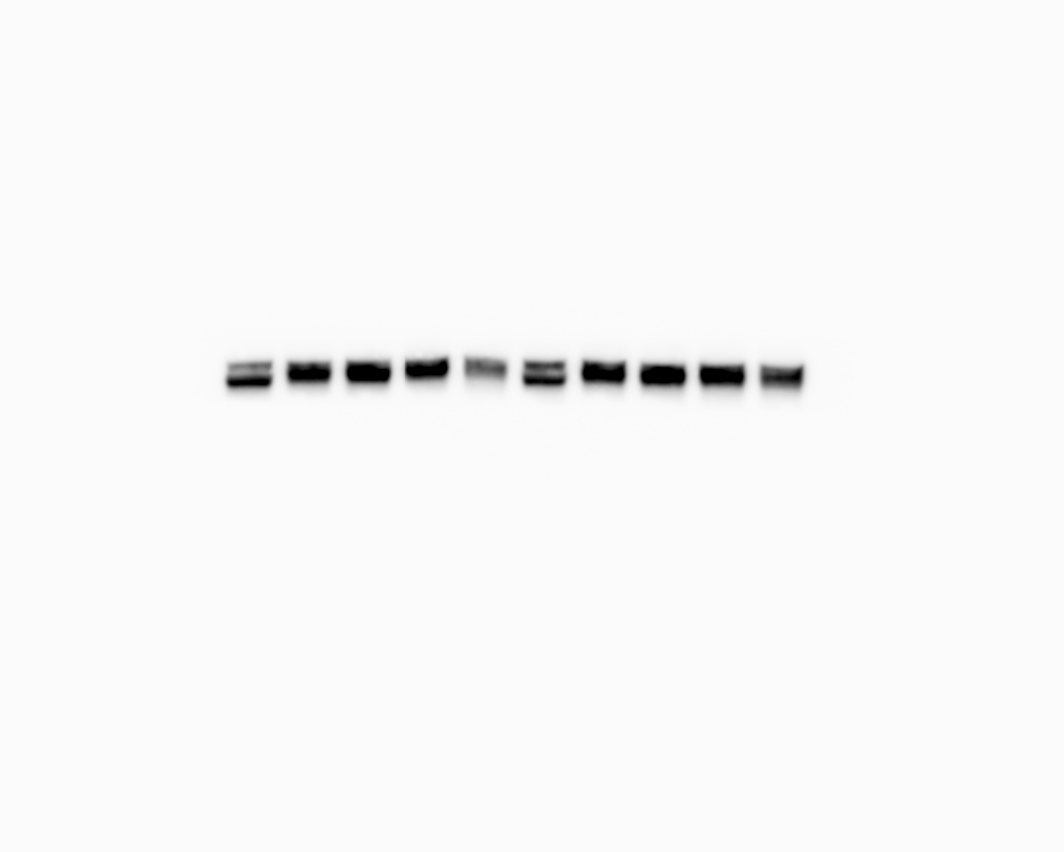

Supplement: Supplementary file 7 — Source data Fig. 4 [file 44318_2024_291_MOESM7_ESM.zip › SD Figure 4/EMBOJ-2023-115976_Fig4A-3_IRF3.tif]

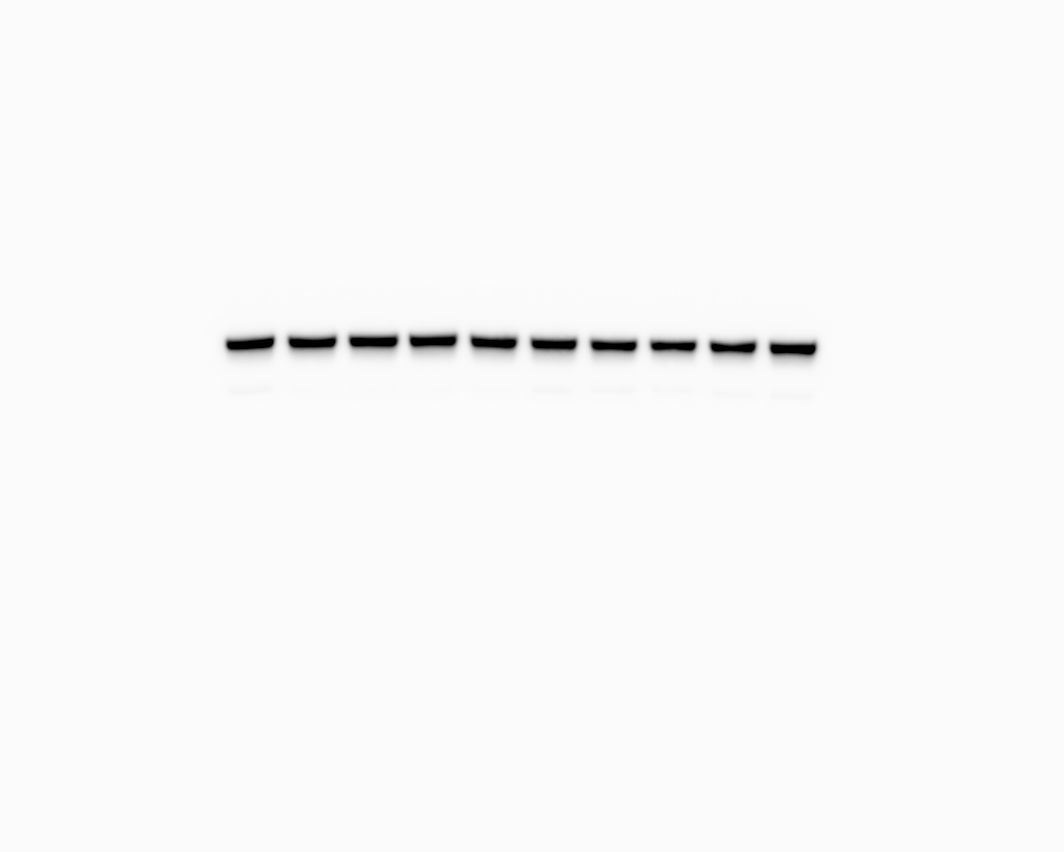

Supplement: Supplementary file 7 — Source data Fig. 4 [file 44318_2024_291_MOESM7_ESM.zip › SD Figure 4/EMBOJ-2023-115976_Fig4A-4_p65.tif]

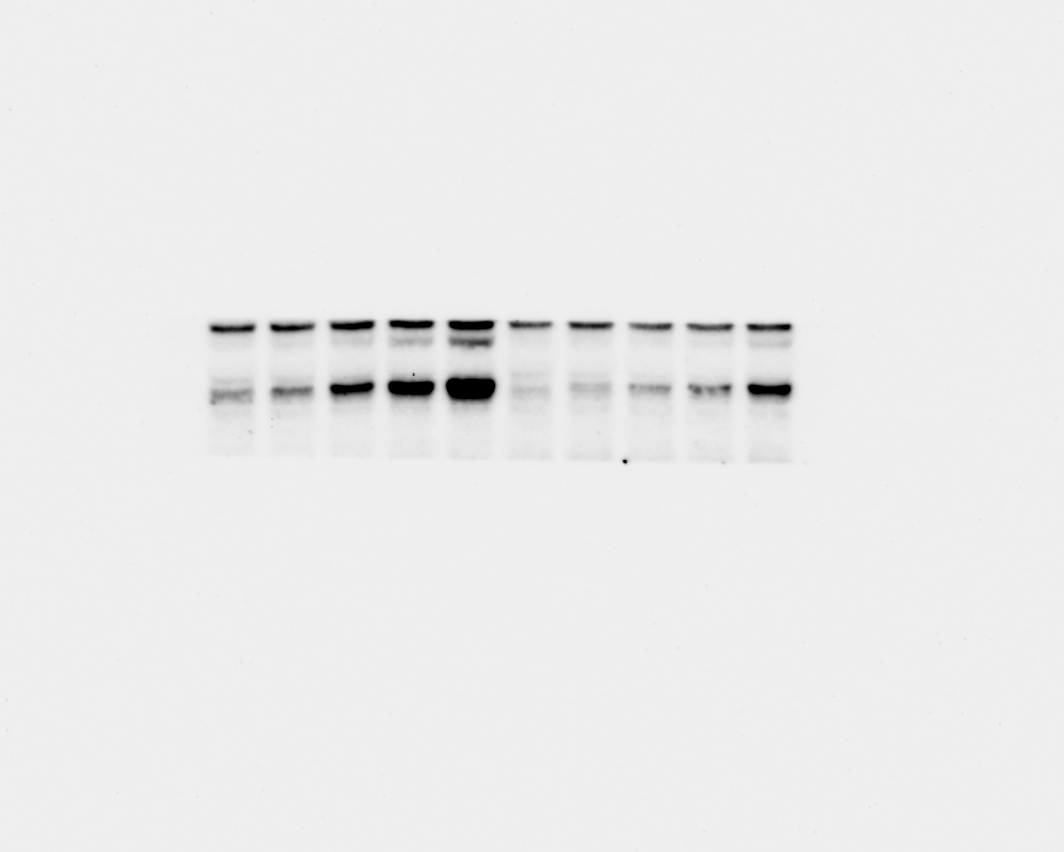

Supplement: Supplementary file 7 — Source data Fig. 4 [file 44318_2024_291_MOESM7_ESM.zip › SD Figure 4/EMBOJ-2023-115976_Fig4A-5_pIkBa.tif]

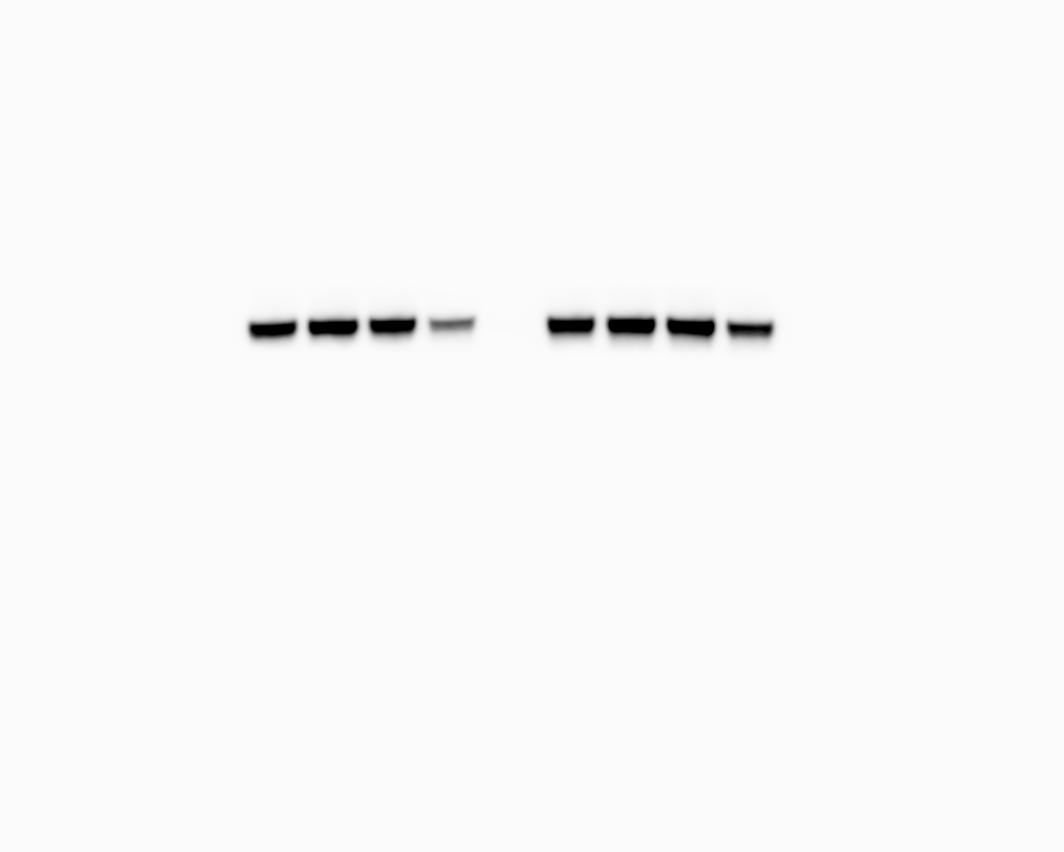

Supplement: Supplementary file 7 — Source data Fig. 4 [file 44318_2024_291_MOESM7_ESM.zip › SD Figure 4/EMBOJ-2023-115976_Fig4A-6_pIRF3.tif]

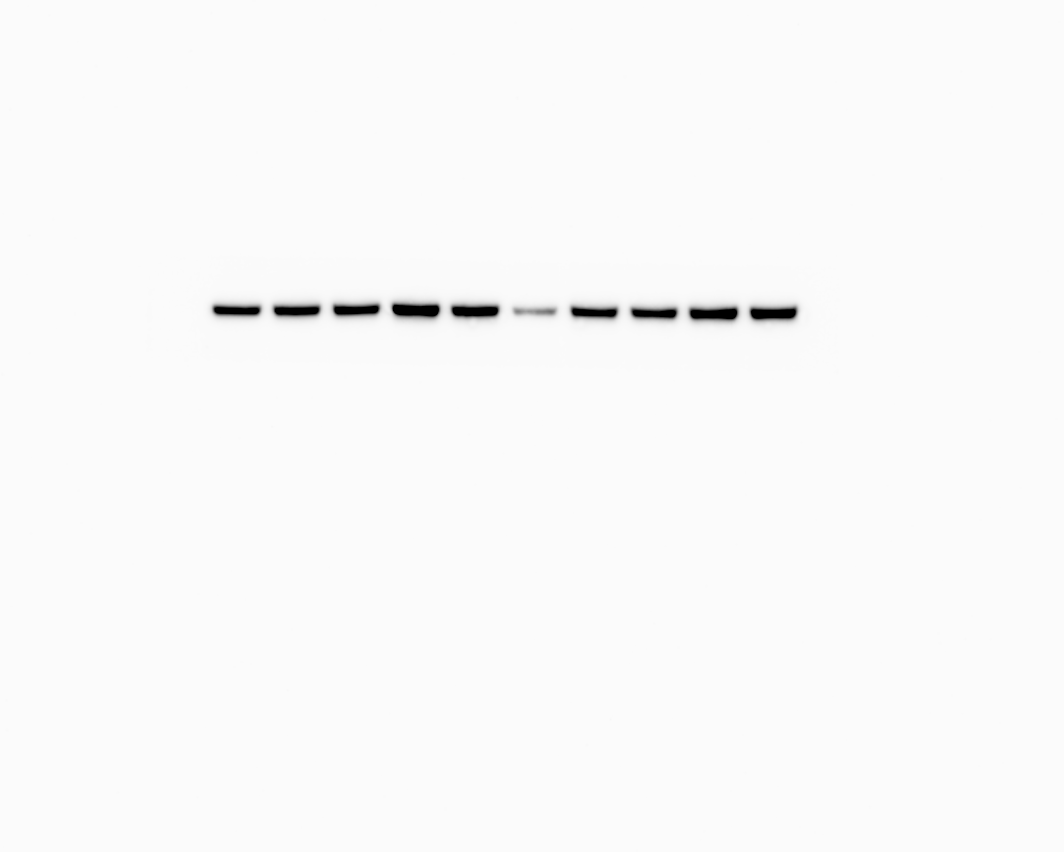

Supplement: Supplementary file 7 — Source data Fig. 4 [file 44318_2024_291_MOESM7_ESM.zip › SD Figure 4/EMBOJ-2023-115976_Fig4A-7_pp65.tif]

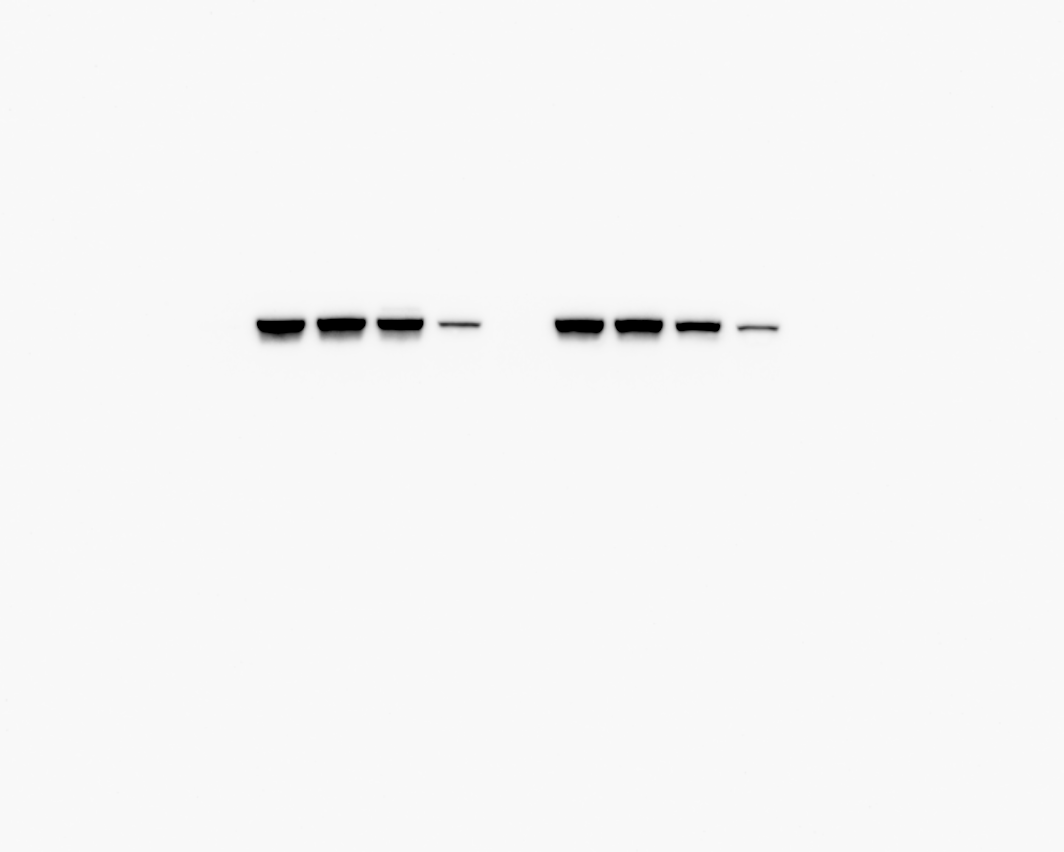

Supplement: Supplementary file 7 — Source data Fig. 4 [file 44318_2024_291_MOESM7_ESM.zip › SD Figure 4/EMBOJ-2023-115976_Fig4A-8_pTBK1.tif]

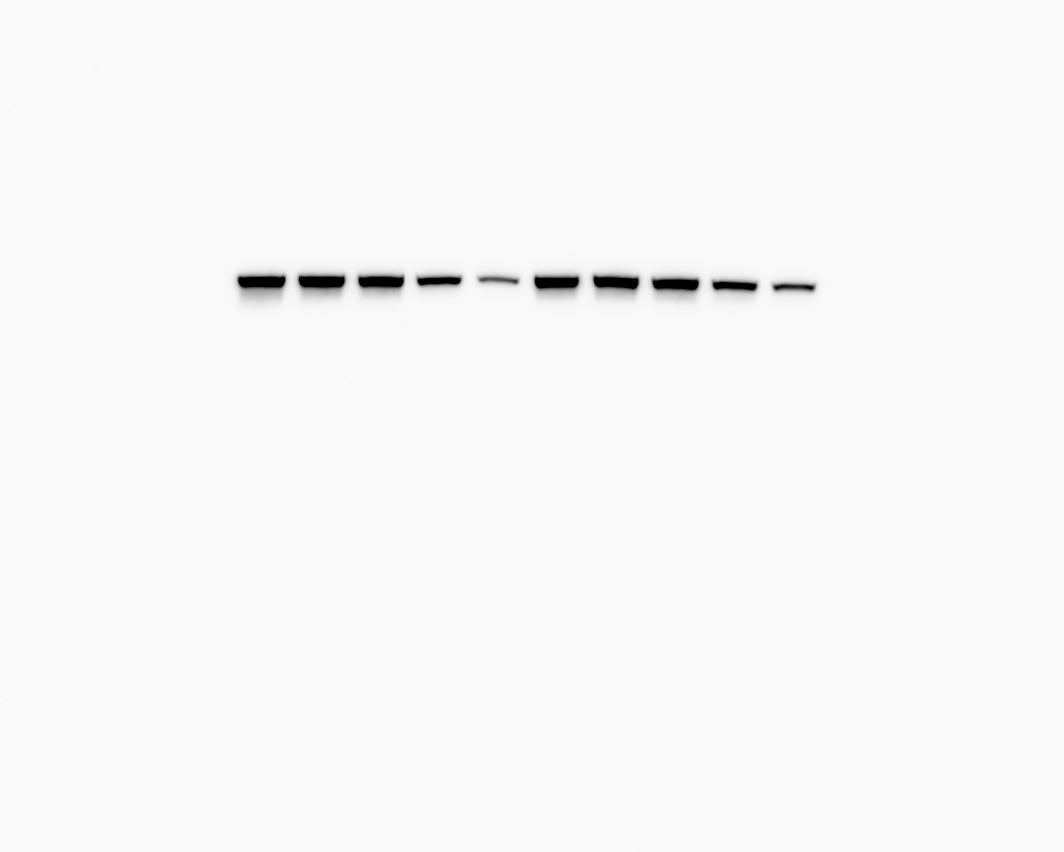

Supplement: Supplementary file 7 — Source data Fig. 4 [file 44318_2024_291_MOESM7_ESM.zip › SD Figure 4/EMBOJ-2023-115976_Fig4A-9_TBK1.tif]

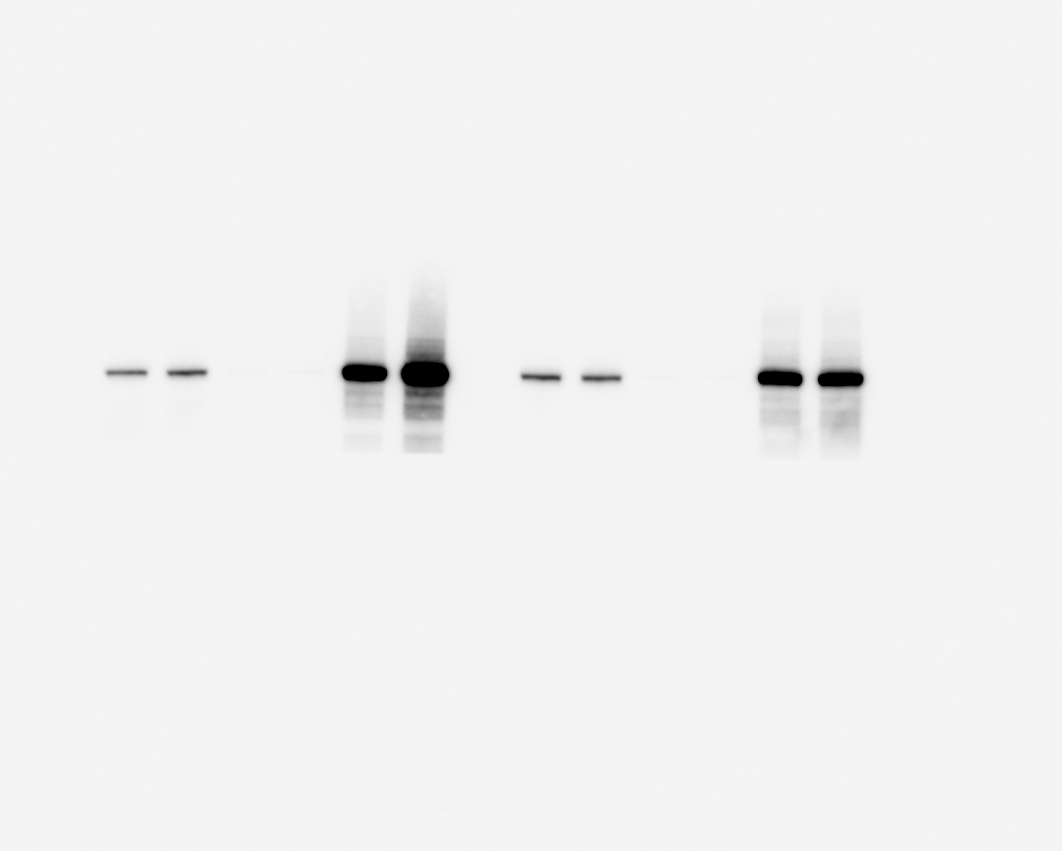

Supplement: Supplementary file 7 — Source data Fig. 4 [file 44318_2024_291_MOESM7_ESM.zip › SD Figure 4/EMBOJ-2023-115976_Fig4D-1_HOIP.tif]

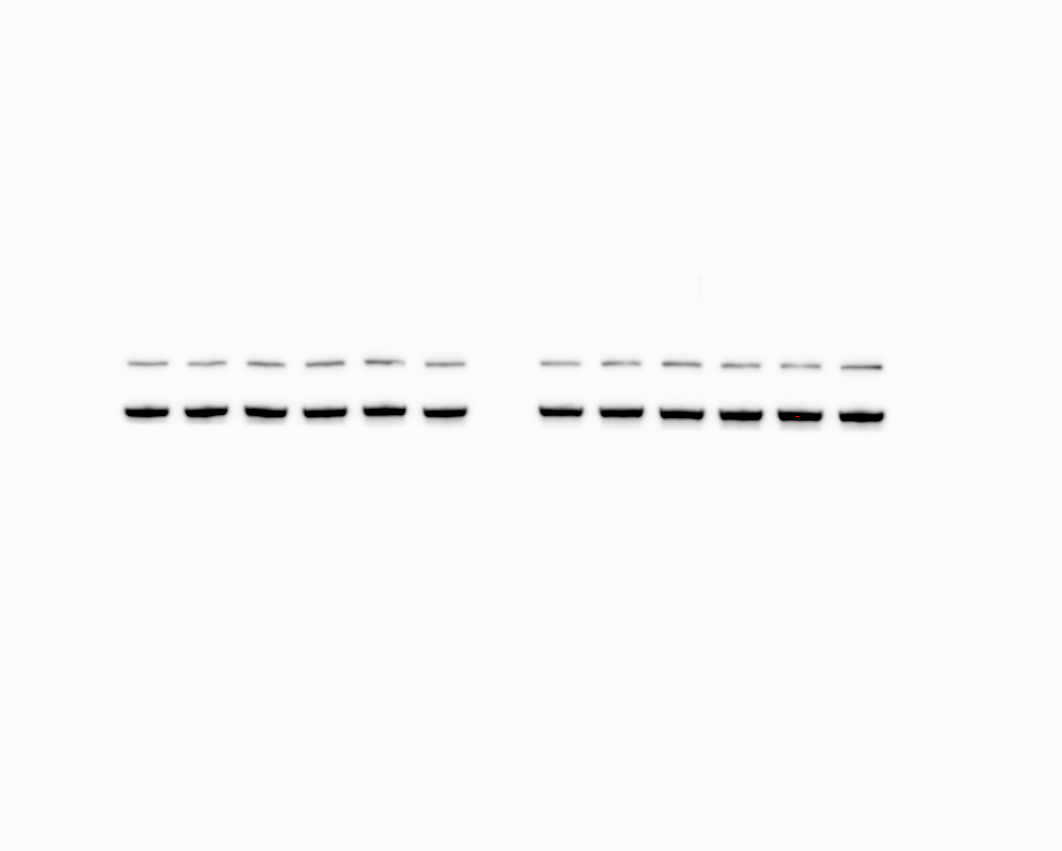

Supplement: Supplementary file 7 — Source data Fig. 4 [file 44318_2024_291_MOESM7_ESM.zip › SD Figure 4/EMBOJ-2023-115976_Fig4D-2_HSP90.tif]

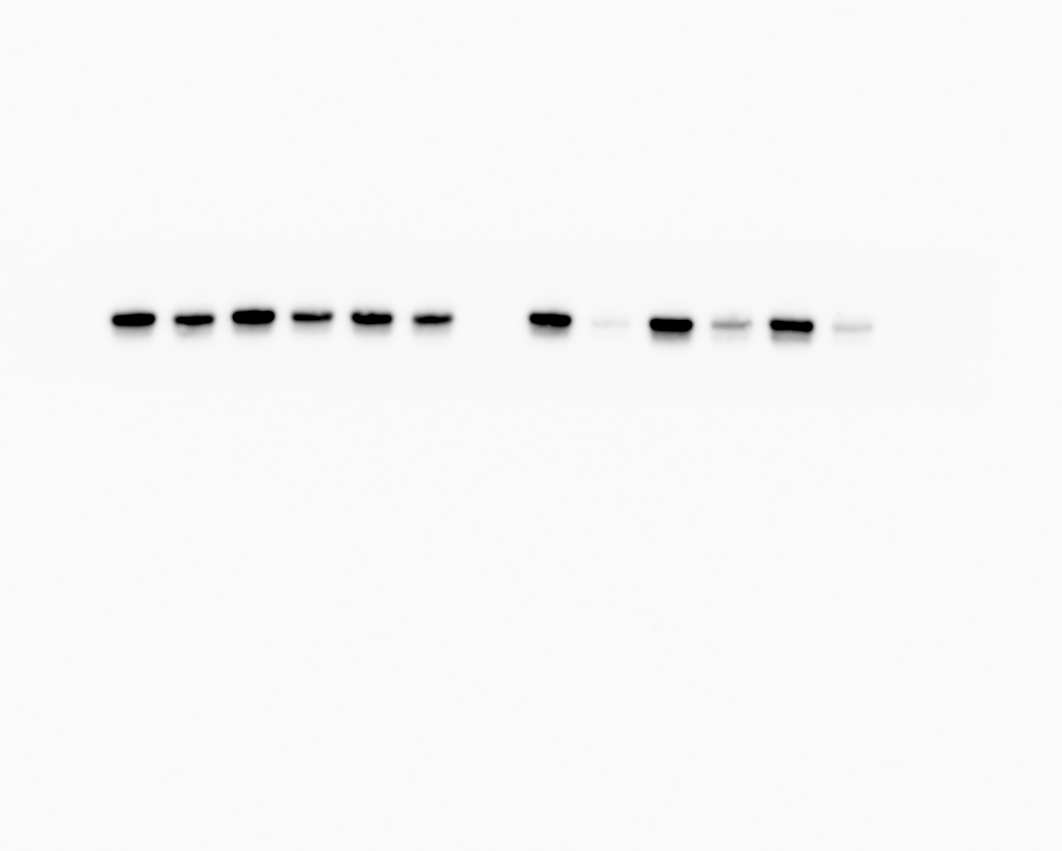

Supplement: Supplementary file 7 — Source data Fig. 4 [file 44318_2024_291_MOESM7_ESM.zip › SD Figure 4/EMBOJ-2023-115976_Fig4D-3_IkBa.tif]

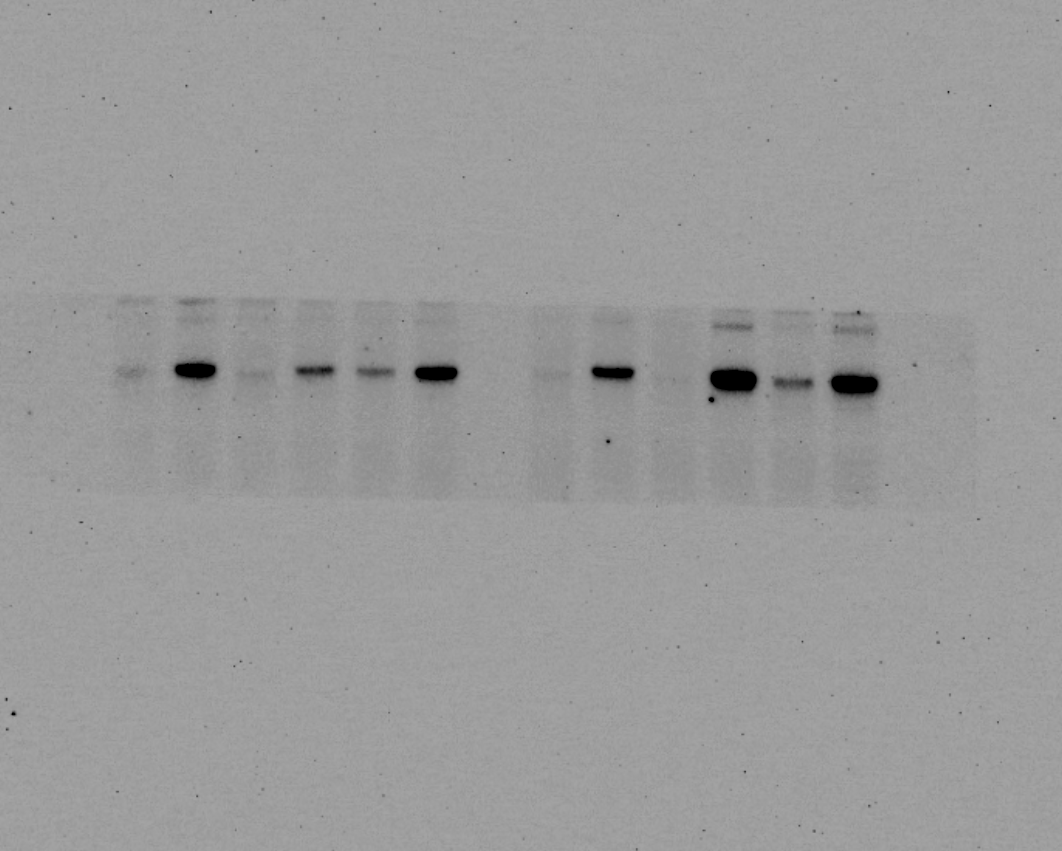

Supplement: Supplementary file 7 — Source data Fig. 4 [file 44318_2024_291_MOESM7_ESM.zip › SD Figure 4/EMBOJ-2023-115976_Fig4D-4_pIkBa.tif]

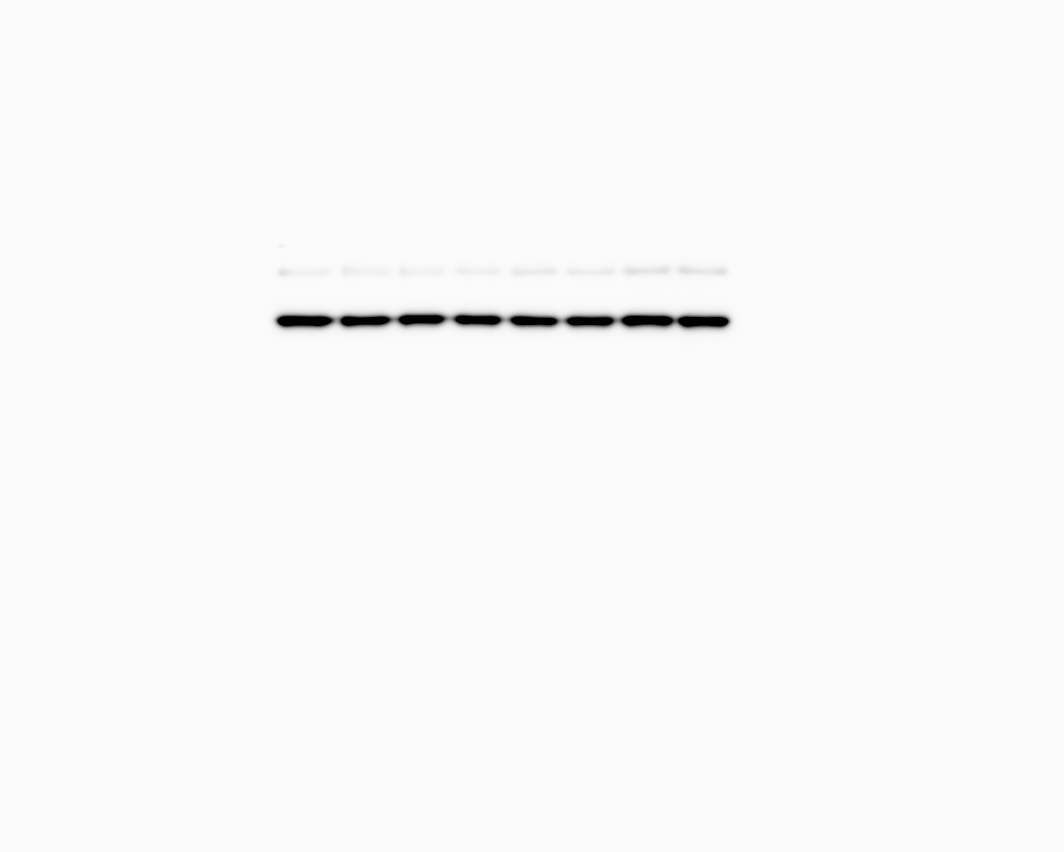

Supplement: Supplementary file 8 — Source data Fig. 5 [file 44318_2024_291_MOESM8_ESM.zip › SD Figure 5/EMBOJ-2023-115976_Fig5A-1_gapdh.tif]

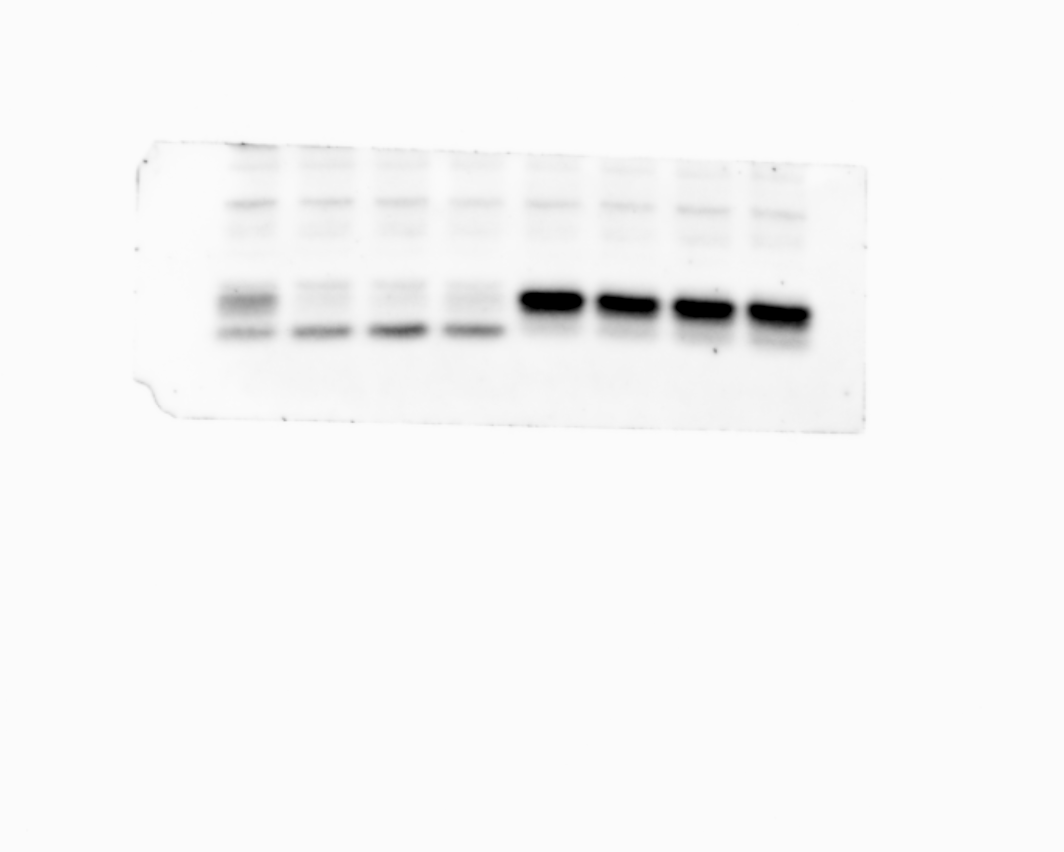

Supplement: Supplementary file 8 — Source data Fig. 5 [file 44318_2024_291_MOESM8_ESM.zip › SD Figure 5/EMBOJ-2023-115976_Fig5A-2_LC3B.tif]

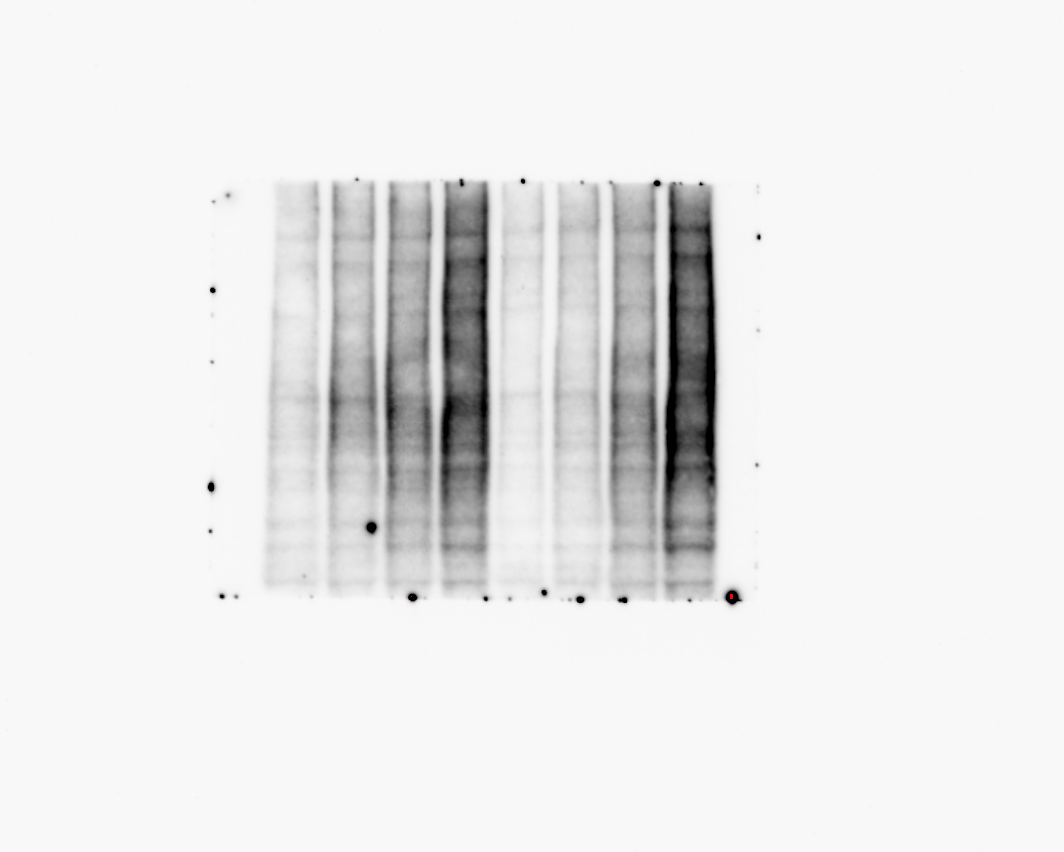

Supplement: Supplementary file 8 — Source data Fig. 5 [file 44318_2024_291_MOESM8_ESM.zip › SD Figure 5/EMBOJ-2023-115976_Fig5A-3_M1.tif]

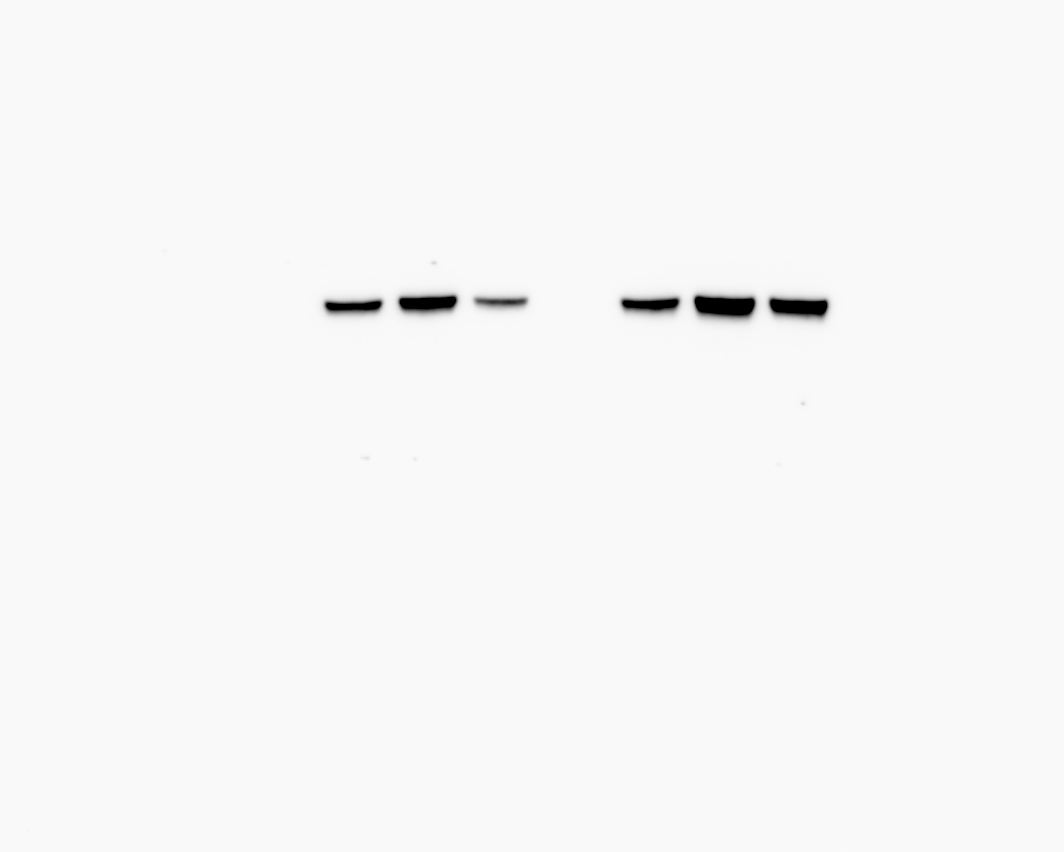

Supplement: Supplementary file 8 — Source data Fig. 5 [file 44318_2024_291_MOESM8_ESM.zip › SD Figure 5/EMBOJ-2023-115976_Fig5A-4_pSTING.tif]

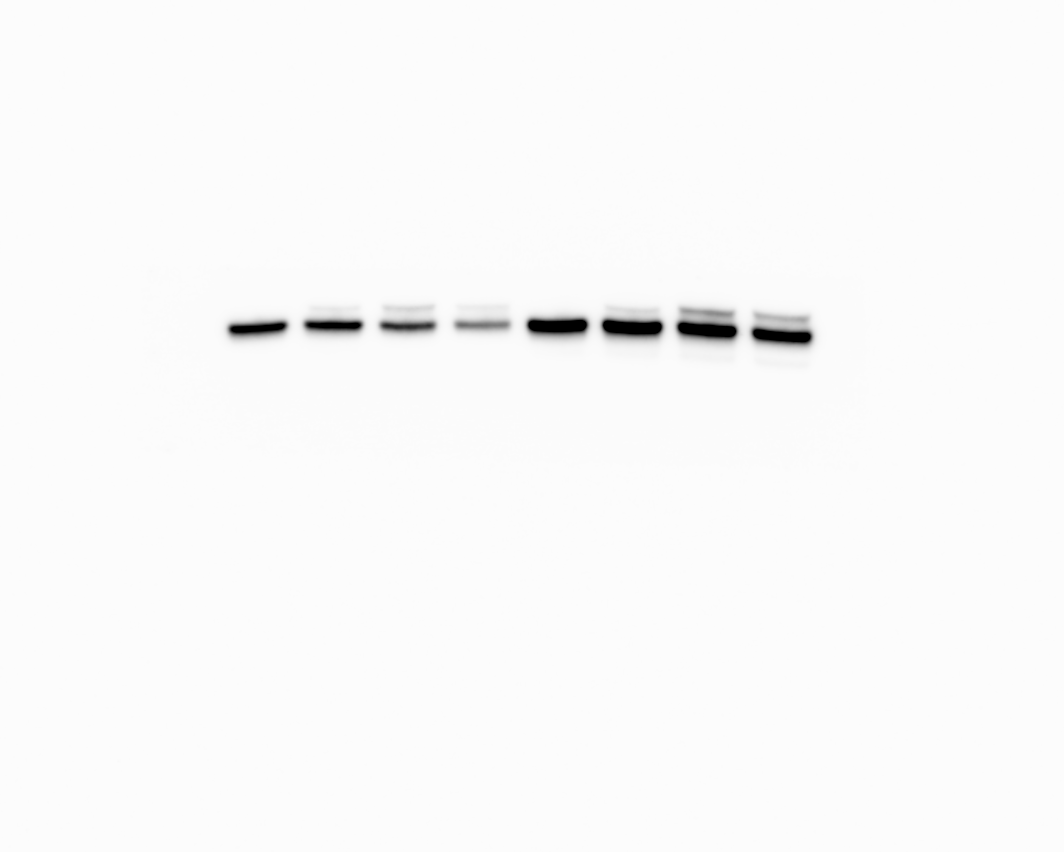

Supplement: Supplementary file 8 — Source data Fig. 5 [file 44318_2024_291_MOESM8_ESM.zip › SD Figure 5/EMBOJ-2023-115976_Fig5A-5_STING.tif]

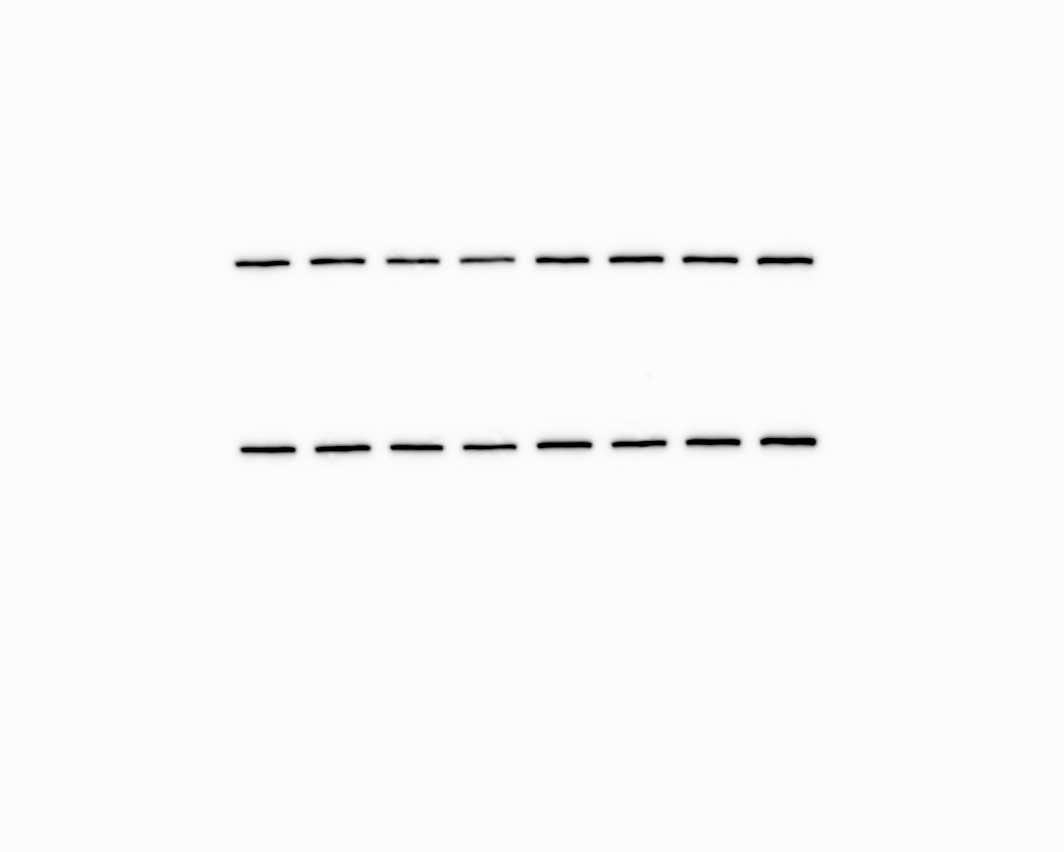

Supplement: Supplementary file 8 — Source data Fig. 5 [file 44318_2024_291_MOESM8_ESM.zip › SD Figure 5/EMBOJ-2023-115976_Fig5A-6_Vinc.tif]
