# Supplementary material for: STING induces HOIP-mediated synthesis of M1 ubiquitin chains to stimulate NF-κB signaling
Source: EMBO J. 2024 Nov 22;44(1):141–65. doi: 10.1038/s44318-024-00291-2 (PMC11696098; doi:10.1038/s44318-024-00291-2)
Supplement: Supplementary file 9 — Expanded View Figures [file 44318_2024_291_MOESM9_ESM.pdf]

Expanded View Figures

Figure EV1. Total-Ub and K63-Ub co-localization at LC3B foci following STING activation.

(**A**) Representative spinning disk confocal images of HeLa^{STING}; mEGFP-LC3B (green) cells treated with 120 µg/mL of cGAMP for 8 h prior to PFA-fixation and immunostaining for mono- and poly-ubiquitin chains (Ub; magenta), and STING (cyan). Scale bar = 20 µm. Corresponding to quantification in Fig. 1B. (**B**) Representative spinning disk confocal images of HeLa^{STING}; mEGFP-LC3B (green) cells treated with 1 µM diABZI for 4 h prior to PFA-fixation and immunostaining for mono- and poly-ubiquitin chains (Ub; magenta), and STING (cyan). Scale bar = 20 µm. Corresponding to quantification in Fig. EV1C. (**C**) Quantification of the percentage (%) of cells positive for mEGFP-LC3B foci and immunostained Ub foci (left panel), and the percentage (%) of mEGFP-LC3B foci with overlapping immunolabeled signal for Ub, STING, or both (right panel) from experiments represented in Fig. EV1B. Error bars represent ±s.d. from three replicates analyzed in the same experiment. Imaging was replicated in three independent experiments. (**D**, **E**) Representative spinning disk confocal images of HeLa^{STING}; mEGFP-LC3B cells treated with 120 µg/mL of cGAMP for 8 h prior to PFA-fixation and immunostaining for K48- and K63-ubiquitin chains. Scale bar = 20 µm. (**F**) Quantification of the percentage (%) of mEGFP-LC3B foci with overlapping signal for immunolabeled K48- and K63-ubiquitin chain from experiments represented in Fig. EV1D, E. Mean ± s.d. from $n = 3$ replicates analyzed in the same experiment. Imaging was replicated in three independent experiments. (**G**) Pearson's correlation coefficient of mScarletI-LC3B and Vx3-EGFP over time in the live imaging experiment represented in Movie EV1. FRT/TREX HeLa cells stably expressing FRT/TO-DD-Vx3-EGFP, BFP-P2A-STING, and mScarletI-LC3B were incubated with 1 µg/mL Doxycycline and 500 nM Shield1 for 24 h prior to treatment with either 120 µg/mL cGAMP or 1 µM diABZI and imaging every 30 min for 12 h on a spinning disk confocal microscope. Error bars represent ± s.d. from three replicates analyzed in the same experiment. Imaging was replicated in three independent experiments.

This is a U.S. Government work and not under copyright protection in the US; foreign copyright protection may apply

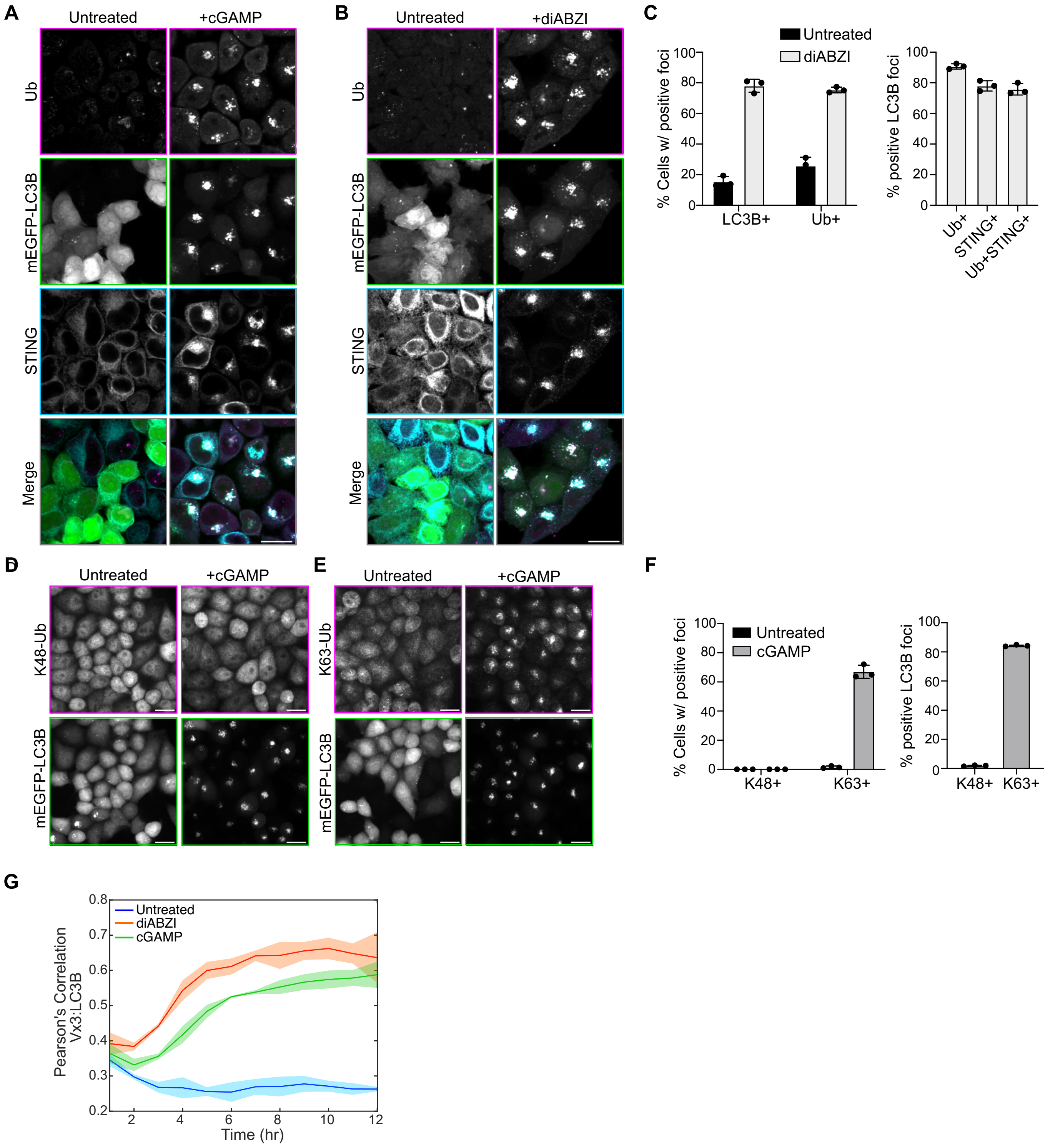

 This is a U.S. Government work and not under copyright protection in the US; foreign copyright protection may apply

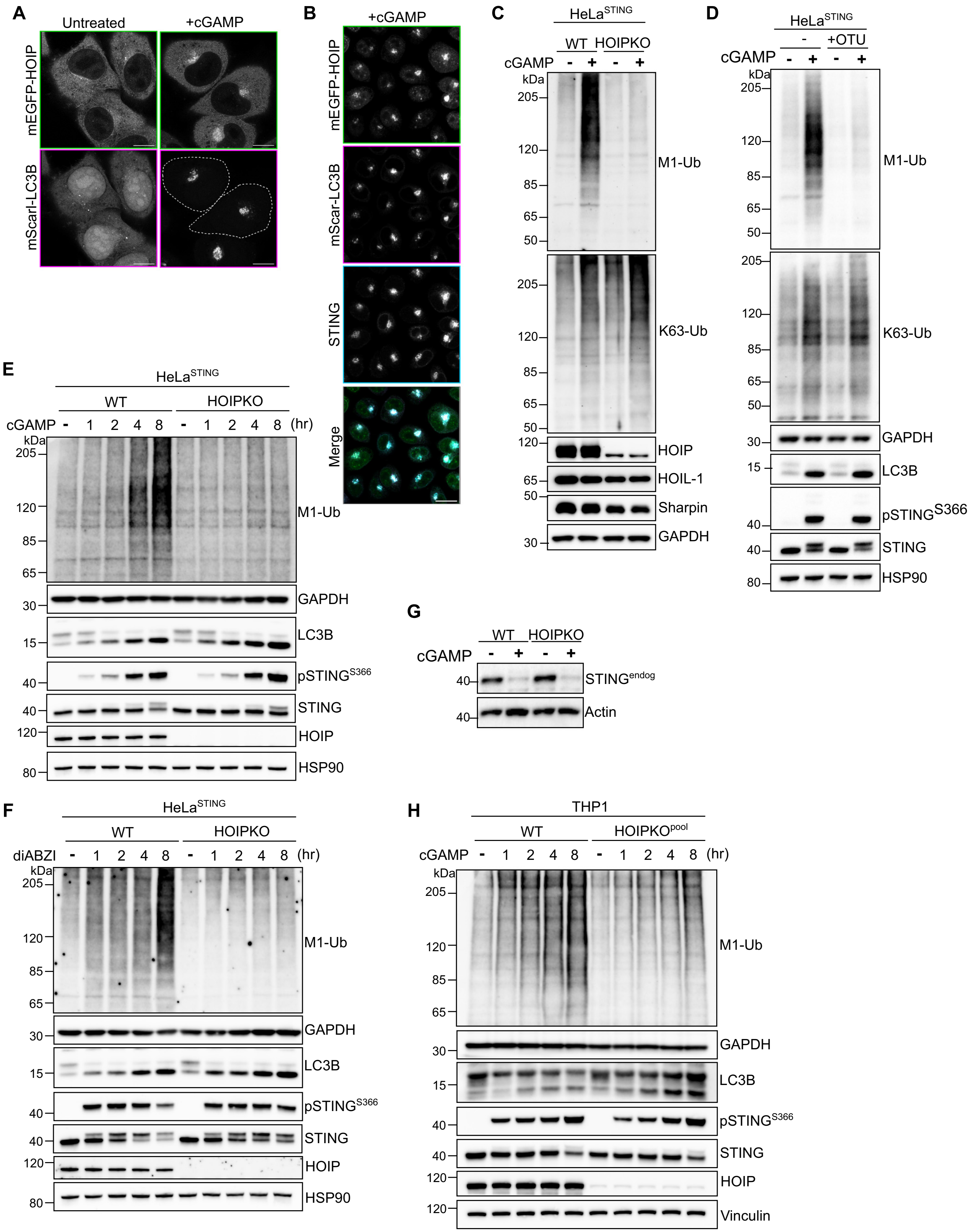
A
Untreated
+cGAMP
mEGFP-HOIP
mScarl-LC3B
B
+cGAMP
mEGFP-HOIP
mScarl-LC3B
STING
Merge
C
HeLaSTING
WT HOIPKO
cGAMP - + - +
kDa
M1-Ub
K63-Ub
HOIP
HOIL-1
Sharpin
GAPDH
D
HeLaSTING
- +OTU
cGAMP - + - +
M1-Ub
K63-Ub
GAPDH
LC3B
pSTINGS366
STING
HSP90
E
HeLaSTING
WT HOIPKO
cGAMP - 1 2 4 8 - 1 2 4 8 (hr)
M1-Ub
GAPDH
LC3B
pSTINGS366
STING
HOIP
HSP90
G
WT HOIPKO
cGAMP - + - +
STINGendog
Actin
F
HeLaSTING
WT HOIPKO
diABZI - 1 2 4 8 - 1 2 4 8 (hr)
M1-Ub
GAPDH
LC3B
pSTINGS366
STING
HOIP
HSP90
H
THP1
WT HOIPKOpool
cGAMP - 1 2 4 8 - 1 2 4 8 (hr)
M1-Ub
GAPDH
LC3B
pSTINGS366
STING
HOIP
Vinculin

Figure EV2. STING activation by multiple agonists induces HOIP-mediated M1 ubiquitin chain formation, which is not required for STING degradation or LC3B lipidation in HeLa or THP1 cells.

(**A**) Representative Airyscan-processed confocal images of $HeLa^{STING}$ cells stably expressing mScarletI-LC3B and mEGFP-HOIP treated with 120 µg/mL of cGAMP for 8 h with no saponin extraction prior to PFA-fixation. Scale bar = 10 µm. Corresponding to representative images in Fig. 2A. Imaging was replicated in three independent experiments. (**B**) Representative spinning disk confocal images of $HeLa^{STING}$; mEGFP-HOIP (green); mScarletI-LC3B (magenta) cells treated with 120 µg/mL of cGAMP for 8 h prior to prior to saponin extraction and PFA-fixation. Scale bar = 20 µm. Corresponding to quantification in Fig. 2B. (**C**, **D**) Representative immunoblots of indicated proteins detected in cell lysates from $HeLa^{STING}$ WT and HOIPKO cells (**C**) or $HeLa^{STING}$ and $HeLa^{STING}$ stably expressing mEGFP-OTULIN (**D**) prepared following treatment with 120 µg/mL cGAMP for 8 h. Immunoblotting was replicated in three independent experiments. (**E**, **F**) Representative immunoblots of indicated proteins detected in $HeLa^{STING}$ cell lysates from WT and HOIPKO cells prepared following treatment with 120 µg/mL cGAMP (**E**) or 1 µM diABZI (**F**) for 1, 2, 4, and 8 h. Immunoblotting was replicated in three independent experiments. (**G**) Representative immunoblots of endogenous STING were detected in cell lysates from WT and HOIPKO HeLa cells without stable overexpression of STING. Cells were treated with 15 µg/mL cGAMP for 8 h. Immunoblotting was replicated in three independent experiments. (**H**) Representative immunoblots of indicated proteins detected in THP1 cell lysates from WT and $HOIPKO^{pool}$ cells prepared following treatment with 120 µg/mL cGAMP for 1, 2, 4, and 8 h. Immunoblotting was replicated in three independent experiments.

This is a U.S. Government work and not under copyright protection in the US; foreign copyright protection may apply

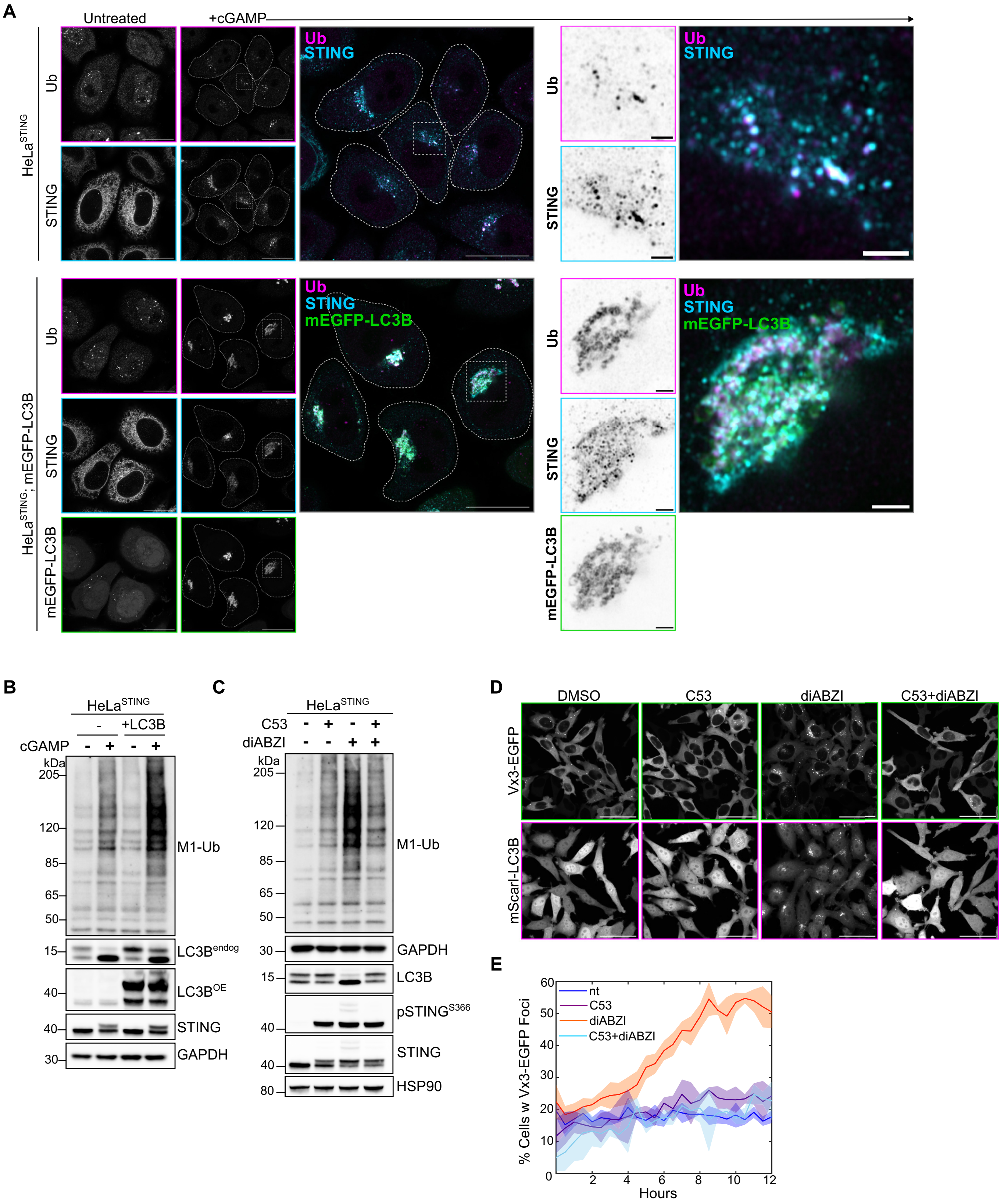
A
Untreated
+cGAMP
HeLaSTING
Ub
STING
HeLaSTING, mEGFP-LC3B
mEGFP-LC3B
Ub
STING
mEGFP-LC3B
B
HeLaSTING
- +LC3B
cGAMP - + - +
kDa
205
120
85
65
50
15
40
40
30
M1-Ub
LC3Bendog
LC3BOE
STING
GAPDH
C
HeLaSTING
C53 - + - +
diABZI - - + +
kDa
205
120
85
65
50
30
15
40
40
80
M1-Ub
GAPDH
LC3B
pSTINGS366
STING
HSP90
D
DMSO
C53
diABZI
C53+diABZI
Vx3-EGFP
mScarl-LC3B
E
nt
C53
diABZI
C53+diABZI
% Cells w Vx3-EGFP Foci
Hours
0
10
20
30
40
50
60
2
4
6
8
10
12

Figure EV3. Overexpression of LC3B affects STING activation-induced ubiquitylation, which may involve the Golgi neutralizing function of STING.

(**A**) Representative Airyscan-processed confocal images of $HeLa^{STING}$ and $HeLa^{STING}$ cells with stable overexpression of mEGFP-LC3B (green) treated with 120 μg/mL of cGAMP for 8 h prior to PFA-fixation and immunostaining with antibodies raised against mono- and poly-ubiquitin chains (Ub; magenta) and STING (cyan). Scale bar = 20 and 2 μm (inset). Imaging was replicated in two independent experiments. (**B**) Representative immunoblots of indicated proteins detected lysates from $HeLa^{STING}$ WT and $HeLa^{STING}$ with stable overexpression of mEGFP-LC3B prepared after treatment with 120 μg/mL cGAMP for 8 h. Immunoblotting was replicated in 3 independent experiments. (**C**) Representative immunoblots of indicated proteins detected in $HeLa^{STING}$ cell lysates prepared after treatment with either DMSO, 10 μM C53, 1 μM diABZI, or both C53 and diABZI for 4 h. Immunoblotting was replicated in three independent experiments. (**D**, **E**) Representative spinning disk confocal images of FRT/TREX HeLa cells stably expressing FRT/TO-DD-Vx3-EGFP, BFP-P2A-STING, and mScarletI-LC3B at the 6-hour timepoint following treatment (**D**) and quantification of the percentage (%) of cells positive for Vx3-EGFP foci over time (**E**). Cells were incubated with 1 μg/mL Doxycycline and 500 nM Shield1 for 24 h prior to treatment with either DMSO, 10 μM C53, 1 μM diABZI, or both C53 and diABZI, and imaging every 30 min for 12 h on a spinning disk confocal microscope. Scale bar = 50 μm. Quantification is from three wells analyzed in the same experiment. Imaging was replicated in two independent experiments.

This is a U.S. Government work and not under copyright protection in the US; foreign copyright protection may apply

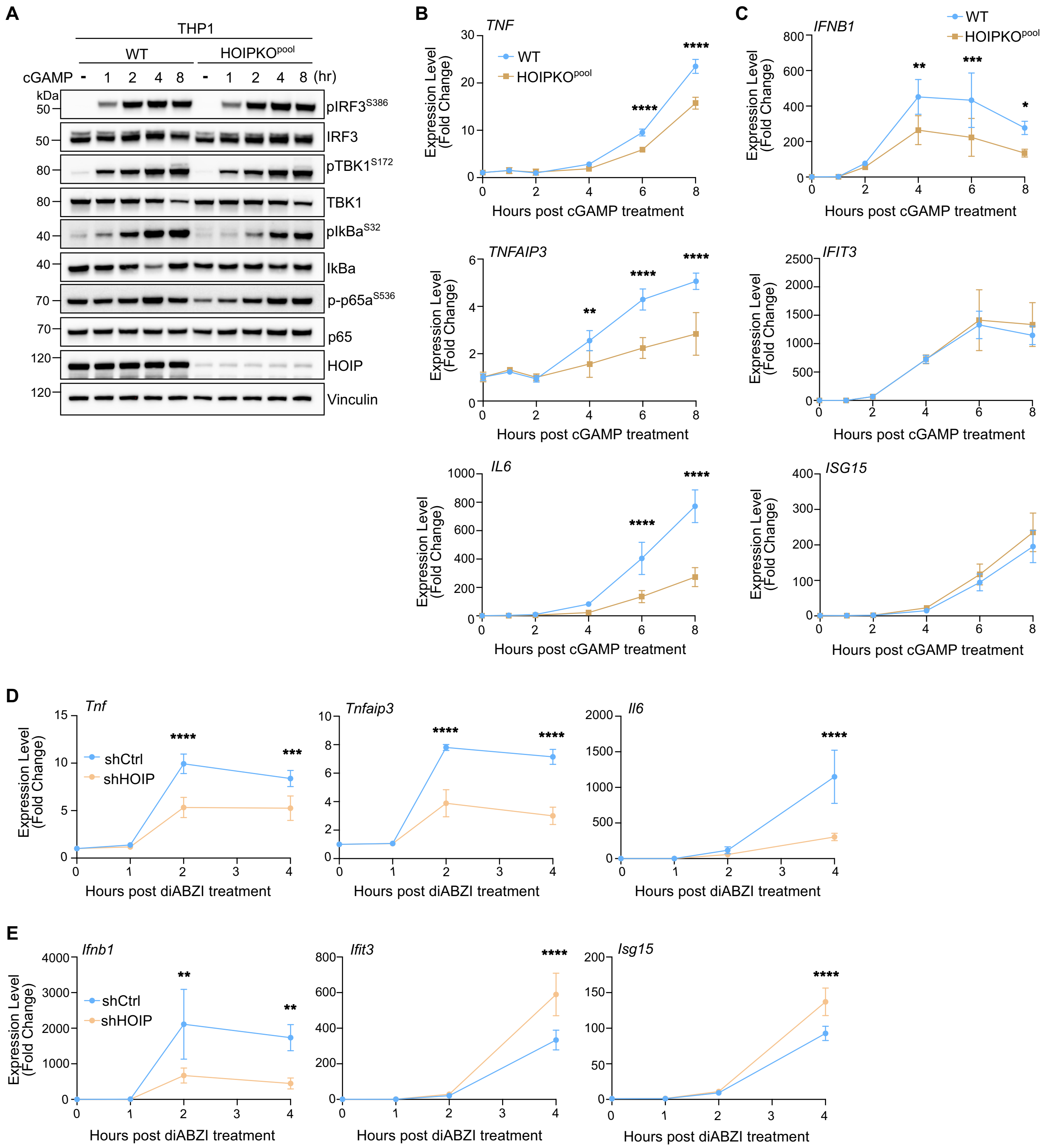

This is a U.S. Government work and not under copyright protection in the US; foreign copyright protection may apply

Figure EV4. HOIP is important for NFκB signaling following STING activation by cGAMP and in mouse bone marrow-derived macrophages.

(**A**) Representative immunoblots of indicated proteins detected in THP1 cell lysates from WT and HOIPKO^{pool} cells prepared following treatment with 120 μg/mL cGAMP for 1, 2, 4, and 8 h. Immunoblotting was replicated in three independent experiments. (**B**, **C**) Relative expression changes of indicated NFκB- (**B**) and IRF3/interferon-related (**C**) genes detected by quantitative RT-PCR in WT and HOIPKO^{pool} THP1 cells treated with 120 μg/mL cGAMP for 1, 2, 4, and 8 h. Quantification of relative expression is from three independent experiments analyzed at the same time. A two-way ANOVA with Sidak's multiple comparisons test was performed on $2^{-\Delta\Delta Ct}$ values. Mean ± s.d. $n = 3$ *<0.05, **<0.01, ***<0.001, ****<0.0001 (*TNF* 6 h $p =$ <0.0001, 8 h $p =$ <0.0001; *TNFAIP3* 4 h $p = 0.0084$, 6 h $p =$ <0.0001, 8 h $p =$ <0.0001; *IL6* 6 h $p =$ <0.0001, 8 h $p =$ <0.0001; *IFNB1* 2 h $p = 0.0019$, 4 h $p = 0.0005$, 8 h $p = 0.0281$). (**D**, **E**) Relative expression changes of indicated NFκB- (**D**) and IRF3/interferon-related (**E**) genes detected by quantitative RT-PCR in WT and shCtrl and shHOIP iBMDM cells treated with 0.2 μM diABZI for 1, 2, and 4 h. Quantification of relative expression is from three independent experiments analyzed at the same time. A two-way ANOVA with Sidak's multiple comparisons test was performed on $2^{-\Delta\Delta Ct}$ values. Mean ± s.d. $n = 3$ **<0.01, ***<0.001, ****<0.0001 (*Tnf* 2 h $p =$ <0.0001, 4 h $p = 0.0005$; *Tnfaip3* 2 h $p =$ <0.0001, 4 h $p =$ <0.0001; *Il6* 4 h $p =$ <0.0001; *Ifnb1* 2 h $p = 0.0011$, 4 h $p = 0.0031$; *Ifit3* 4 h $p =$ <0.0001; *Isg15* 4 h $p =$ <0.0001).

This is a U.S. Government work and not under copyright protection in the US; foreign copyright protection may apply

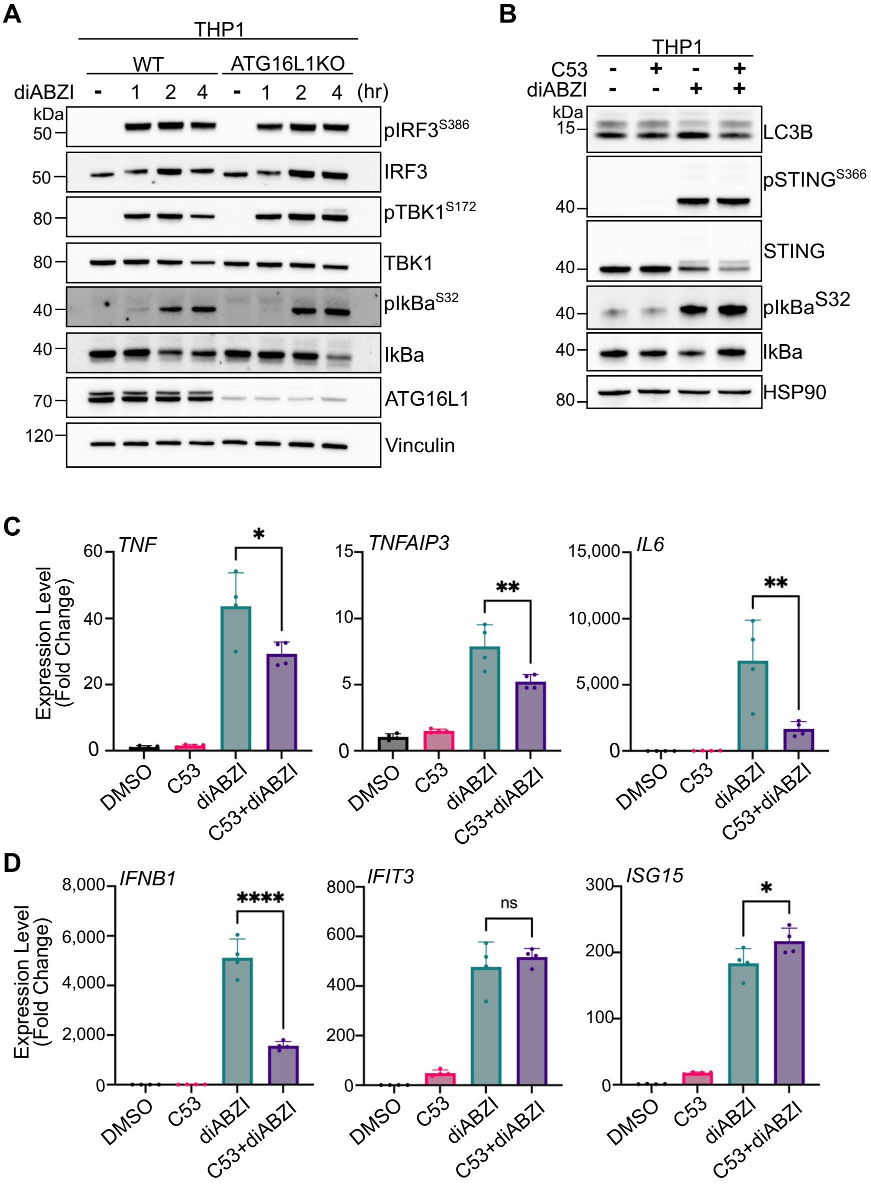

Figure EV5. ATG16L1 is not required for IRF3- and NFκB-related signaling induced by STING activation, however the upstream Golgi neutralizing function of STING may affect immune-related gene expression.

(**A**) Representative immunoblots of indicated proteins detected in THP1 cell lysates from WT and ATG16L1KO cells prepared following treatment with 1 µM diABZI for 1, 2, and 4 h. Immunoblotting was replicated in three independent experiments. (**B**) Representative immunoblots of indicated proteins detected in lysates from WT THP1 cells prepared following treatment with either DMSO, 10 µM C53, 1 µM diABZI, or both C53 and diABZI for 4 h. Immunoblotting was replicated in three independent experiments. (**C**, **D**) Relative expression changes of indicated NFκB-related genes (**C**) and interferon-related genes (**D**) detected by quantitative RT-PCR in THP1 cells treated with DMSO, 10 µM C53, 1 µM diABZI, or both C53 and diABZI for 4 h. Quantification of relative expression is from four independent experiments analyzed at the same time. A one-way ANOVA with Tukey's multiple comparisons test was performed on $2^{-\Delta\Delta Ct}$ values. Mean ± s.d. $n = 4$ *<0.05, **<0.01, ****<0.0001 (*TNF* $p = 0.0123$; *TNFAIP3* $p = 0.005$; *IL6* $p = 0.0027$; *IFNB1* $p = <0.0001$; *ISG15* $p = 0.0351$).

This is a U.S. Government work and not under copyright protection in the US; foreign copyright protection may apply
